# Synthesis and preclinical evaluation of tigilanol tiglate analogs as latency-reversing agents for the eradication of HIV

**DOI:** 10.1126/sciadv.ads1911

**Published:** 2025-01-24

**Authors:** Zachary O. Gentry, Owen D. McAteer, Jennifer L. Hamad, Jose A. Moran, Jocelyn T. Kim, Matthew D. Marsden, Jerome A. Zack, Paul A. Wender

**Affiliations:** ^1^ Department of Chemistry, Stanford University, Stanford, CA 94305, USA.; ^2^ Department of Biology, Stanford University, Stanford, CA 94305, USA.; ^3^ Department of Microbiology & Molecular Genetics, School of Medicine, University of California Irvine, Irvine, CA 92697, USA.; ^4^ Department of Medicine, Division of Infectious Diseases, University of California Los Angeles, Los Angeles, CA 90095, USA.; ^5^ Department of Medicine (Division of Infectious Diseases), School of Medicine, University of California Irvine, Irvine, CA 92697, USA.; ^6^ Department of Microbiology, Immunology, and Molecular Genetics, University of California Los Angeles, Los Angeles, CA 90095, USA.; ^7^ Department of Medicine, Division of Hematology and Oncology, University of California Los Angeles, Los Angeles, CA 90095, USA.; ^8^ Department of Chemical and Systems Biology, Stanford University, Stanford, CA 94305, USA.

## Abstract

Tigilanol tiglate (EBC-46) is a selective modulator of protein kinase C (PKC) isoforms that is Food and Drug Administration (FDA) approved for the treatment of mast cell tumors in canines with up to an 88% cure rate. Recently, it has been FDA approved for the treatment of soft tissue sarcomas in humans. The role of EBC-46 and, especially, its analogs in efforts to eradicate HIV, treat neurological and cardiovascular disorders, or enhance antigen density in antigen-targeted chimeric antigen receptor–T cell and chimeric antigen receptor–natural killer cell immunotherapies has not been reported. Enabled by our previously reported scalable synthesis of EBC-46, we report herein the systematic design, synthesis, and evaluation of EBC-46 analogs, including those inaccessible from the natural source and their PKC affinities, ability to translocate PKC, nuclear factor κB activity, and efficacy in reversing HIV latency in Jurkat-Latency cells. Leading analogs show exceptional PKC affinities, isoform selectivities, and functional activities, serving as promising candidates for therapeutic applications.

## Teaser

Tigilanol tiglate analogs exhibit 90% latency reversal, marking unprecedented progress toward HIV eradication.

## INTRODUCTION

Approximately 39 million people are living with HIV globally (*1*). While current antiretroviral therapies (ARTs) can suppress viral loads to undetectable levels in people living with HIV (*2*), ART is not a cure as it does not eliminate reservoirs of latently infected cells that chronically resupply the virus. As a result, people living with HIV require lifelong ART treatment, which raises concerns about compliance, resistance, cost, and health issues associated with chronic exposure (*3*, *4*). While global access to ART is improving, supply has still not met the need. To circumvent the concerns arising from chronic therapies, strategies to cure HIV are being pursued, including gene therapies to remove the HIV-integrated virus, immune therapies to clear infected cells, “block and lock” strategies to silence the latent virus, and “kick and kill” strategies using latency-reversing agents (LRAs) to activate (kick) HIV-infected cells, allowing for their clearance (kill) by the immune system or other approaches that target cells actively expressing HIV proteins (*5*–*9*).

**Fig. 1.**
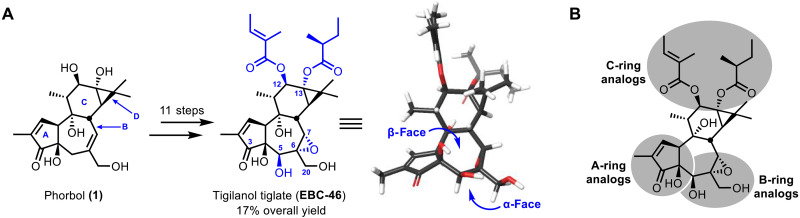
Phorbol as a source of EBC-46 and its analogs. (**A**) Overview of the EBC-46 synthesis from phorbol and (**B**) overview of EBC-46 analog design.

**Fig. 2.**
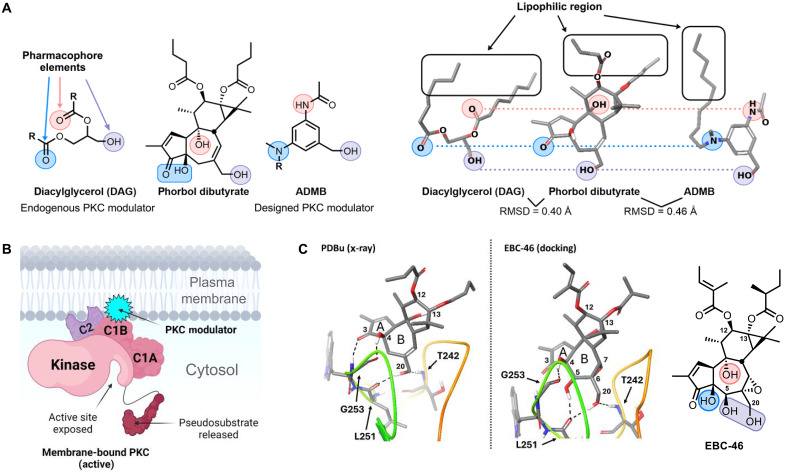
Design of EBC-46 analogs. (**A**) Comparison of the pharmacophoric elements of (*S*)-diacylglycerol (DAG), phorbol dibutyrate (PDBu), and 3-(*N*-acetylamino)-5-(*N*-decyl-*N*-methylamino)benzylalcohol (ADMB). RMSD, root mean square deviation. (**B**) Model of the membrane-ligand-PKC ternary complex. (**C**) Three-dimensional x-ray crystal structure of PDBu bound to the C1b domain of PKC-δ (left) and a docking model of EBC-46 bound to the C1b domain of PKC-δ (right) (key amino acid residues labeled and ligand-protein hydrogen bonds indicated by dotted black lines). This figure was created with BioRender.

“Kick and kill” studies have uncovered several classes of LRAs, including protein kinase C (PKC) modulators (*9*), histone deacetylase and bromodomain inhibitors (*10*), Toll-like receptor agonists (*11*), disulfiram (*12*), benzotriazoles (*10*), SMAC mimetics (*10*), and Tat-mRNA LNPs (*13*), as well as LRA combinations that have been reported to exhibit synergistic effects (*14*). PKC modulators have shown special promise as LRAs (*10*, *15*–*21*), including a recent report of delayed virologic rebound upon treatment interruption in a humanized mouse model using an analog of bryostatin 1 as the “kick” (LRA) component and natural killer cells as the “kill” component (*8*). Prompted by this progress and our unique scalable synthesis of EBC-46 (Fig. 1A) (*22*), a PKC modulator approved for veterinary medicine and human use for cancer (*23*), we initiated studies, as described herein, to determine whether EBC-46 precursors and derivatives (Fig. 1B), incorporating our previously described PKC binding features (pharmacophore requirements) (Fig. 2A) (*24*, *25*), would be effective LRAs. We report the design and synthesis of a library of 15 EBC-46 analogs including a prodrug variant, positive (bryostatin 1) and negative (blocked pharmacophore) controls, and the comparison of their PKC-isoform affinities and selectivities, their ability to activate the nuclear factor κB (NF-κB) pathway, and their efficacy in reversing HIV latency in Jurkat-Latency (J-Lat) cells. When compared to bryostatin 1, a potent PKC modulator and leading LRA, select analogs exhibit superior PKC affinities (some remarkably at a picomolar level), unique isoform selectivities (some like EBC-46 and one unprecedented), and notably better functional activity (LRA) in a J-Lat latency reversal model system.

## RESULTS

### PKC and the C1 domain

PKC is a family of kinases that regulate fundamental biological processes such as cell growth, apoptosis, transcription, and immune functions as well as more complex organismal functions including learning and memory (*26*). The PKC family of isozymes is divided into three groups: eight highly homologous isozymes classified as either conventional (α, βI, βII, and γ) or novel (δ, ε, η, and θ) and more distantly related isozymes classified as atypical (ζ and ι). Although structurally similar, different PKC isozymes have been shown to control distinct downstream cellular pathways (*27*), presenting opportunities for isoform-selective PKC modulators to address specific diseases (*28*). Conventional and novel isozymes of PKC are activated by the binding of small molecule ligands (e.g., diacylglycerol, bryostatin 1, and EBC-46) to their C1 domains, which results in cytosolic PKCs being translocated to cellular membranes where they phosphorylate various client proteins (*26*, *29*). Because the C1 domain binding pocket features a polar interior with a nonpolar rim (*29*, *30*), it follows that ligands binding to the C1 domain, including LRAs, have a matched polar PKC binding domain with H-bond donors and acceptors that associate with complementary functionality in the polar binding pocket and a lipophilic domain that serves to cap the binding pocket and to accommodate insertion of the enzyme-ligand complex into a membrane (Fig. 2B) (*30*).

### Design and synthesis of EBC-46 analogs

Our seminal computer modeling studies (*24*) on structurally diverse PKC modulators showed that they all have a similar spatial array of H-bond donors and acceptors and lipids, constituting a common binding pharmacophore. In tiglianes, like the phorbol esters, this pharmacophore corresponds to functional groups at C20, C3/C4, and C9 as well as lipids at C12 and C13 (Fig. 2A). This model, based initially on terpenoid and alkaloid modulators, was later found to be applicable to newly discovered PKC modulators such as the polyketide bryostatin 1 and its analogs (*25*, *31*) and was further validated by the first x-ray crystal structure of phorbol C13-acetate bound to PKC-δ (*32*). This pharmacophore model has been further corroborated by more recent and impressive structure-activity studies and x-ray crystal structures of various C1 domain-ligand complexes (Fig. 2C) (*16*, *23*, *30*, *32*–*37*). In our current studies, we found that the functional groups of EBC-46 fit this pharmacophore model and include additional functionality (C5-hydroxyl, C6/C7 epoxide, and branched C12/C13 lipids) that could account for its unique biological activity and clinical utility and, thus, for the potential of its analogs to affect HIV latency reversal. Prompted by our ongoing research and therapeutic interest in PKC modulators, we previously reported a sustainable gram-scale synthesis of EBC-46 from readily available phorbol (**1**; Fig. 1A) (*22*). As described herein, using this unique and scalable synthetic access to analogs and guided by our pharmacophore model for the binding of phorbol-type ligands to PKC, we have identified and systematically modified potential pharmacophoric sites in EBC-46 precursors and derivatives and explored their activity as LRAs (Figs. 1B and 2A) (*24*). Several intermediates in our synthetic route from phorbol also served as diversification nodes that, being more step-economically accessible than EBC-46 itself, provide unique and facile access to numerous highly functionalized nonnatural tiglianes (*38*, *39*), enabling investigation of their PKC affinities and selectivities and role as LRAs and, more generally, as leads directed at antigen-enhanced immunotherapies (*40*) and at other PKC-related clinical indications (*22*, *41*–*43*).

To better understand how our phorbol ester pharmacophoric model for PKC extends to EBC-46 and, thus, how it could be used to design improved modulators and LRAs, we simulated the docking of EBC-46 to the C1b domain of PKC-δ (Fig. 2C). This in silico model (see the Supplementary Materials for the computational modeling method) suggests that the C5-hydroxyl group plays an added pharmacophoric role as it engages in a second hydrogen bond to the Leu251 backbone amide in combination with the C20-hydroxyl group. On the basis of our pharmacophoric model (*24*) and studies on the role of the lipophilic region (Fig. 2A) (*30*, *34*, *35*, *44*) and C1 domain ligands in PKC binding (*16*, *23*, *36*, *45*), we also examined the often-underestimated role of lipid variations in PKC binding and isoform selectivity. We then tested the functional activities of these systematically modified analogs in an assay for NF-κB activation (*23*), a pathway involved in HIV latency reversal (*46*). Last, we then evaluated our analogs in a J-Lat latency reversal assay. Notably, many showed superior activity when compared to bryostatin 1, one of the more promising previously reported LRAs. This function-oriented synthesis strategy (*41*–*43*) based on synthesis- and computer-informed design provided a systematic analysis of A-, B-, and C-ring variations of EBC-46 and has resulted in promising PKC modulators and potent leads for HIV latency reversal.

**Fig. 3.**
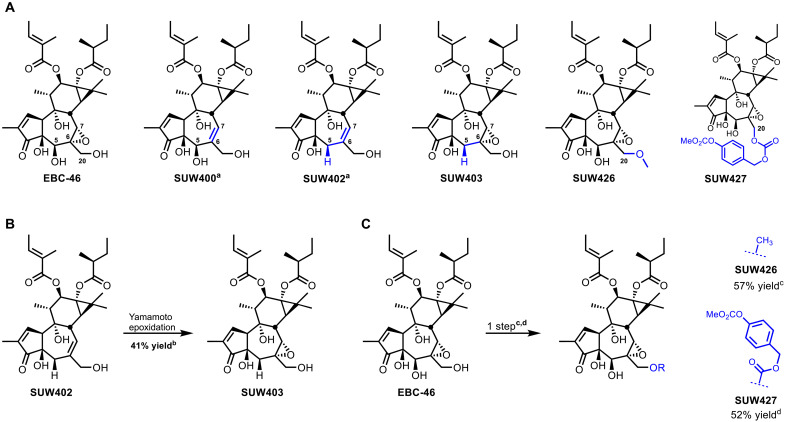
Reactions and conditions for the synthesis of B-ring analogs of EBC-46. (**A**) Overview of B-ring analogs of EBC-46. (a) Reactions and conditions for synthesis of SUW400 and SUW402 previously reported (*22*). (**B**) Reactions and conditions for the synthesis of SUW403. (b) Vanadyl isopropoxide (0.05 equiv), Yamamoto ligand (0.1 equiv), cumene hydroperoxide (2 equiv), toluene, 0°C to room temperature. (**C**) Reactions and conditions for the synthesis of SUW426 and SUW427. (c) For SUW426: methyl triflate (6 equiv), 2,6-di-*tert*-butyl pyridine (10 equiv), CH_2_Cl_2_. (d) For SUW427: 4-methylcarbonate benzyl chloroformate (1 equiv), triethylamine (NEt_3_) (1 equiv), 4-dimethylaminopyridine (DMAP) (1 equiv), CH_2_Cl_2_, 4°C.

Our molecular editing of the EBC-46 B-ring began by exploring the importance of its C5β-hydroxy-C6α,C7α-epoxy functionality that is also found in several other phorbol-derived natural products that exhibit potent anticancer or anti-HIV activity such as yuanhuacine and gnidimacrin (*47*–*49*). Toward this end, we prepared a systematically varied series of B-ring analogs (Fig. 3A). SUW400 features a C6,C7 alkene in place of the C6,C7-α epoxide of EBC-46. SUW403 lacks oxidation at C5 but includes the C6,C7-α epoxide. SUW402 is simply a phorbol derivative that lacks oxidation at C5 but includes a C6,C7 alkene and the EBC-46 ester groups. The syntheses of SUW400 and SUW402 have already been reported (*22*). We prepared SUW403 in one step from SUW402 by Yamamoto epoxidation (Fig. 3B) (*50*). We prepared a C20-methyl ether (SUW426), lacking the pharmacophoric hydroxyl group at C20, as a negative control tool compound to probe for activity potentially arising from a PKC-independent pathway (Fig. 3C) (*51*–*53*). On the basis of our pharmacophoric model and the computational modeling of the C1 domain binding site, we expected that the removal of the C20 alcohol’s ability to function as a hydrogen bond donor and the steric bulk of the methyl group would make SUW426 less effective in binding to the C1 domain of PKC. We prepared SUW426 directly from EBC-46 by treatment with excess methyl triflate and 2,6-di-*tert*-butyl pyridine as a hindered base. We also prepared a C20-esterase–releasable prodrug (SUW427), a first in this class, as we have found, in related studies, that such modifications beneficially influenced biodistribution and successfully expanded the therapeutic window of bryostatin 1 by more than 100-fold (*17*). We prepared this prodrug variant of EBC-46 (SUW427) directly from EBC-46 and the corresponding benzyl chloroformate (Fig. 3C).

**Fig. 4.**
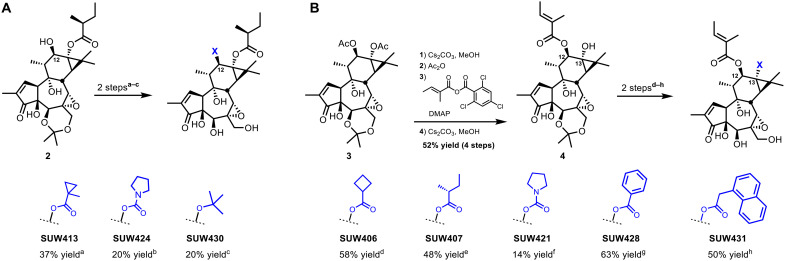
Reactions and conditions for the synthesis of C-ring analogs of EBC-46. (**A**) C12 analog synthesis. (a) For SUW413: 1-methylcyclopropanecarboxylic acid (2.2 equiv), NEt_3_ (4 equiv), 2,4,6-trichlorobenzoyl chloride (2 equiv), DMAP (2.6 equiv), toluene, and 60°C; then *p*-toluenesulfonic acid (TsOH) [0.21 M in H_2_O:acetonitrile (MeCN)]. (b) For SUW424: *N*-methylimidazole (36 equiv), CDI (7 equiv), MeCN; then pyrrolidine; then TsOH (0.21 M in H_2_O:MeCN). (c) For SUW430: lutidinium bistriflimide (0.5 equiv), *tert*-butyl 2,2,2-trichloro acetimidate (11.6 equiv), fluorobenzene, 40 to 50°C; then pyridinium *p*-toluenesulfonate (PPTS) (12 equiv), MeOH:MeCN (5:1, v/v), 60°C. (**B**) Reactions and conditions for the synthesis of C13 C-ring analogs of EBC-46. (d) For SUW406: cyclobutanecarboxylic acid (1.8 equiv), 1-ethyl-3-(3-dimethylaminopropyl) carbodiimide (EDC) (2 equiv), NEt_3_ (3 equiv), DMAP (0.25 equiv), CH_2_Cl_2_; then TsOH (0.21 M in H_2_O:MeCN). (e) For SUW407: (*R*)-2-methylbutanoic acid (1.8 equiv), EDC (2 equiv), NEt_3_ (3 equiv), DMAP (0.25 equiv), CH_2_Cl_2_; then TsOH (0.21 M in H_2_O:MeCN). (f) For SUW421: *N*-methylimidazole (32 equiv), CDI (3 equiv), MeCN; then pyrrolidine; then TsOH (0.21 M in H_2_O:MeCN). (g) For SUW428: benzoic acid (3.5 equiv), NEt_3_ (3.5 equiv), DMAP (3.9 equiv), CH_2_Cl_2_; then PPTS (12 equiv), MeOH:MeCN (5:1, v/v), 60°C. (h) For SUW431: 1-napthaleneacetic acid (2 equiv), EDC (2 equiv), NEt_3_ (2.1 equiv), DMAP (0.5 equiv), CH_2_Cl_2_; then PPTS (12 equiv), MeOH:MeCN (5:1, v/v), 60°C.

We directed our molecular editing of the C-ring at variations of the C12 and C13 substituents (Fig. 4). Since the discovery of phorbol myristate acetate and other phorbol esters, it has been found that changes in the C12/C13 lipid domain profoundly influence biological activity and potency (*54*): Phorbol myristate acetate is a potent tumor promoter, while prostratin (C12-deoxyphorbol-C13-acetate) is not. However, prostratin is an LRA lead and the corresponding prostratin-C13-naphthyl acetate differing from prostratin by only a naphthyl group is >130-fold more potent than prostratin as an LRA and is used by clinicians and investigators to estimate HIV reservoirs ex vivo in children and acutely HIV-infected adults (*16*, *37*, *55*, *56*). Similarly, prior work investigating C12 and C13 ester derivatives of EBC-46 revealed that small changes in the lipophilicity of these substituents had important effects on the isoform-selective activation of PKC and on antimelanoma and wound-healing activities (*23*, *57*). We readily synthesized our C12 analogs from **2**, which we made using our previously reported synthetic route (Fig. 4A) (*22*). Because of the potential of the tiglate ester to act as a Michael acceptor or to isomerize, we sought a more metabolically stable C12 isostere featuring a C12 cyclopropane in place of the tiglate-alkene. Toward this end, we prepared SUW413 in two steps: using the mixed Yamaguchi anhydride, generated in situ from the corresponding carboxylic acid and 2,2,2-trichlorobenzoyl chloride, to form the C12 ester followed by subsequent acid deprotection of the C5,C20 acetonide. Because EBC-46 lacking a C12 ester has been identified as a major metabolite (*58*), we envisioned that analogs that featured more esterase-resistant groups at this position would have improved activity. Toward this end, we first targeted the C12-pyrrolidine carbamate analog (SUW424) as it mimics the planar geometry of the tiglate ester but is less hydrolytically labile. In one step, we treated **2** with carbonyl diimidazole (CDI) to form the C12-imidazole carbamate, and the C12-pyrrolidine carbamate was formed rapidly by addition of excess pyrrolidine. Subsequent acid deprotection afforded SUW424. We considered the C12-*tert*-butyl ether SUW430, incorporating another metabolically stable functionality, as yet unexplored in decades of research on tiglianes, as it would also serve as a hydrophobic cap covering the C12 oxygen and thereby mimicking prostratin, which lacks a C12 oxygen. This tactic would open step-economical access to “pseudodeoxy” analogs, in which the oxygen is buried in a hydrocarbon environment and would thus avoid the multistep deoxygenation required in our scalable synthesis of prostratin and its analogs from phorbol (*37*, *59*). This branched ether proved to be one of the first examples of an active phorbol derivative with a non-ester lipid at C12 (vide infra) (*60*). We prepared SUW430 in two steps from **2** by first using highly effective noncoordinating acid-base–catalyzed *tert*-butyl etherification (*61*) followed by a mild acid–mediated deprotection of the C5,C20 acetonide.

The corresponding synthesis of C13 analogs began from **3**, which we made using our previously reported synthetic route (Fig. 4B) (*22*). We cleaved the C12 and C13 acetate groups to the corresponding diol using cesium methoxide. Chemoselective reinstallation of the C13 acetate using acetic anhydride (with no acylation catalyst) followed by C12-tiglate esterification and subsequent C13-acetate deprotection yielded the C13-hydroxy-C12-tiglate ester **4**. To probe the importance of the chiral ester at C13, we sought to make an achiral cyclobutyl ester analog (SUW406) and a diastereomeric (*R*)-methyl-butyrate ester analog of EBC-46 (SUW407). SUW406 has an achiral near-planar array of carbons analogous to the methylbutyrate ester in EBC-46. SUW407 probes whether the C13 chirality in EBC-46 is important as our pharmacophore model suggests that it would be in contact with the membrane and not the chiral PKC binding domain. We made both compounds in two steps from **4** using standard Steglich esterification conditions followed by subsequent acid deprotection of the C5,C20 acetonide. The C13 ester carbonyl of phorbol esters and prostratin has been observed to participate in a transannular hydrogen bond with the C9 alcohol (*30*, *32*). This hydrogen bond plays a role in preorganizing the C13 substituent. Disruption of this intramolecular hydrogen bond in prostratin and 12-deoxyphorbol-13-phenylacetate, by converting the C13 esters to the corresponding C13 ethers, results in compounds with weak PKC binding affinity and poor in vitro activity (*62*). To investigate whether the binding affinity of these compounds could be improved by strengthening this hydrogen bond, we increased the Lewis basicity of the carbonyl by incorporating it into a C13 carbamate. We prepared SUW421 using the previously described carbamoylation/deprotection conditions for the preparation of SUW424. Similarly, we prepared a C13-benzoate ester, as a more nonpolar isostere of SUW421, from **4** using benzoic anhydride. Subsequent acid deprotection of the C5,C20 acetonide afforded SUW428. In our previous study of prostratin esters and their ability to activate latent HIV reservoirs in vitro and ex vivo, we found that the best performing compound featured a (1-napthyl)acetate ester at C13 (*16*). This compound was >100-fold better at activating HIV reservoirs than prostratin itself and is now used to measure HIV reservoirs ex vivo in children living with HIV and acutely infected adults (*55*, *56*). Inspired by this finding, we installed a C13-(1-naphthyl)acetate ester in **4** using standard Steglich conditions. Subsequent acid deprotection of the C5,C20 acetonide afforded SUW431.

**Fig. 5.**
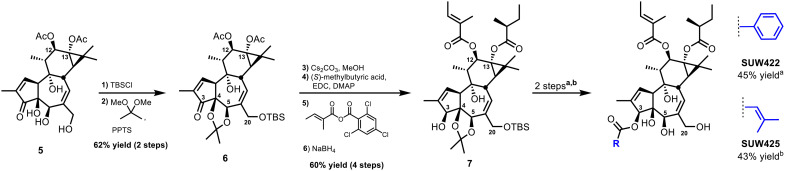
Reactions and conditions for the synthesis of A-ring analogs of EBC-46. (a) For SUW422: benzoyl chloride (10 equiv), DMAP (0.5 equiv), pyridine, 50°C; then *p*-toluenesulfonic acid (TsOH) (0.105 M in H_2_O:MeCN), 80°C. (b) For SUW425: senecioic acid (20 equiv), EDC (20.5 equiv), NEt_3_ (23 equiv), DMAP (0.5 equiv), CH_2_Cl_2_, 40°C; then TsOH (0.105 M in H_2_O:MeCN), 80°C.

We began our molecular editing of the A-ring by exploring ester variants of the C3 alcohol (Fig. 5)—a functional group found in structurally homologous PKC-modulating compounds with potent biological activity. The synthesis of C3 analogs began from **5**, which we made using our previously reported synthetic route (Fig. 5) (*22*). We prepared intermediate **6** from **5** by first protecting the C20 and C4,C5 alcohols with a *t*-butyldimethylsilyl group and an acetonide, respectively. Subsequent deacetylation of the C12,C13-acetate esters, selective esterification of the C12,C13 alcohols, and stereoselective reduction of the C3 ketone with sodium borohydride afforded the readily derivatizable C3-hydroxy intermediate **7**. Because the C6,C7 epoxide was not compatible with the protection and deprotection conditions described above, the A-ring analogs also have a C6,C7 alkene in the B-ring. Given that gnidimacrin is highly effective at activating latent HIV reservoirs and features a C3-β-benzoate ester (*48*), we installed a C3-benzoate ester to mimic the structure of its A-ring by treating **7** with benzoyl chloride in pyridine. Subsequent acid deprotection of the B-ring protecting groups afforded the final compound SUW422. In a study looking at the HIV latency reversal activity of various ingenol esters, the C3-senecioate ester of ingenol was identified as the most efficacious compound with the ability to activate latent reservoirs to the same degree as their anti-CD3/CD28 T cell positive control (*45*). Thus, we prepared a C3-senecioate ester by Steglich esterification of **7**, and subsequent acid deprotection of the B-ring protecting groups afforded SUW425.

### Preclinical evaluation: Cell-free PKC binding assay

**Fig. 6.**
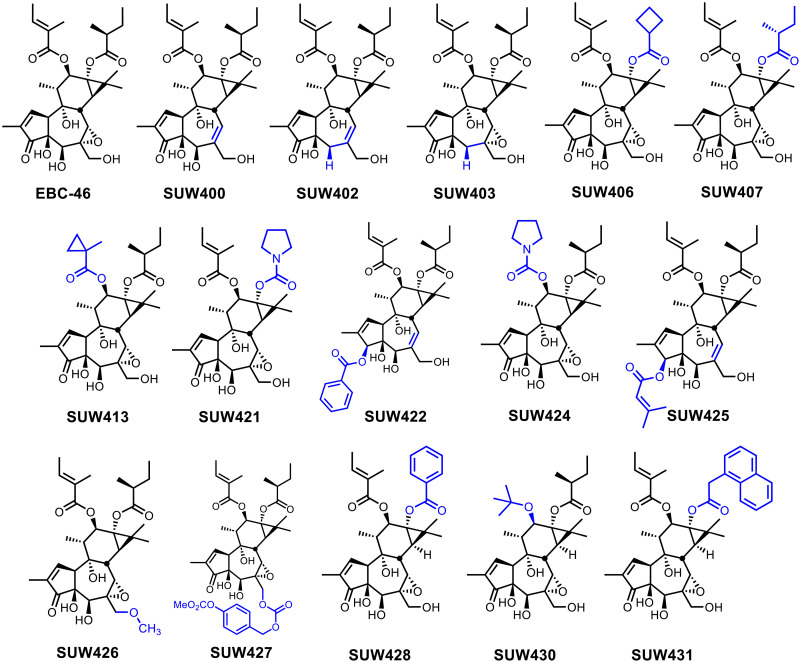
A-, B-, and C-ring analogs of EBC-46 prepared from phorbol. Functional group modifications highlighted in blue.

**Fig. 7.**
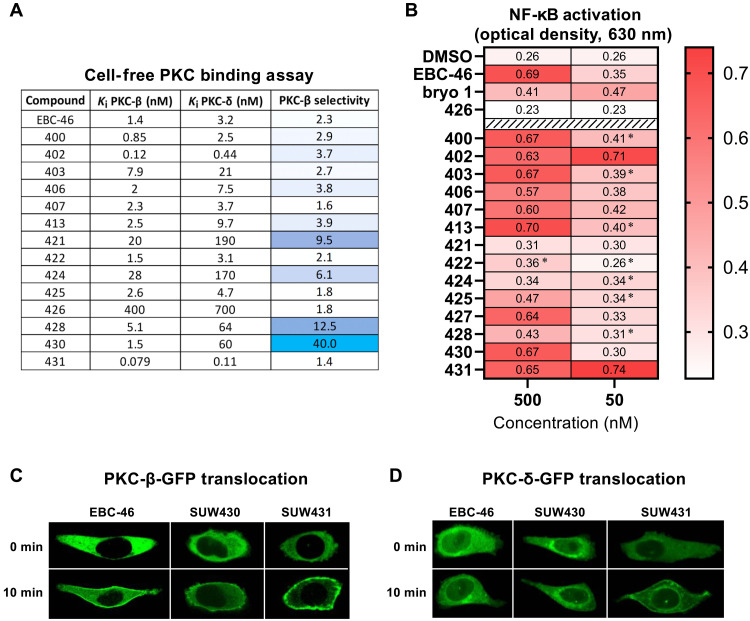
Evaluation of EBC-46 analogs as PKC modulators. (**A**) Cell-free PKC competition binding assay data for PKC-β-I and PKC-δ against radiolabeled PDBu (95% confidence interval range indicated in parentheses). *K*_i_, inhibition constant. (**B**) NF-κB activation assay using A549 cells with an alkaline phosphatase reporter. Data collected 24 hours after dosing; all samples assessed in quadruplicate and standard errors <5%, unless noted [asterisk (*), standard error between 5 and 10%]. (**C** and **D**) Confocal microscopy images of CHO-K1 cells transfected with PKC-GFP and treated with PKC modulators (EBC-46, SUW430, and SUW431). Translocation assessed at 10 min after compound addition.

**Fig. 8.**
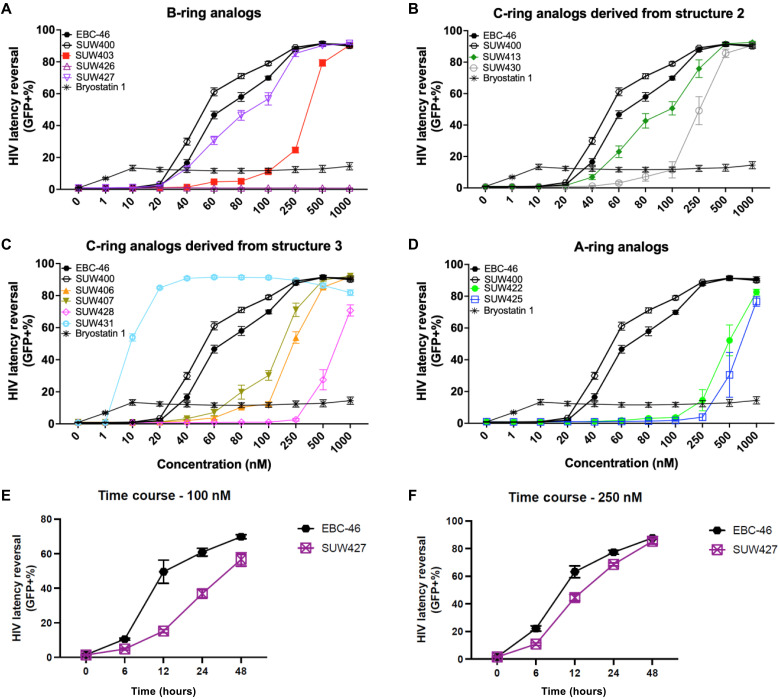
Evaluation of EBC-46 analogs as LRAs. J-Lat clone 10.6 cells were treated with ascending concentrations of tigilanol tiglate analogs for 48 hours. Bryostatin 1, a natural PKC modulator, served as a positive control. All conditions were completed in three independent biological replicates, each in technical duplicates, resulting in *n* = 6. The same bryostatin 1, EBC-46, and SUW400 curves are shown in (A) to (D) for comparison purposes. Dose curves are separated by ring modifications as follows: (**A**) B-ring analogs (Fig. 3), (**B**) C-ring analogs derived from **2** (Fig. 4A), (**C**) C-ring analogs derived from **3** (Fig. 4B), and (**D**) A-ring analogs (Fig. 5). (**E**) Time course experiment showing EBC-46 and SUW427 (100 nM) over the course of 48 hours and (**F**) time course experiment showing EBC-46 and SUW427 (250 nM) over the course of 48 hours.

With a diverse library of compounds bearing unique functionalities on the A-, B-, and C-rings (Fig. 6), we began to explore how these modifications affect PKC binding (Fig. 7A), NF-κB activation (Fig. 7B), cell permeation and PKC translocation (Fig. 7, C and D), and latency reversal in J-Lat cells (Fig. 8). Because binding to the C1 domain of PKC is a prerequisite to PKC pathway involvement, our evaluation of the biological activity of these compounds began with a cell-free competitive binding assay against tritiated phorbol-12,13-dibutyrate (^3^H-PDBu) using representative conventional (PKC-β-I) and novel (PKC-δ) isoforms of PKC (*63*). We conducted this assay with phosphatidyl serine vesicles in solution that mimic the cellular membrane and play a critical role in the binding of small molecules to the PKC C1 domain (Fig. 7A) (*64*).

EBC-46 showed potent binding to the two isoforms of PKC (1.4 nM for PKC-β and 3.2 nM for PKC-δ) with modest twofold selectivity for PKC-β over PKC-δ (Fig. 7A). SUW400, which features a C6,C7 alkene in place of the C6,C7-α epoxide and is a readily accessible analog, had nearly identical binding affinity and selectivity compared to EBC-46, suggesting that the B-ring epoxide is not critical to PKC binding as predicted by our in silico modeling (Fig. 2C) and suggesting that this more accessible compound might be clinically useful. SUW403, the des-C5-hydroxy analog of EBC-46, showed an approximately fivefold decrease in potency while retaining similar isoform selectivity for PKC-β over PKC-δ, suggesting that C5-OH plays a role in PKC binding, in line with our modeling studies. SUW402, a phorbol diester featuring the esters found on EBC-46, showed highly potent binding to both tested isoforms of PKC (0.12 nM for PKC-β and 0.44 nM for PKC-δ). SUW426 has a C20 methoxy group in place of C20-OH, a hydrogen bond donor and pharmacophoric group that inserts deeply into the narrow C1 domain binding pocket. As anticipated from our modeling studies, the added steric bulk of SUW426, in combination with its inability to serve as a C20 hydrogen bond donor (Fig. 2, B and C), resulted in an approximately 250-fold decrease in PKC binding affinity, thus serving as a tool compound to explore non-PKC effects. SUW422, featuring a hydrolytically stable C3-β-benzoate ester akin to gnidimacrin, had nearly identical binding potency and selectivity to EBC-46, in line with our pharmacophore model. Similarly, the C3-senecioate ester analog (SUW425) had binding affinity and selectivity that was approximately the same as EBC-46. While C3 esters are typically found in biologically active ingenanes and daphnanes, our modeling and data indicate that it also applies to our analogs. Modifications to the C12 and C13 positions of the C-ring, the region that, on the basis of our modeling and subsequent x-ray analyses, is putatively embedded in the cell membrane during PKC activation, proved to be crucial for potent and selective binding to PKC (Fig. 7A). In line with our modeling, SUW407, which features the diastereomeric C13-(*R*)-methylbutyrate ester, showed similar binding affinities for both PKC-β and PKC-δ. This finding suggests that the chirality of this small ester is inconsequential to extracellular PKC binding and selectivity as expected from its membrane microenvironment. SUW406, featuring a pseudoplanar achiral C13-cyclobutanoate ester akin to the EBC-46 chiral butanoate, showed a similar binding affinity to PKC-β compared to EBC-46 with moderate fourfold selectivity over PKC-δ. This suggests that the relatively inexpensive pseudoplanar five-carbon cyclobutanoic acid ester might be a useful surrogate for the chiral (*S*)-methylbutyric acid ester of EBC-46. SUW413, which features an achiral α-cyclopropyl ester, showed very similar binding affinity to both PKC-β and PKC-δ compared to SUW406. This finding suggests that the α-cyclopropyl ester might serve as a more stable substitute for the α,β-unsaturated tiglate ester found in EBC-46. SUW431, featuring C13-(1-naphthyl)acetate ester), had exceptionally increased PKC binding potency with ~20-fold stronger binding for PKC-β (79 pM) and 30-fold stronger binding for PKC-δ (110 pM) when compared to EBC-46. SUW431 has approximately 10-fold greater affinity to PKC-δ than that reported for the exemplary analog from the prostratin series (SUW013) (*55*, *56*). SUW424 and SUW421, featuring pyrrolidine carbamates at the C12 and C13 positions, respectively, showed relatively weak binding to PKC-β (~25 nM) but increased selectivity over PKC-δ (6- and 10-fold, respectively). We hypothesized that this >10-fold decrease in potency is due to the increased polarity of the carbamate functional group, relative to the esters found on EBC-46, making the lipophilic region less effective in embedding in a membrane. Therefore, we made SUW428 as a less polar isostere of the pyrrolidine carbamate. In support of our hypothesis, this compound retained >12-fold selectivity for PKC-β over PKC-δ while restoring the potency to single-digit nanomolar levels for PKC-β (5.1 nM). Considering that SUW431, featuring an aryl lipid separated from its C13 carbonyl by a methylene spacer, was unselective (1.4-fold for PKC-β), this finding suggests that the conformationally restricted arrangement of the aryl group of SUW428 might be responsible for the observed PKC-β–selective mode of binding. Unexpectedly, SUW430, featuring a C12 *tert*-butyl ether, showed nearly identical binding affinity to PKC-β (1.5 nM) as EBC-46 with 40-fold selectivity over PKC-δ (60 nM). This unprecedentedly selective compound, incorporating a non-ester lipid at C12, was the most PKC-β–selective binder in this series and opens a new approach to achieve isoform selectivity as would be desired for various therapeutic indications (*23*, *28*, *48*, *65*–*67*).

### PKC-GFP translocation assay

Because the extracellular binding of ligands to the C1 domain of PKC is necessary but not sufficient for their cellular permeation and intracellular PKC activation, we tested EBC-46, our most potent analog (SUW431), and our most isoform-selective analog (SUW430) in a translocation assay using PKC-β and PKC-δ proteins fused to green fluorescent protein (GFP). Using confocal microscopy, this assay allows us to visualize, in real time, cell permeation and the ligand-induced translocation of specific PKC isoforms from the cytosol to the membranes of the cell—the hallmark of PKC activation (*29*, *68*). All three of these compounds were able to quickly enter cells and effectively translocate PKC-β at 500 nM and PKC-δ at 1500 nM in CHO-K1 cells within 10 min, confirming that these compounds can permeate cellular membranes as well as access and activate both PKC isoforms (Fig. 7, C and D). In alignment with past studies on the activation of PKC in CHO-K1 cells (*22*, *65*, *69*), we observed strong plasma membrane translocation of PKC-β (Fig. 7C). SUW431 induced strong perinuclear and plasma membrane translocation of PKC-δ, while EBC-46 and SUW430 selectively induced perinuclear translocation of PKC-δ (Fig. 7D). This nuclear membrane–biased translocation profile of PKC-δ in CHO-K1 cells resembles that of 12-deoxyphorbol-13-phenylacetate—a prostratin derivative that exhibits potent HIV latency reversal in vitro and ex vivo (*37*, *65*, *69*).

### NF-κB activation assay

As a high-throughput method of screening compounds for their ability to get into cells and activate immunological pathways related to HIV latency reversal (*46*, *70*), we conducted an NF-κB reporter assay using A549 cells stably integrated with a secreted embryonic alkaline phosphatase reporter under the control of a promoter fused to five NF-κB binding sites (Fig. 7B) (*48*, *70*, *71*). We assessed NF-κB activation 24 hours after compound addition, and we observed that the performance of our analogs in this assay was strongly correlated with their performance in the cell-free binding assay (Fig. 7, A and B). At 50 nM, EBC-46 and most of our analogs displayed minimal activation of NF-κB relative to the dimethyl sulfoxide (DMSO) control. However, at 50 nM, the highly potent C13-(1-napthyl)acetate ester of EBC-46 (SUW431) and the phorbol diester (SUW402) induced approximately three times the basal level of NF-κB activation indicated by our DMSO negative control. However, in line with the ability of potent PKC activators to induce feedback inhibition mechanisms (*26*), we observed that SUW431 and SUW402 also exhibited strong activation of NF-κB at higher concentrations but with an inverse dose response. At 500 nM, EBC-46 induced ~2.5 times the basal level of NF-κB activation. SUW426, the C20-methyl-ether analog of EBC-46, did not exhibit NF-κB activation at either concentration tested in this assay, consistent with its design as a negative control. As expected, the less potent C12,C13-carbamate analogs (SUW424 and SUW421) had minimal activity in this assay at 50 and 500 nM. Compared to EBC-46, both A-ring analogs (SUW422 and SUW425) had similar binding affinities for PKC-β and PKC-δ, yet they exhibited minimal NF-κB activation at both 50 and 500 nM. The lack of functional activity from SUW422 and SUW425 in the NF-κB assay could be a result of poor cell membrane permeability or poor hydrolytic stability of these compounds under the assay conditions, suggesting that cell-free PKC binding affinity is necessary but not sufficient for PKC activation. The desoxy-B-ring analogs (SUW400 and SUW403) and the C-ring analogs with similar esters to EBC-46 (SUW406, SUW407, and SUW413) all demonstrated comparable activity to EBC-46 at 50 and 500 nM. The prodrug variant of EBC-46 (SUW427) also showed similar activity to EBC-46 at both concentrations. This is substantial because we expect this compound to have different biophysical properties than EBC-46 and potentially a larger therapeutic window for systemic distribution as was observed with the prodrug of bryostatin 1 (*17*). SUW428, one of the more PKC-β–selective analogs, was less effective at inducing NF-κB activation than EBC-46 at 500 nM, which is in line with it having approximately three times lower affinity for PKC-β compared to EBC-46. Although SUW430 has the same binding affinity as SUW428 for PKC-δ (~20× weaker than EBC-46), it has the same affinity as EBC-46 for PKC-β, and it performed comparably to EBC-46 at both concentrations in the NF-κB assay.

Because SUW402 and SUW431 are potent activators of NF-κB, we used an enzyme-linked immunosorbent assay to measure their ability to up-regulate inflammatory cytokine production (tumor necrosis factor–α and interleukin-8). We stimulated A549 cells with SUW402 and SUW431 at the same concentrations used in the NF-κB activation assay: 500 and 50 nM (figs. S1 and S2). SUW402 did not produce detectable levels of tumor necrosis factor–α at either concentration, while SUW431 produced minimal amounts (8.4 pg/ml) at 500 nM, an amount close to the assay’s lowest detectable limit of 3.5 pg/ml. At 500 or 50 nM, both SUW402 and SUW431 produced only modest levels of interleukin-8, a 15 to 25% increase relative to the DMSO negative control. These enzyme-linked immunosorbent assay results suggest that potent up-regulation of NF-κB corresponds with minimal up-regulation of inflammatory cytokine expression in this cell line.

### J-Lat HIV latency reversal assay

Having shown that our lead compounds bind cell-free PKC isoforms, enter cells, translocate PKC-GFP fusion proteins, and activate NF-κB transcription factors, we next set out to determine whether our analogs were capable of reversing HIV from latency. We performed in vitro assays using T lymphocyte J-Lat clone 10.6, which contains a latent near full-length replication-incompetent integrated HIV DNA genome (*72*). J-Lat 10.6 lacks two viral genes, *env* and *nef*, which encode for the envelope protein and an accessory protein, respectively, and thus cannot initiate a spreading HIV infection. The integrated HIV provirus also encodes a GFP reporter gene, allowing latency reversal to be measured by quantification of GFP expression (fig. S1). Bryostatin 1 is a naturally occurring PKC modulator that is commonly used in HIV latency reversal studies because of its effectiveness and hence served as our positive control (*73*). In this latency reversal assay, we quantified GFP expression 48 hours after compound addition and observed bryostatin 1 to be maximally effective at concentrations of 40 nM or higher with latency reversal in 12% of cells (Fig. 8). Given the efficacy of bryostatin 1 as an LRA (*73*, *74*), EBC-46 and many of its analogs tested in this J-Lat assay induced latency reversal in >90% of cells. SUW400, a structurally simplified analog of EBC-46, performed similarly, inducing maximal latency reversal of >90% of cells at a concentration of 250 nM, supporting the notion that the C6,C7-α epoxide of EBC-46 does not play an essential role in its membrane permeability or functional activity related to HIV latency reversal. SUW431, featuring the C13-napthylacetate, was the most effective HIV latency reversal agent, inducing latency reversal in 90% of cells at a concentration of just 40 nM (Fig. 8C). All other analogs with B-ring (SUW403), C-ring (SUW406, SUW407, SUW413, SUW428, and SUW430), and A-ring modifications (SUW422 and SUW425) strongly induced HIV latency reversal (in 80 to 90% of cells) but required a higher dose than EBC-46 before we detected reversal activity (Fig. 8). SUW427, an esterase-releasable prodrug of EBC-46, also performed similarly to EBC-46 with a maximum efficacy of latency reversal in >90% of cells at a dose of 250 nM. Notably, in a time course study of this prodrug’s activity, we observed that it demonstrated a time-dependent response as expected for a prodrug. At a concentration of 100 nM, prodrug SUW427 had a clear delayed HIV latency reversal in J-Lat 10.6 cells, achieving comparable HIV latency reversal ability to EBC-46 at the same concentration (Fig. 8E). At a concentration of 250 nM, SUW427 almost converged with its parent compound at all time points and reached maximal latency reversal (Fig. 8F). The gradual release of EBC-46 from its prodrug form provides an alternative mode of administration that avoids the bolus effect associated with rapid drug administration (*17*). SUW426, the C20-methyl-ether analog of EBC-46, did not produce latency reversal at any tested concentration (Fig. 8A). This is consistent with its lack of activity in the NF-κB activation assay and its design as a negative control as it is structurally similar to EBC-46 but unable to bind to the C1 domain of PKC at the concentrations used in this assay as it lacks a complete pharmacophore.

## DISCUSSION

On the basis of our function-oriented synthesis approach and guided by our pharmacophore model for C1-domain PKC modulators and related SAR studies of their PKC modulation (*16*, *23*–*25*, *30*, *34*, *35*, *45*, *75*), we leveraged our scalable, sustainable, and step-economical synthesis of EBC-46 (11 steps, 17% overall yield from naturally abundant phorbol) to design, prepare, and evaluate 15 analogs of EBC-46 (*22*, *39*). Our synthesis provides unique and scalable access to EBC-46 and its analogs, many of which are not accessible from the rare natural source endemic to a small rainforest region of northeastern Australia. We examined structural variations at pharmacophoric hotspots in the A-, B-, and C-rings, including a prodrug analog (SUW427) and a negative control (SUW426). Several analogs exhibited binding affinities to PKC-β and PKC-δ isoforms that are comparable to bryostatin 1 and EBC-46, and two (SUW402 and SUW431) exhibited remarkable affinities in the picomolar range. Several analogs (SUW421, SUW424, SUW428, and SUW430) showed PKC isoform selectivities substantially different from EBC-46 (2.3× preference for PKC-β) with selectivities ranging from a 6× to 40× preference for binding PKC-β over PKC-δ, representing unprecedented selectivities and progress nearing the long-sought goal of PKC isoform–selective modulation (*65*, *69*, *75*–*77*). These function-oriented synthesis results (*38*, *41*) underscore the fact that nature’s compounds are great leads but are not evolved or optimized for human therapeutic indications (*38*, *41*, *42*, *78*).

Complementing our evaluation of the affinities of our compounds in cell-free PKC isoform binding assays and the ability of our compounds to enter cells and activate PKC in PKC-GFP translocation assays, we next conducted an NF-κB reporter assay in A549 cells as a high-throughput method of screening compounds for functional activity relevant to latency reversal (*70*). We observed that the performance of our analogs in this assay was strongly correlated with their performance in both the cell-free binding assay and in vitro J-Lat latency reversal assay. All the tested compounds (excluding the non-PKC binding negative control compound SUW426) induced HIV expression from latency in J-Lat cells, and, of particular consequence with respect to “kick and kill” approaches, the maximum level of latency reversal achieved with these compounds (>90%) greatly exceeds that induced by bryostatin 1 (12%), a leading LRA taken into a clinical trial but dosed too low to detect activity (*74*). Although some analogs have remarkable isoform-selective affinity to PKC-β, these highly selective activators showed less potent activity in the in vitro NF-κB reporter assay and the J-Lat latency reversal assay, suggesting that activation of PKC-δ is more important than activation of PKC-β for HIV latency reversal, an observation in line with previous work (*79*). Notably, SUW431 represents an ultrapotent latency reversal agent that effectively activates latent HIV at substantially lower concentrations than EBC-46, and structurally simplified analogs of EBC-46 (e.g., SUW400) demonstrate the potential for more synthetically accessible derivatives of EBC-46, such as unexplored C-ring derivatives of SUW400, to have superior functional activities.

Notably, SUW133 (a simplified bryostatin 1 analog with a wider therapeutic window than its parent compound) (*80*) was capable of delaying rebound when administered alone to HIV-infected, ART-treated humanized mice (*81*) and was even more effective when natural killer cells were also introduced as a “kill” arm, resulting in 40% of the animals not rebounding in vivo after ART discontinuation for the duration of the study (*8*). Given this recent success and the strong in vitro efficacy of EBC-46 and its highly accessible analogs identified in the current study, these molecules are potentially superior lead compounds as LRAs for “kick and kill” approaches to HIV eradication. Although EBC-46 is Food and Drug Administration approved for treating cancers using a direct injection into solid tumors (*82*), the compatibility of EBC-46 and its analogs in a systemic drug administration approach has not yet been explored but is warranted by these studies. Future in vivo studies will be necessary to investigate the utility of EBC-46 and its analogs as effective LRAs for the “kick and kill” approach toward HIV eradication. This study shows that readily accessible EBC-46 analogs are notably more effective as LRAs in vitro than bryostatin 1, a lead LRA, strongly warranting their further in vivo study in “kick and kill” approaches to HIV eradication.

## MATERIALS AND METHODS

All reactions were carried out in glassware under an ambient atmosphere, unless otherwise noted. Reactions were concentrated under reduced pressure using a rotary evaporator, unless otherwise noted. Commercial reagents were used as received. Dichloromethane, diethyl ether, dimethylformamide, tetrahydrofuran, and toluene were passed through an alumina drying column (Solv-Tek Inc.) using nitrogen pressure; ethyl acetate and hexanes were obtained from Thermo Fisher Scientific. Analytical thin-layer chromatography was carried out on 250-μm silica gel 60G plates with fluorescent indicator F254 (EMD Millipore). Plates were visualized with ultraviolet light and treated with *p*-anisaldehyde, ceric ammonium molybdate, or potassium permanganate stain with gentle heating. Flash column chromatography was performed using silica gel (230 to 400 mesh, grade 60, particle size of 40 to 63 μm) purchased from Thermo Fisher Scientific. Nuclear magnetic resonance spectra were acquired on a Varian INOVA 600, Varian 400, Bruker 400, or JEOL 500 magnetic resonance spectrometer. ^1^H chemical shifts are reported relative to the residual solvent peak (CDCl_3_, 7.26 ppm; *d*_6_-acetone, 2.05 ppm; CD_3_OD, 3.31 ppm) as follows: chemical shift (δ), multiplicity (app, apparent; b, broad; s, singlet; d, doublet; t, triplet; q, quartet; sex, sextet; h, heptet; m, multiplet; or combinations thereof), coupling constant(s) in Hz, integration. ^13^C chemical shifts are reported relative to the residual solvent peak (CDCl_3_, 77.16 ppm). Infrared spectra were acquired on a Nicolet iS 50 FT-IR Spectrometer (Thermo Fisher Scientific) equipped with an attenuated total reflectance assembly. Optical rotations were acquired on a P-2000 Digital Polarimeter (Jasco). High-resolution mass spectra were acquired at the Vincent Coates Foundation Mass Spectrometry Laboratory at Stanford. Experimental procedures were generally optimized on a small scale, and the results from these optimized procedures are provided below. Reaction procedures were performed by multiple investigators to ensure reproducibility. Characterization data are provided for all isolable compounds. For some steps, the reaction product could be used without chromatographic purification. Because the hazard of new compounds is unknown, all procedures were conducted with full personal protective equipment in a way that avoids exposure. Detailed reaction procedures for each transformation can be found in the Supplementary Materials.
