# Supplementary material for: Synthesis and preclinical evaluation of tigilanol tiglate analogs as latency-reversing agents for the eradication of HIV
Source: Sci Adv. 2025 Jan 24;11(4):eads1911. doi: 10.1126/sciadv.ads1911 (PMC11778240; doi:10.1126/sciadv.ads1911)
Supplement: Supplementary file 1 — Supplementary Text Figs. S1 to S22 [file sciadv.ads1911_sm.pdf]

Science Advances
AAAS

Supplementary Materials for

Synthesis and preclinical evaluation of tigilanol tiglate analogs as latency-reversing agents for the eradication of HIV

Zachary O. Gentry *et al.*

Corresponding author: Matthew D. Marsden, m.marsden@uci.edu; Jerome A. Zack, jzack@ucla.edu; Paul A. Wender, wenderp@stanford.edu

Sci. Adv. **11**, eads1911 (2025)
DOI: 10.1126/sciadv.ads1911

This PDF file includes:

Supplementary Text
Figs. S1 to S22

General Procedures:
All reactions were carried out in glassware under ambient atmosphere unless otherwise noted. Reactions were concentrated under reduced pressure using a rotary evaporator unless otherwise noted. Commercial reagents were used as received or purified using the methods indicated herein. Dichloromethane, diethyl ether, dimethylformamide, tetrahydrofuran, and toluene were passed through an alumina drying column (Solv-Tek Inc.) using nitrogen pressure; ethyl acetate and hexanes were obtained from Fisher Scientific. Analytical thin-layer chromatography (TLC) was carried out on 250 μm silica gel 60G plates with fluorescent indicator F254 (EMD Millipore). Plates were visualized with UV light and treated with *p*-anisaldehyde, ceric ammonium molybdate, or potassium permanganate stain with gentle heating. Flash column chromatography was performed using silica gel (230-400 mesh, grade 60, particle size 40 to 63 μm) purchased from Fischer Scientific. NMR spectra were acquired on a Varian INOVA 600 or Varian 400 or Bruker 400 magnetic resonance spectrometer. ^{1}H chemical shifts are reported relative to the residual solvent peak ($CDCl_3$ = 7.26 ppm, d_6-acetone = 2.05 ppm, CD_3OD = 3.31 ppm) as follows: chemical shift (δ), multiplicity (app = apparent, b = broad, s = singlet, d = doublet, t = triplet, q = quartet, sex = sextet, h = heptet, m = multiplet, or combinations thereof), coupling constant(s) in Hz, integration. ^{13}C chemical shifts are reported relative to the residual solvent peak ($CDCl_3$ = 77.16 ppm). Infrared spectra were acquired on a Nicolet iS 50 FT-IR Spectrometer (ThermoFisher) equipped with an attenuated total reflectance (ATR) assembly. Optical rotations were acquired on a P-2000 Digital Polarimeter (Jasco). High resolution mass spectra (HRMS) were acquired at the Vincent Coates Foundation Mass Spectrometry Laboratory at Stanford.

Experimental procedures were generally optimized on a small scale, and the results from these optimized procedures are provided below. Reaction procedures were performed by multiple investigators to ensure reproducibility. Characterization data is provided for all isolable compounds. For some steps, the reaction product could be used without chromatographic purification. **CAUTION**: Because the hazard of new compounds is unknown, all procedures were conducted with full personal protective equipment in a way that avoids exposure.

SUW403

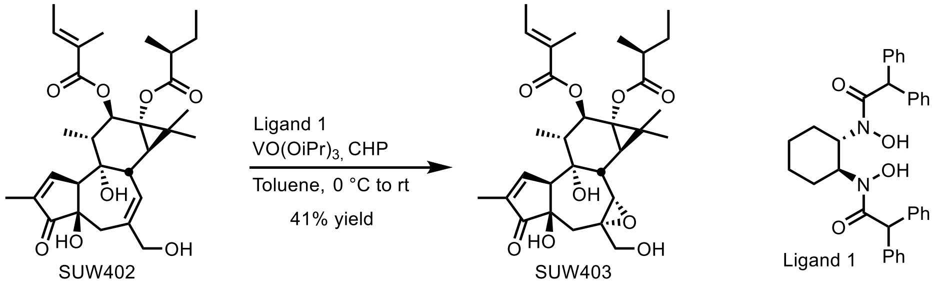

To a flame-dried vial equipped with a stir bar was added vanadyl isopropoxide (0.4 mg, 1.7 µmol, 0.05 equiv) from a stock solution. The reaction mixture was cooled to 0˚C then ligand **1** (1.9 mg, 3.5 µmol, 0.1 equiv) was added from a stock solution. The reaction mixture was stirred at 0˚C for 8 hr. Cumene hydroperoxide (10.6 mg, 69 µmol, 2 equiv) was added directly as a single portion under argon. After stirring for 30 minutes at rt, SUW402 (18.4 mg, 35 µmol, 1 equiv) was added as a solution in toluene (1 mL). The reaction mixture was stirred at rt for 18 hr. TLC analysis indicated complete consumption of SUW402. The crude reaction mixture was filtered through a pad of Celite and rinsed with DCM (3x10 mL). Purification was accomplished by silica gel flash column chromatography (10-40% EtOAc/Hex, 1x7 cm) affording SUW403 (7.7 mg, 41% yield) as a colorless oil. Compound purity was established by TLC (one-spot) analysis.

SUW403 TLC R_f = 0.38 (50% EtOAc/Hex, UV active, purple spot in *p*-anisaldehyde)

^{1}H NMR (400 MHz, $CDCl_3$) δ 7.70 (dd, *J* = 2.6, 1.4 Hz, 1H), 6.86-6.77 (m, 1H), 5.46 (d, *J* = 9.9 Hz, 1H), 4.24 (app s, 1H), 3.71-3.60 (m, 2H), 3.38 (s, 1H), 3.13 (d, *J* = 6.6 Hz, 1H), 2.45-2.35 (m, 1H), 2.22 (s, 1H), 2.04 (s, 2H), 1.97-1.87 (m, 1H), 1.84-1.81 (m, 3H), 1.81-1.76 (m, 6H), 1.76-1.70 (m, 1H), 1.52-1.41 (m, 2H), 1.33 (d, *J* = 6.6 Hz, 1H), 1.27 (s, 3H), 1.25 (s, 3H), 1.14 (d, *J* = 7.1 Hz, 3H), 0.94 (t, *J* = 7.4 Hz, 3H), 0.83 (d, *J* = 6.5 Hz, 3H).

^{13}C NMR (126 MHz, $CDCl_3$, 30 peaks total) δ 208.8, 179.0, 167.6, 163.7, 137.6, 133.2, 128.6, 77.4, 77.0, 73.2, 65.8, 64.9, 64.3, 61.3, 52.0, 45.8, 41.3, 36.5, 36.2, 36.0, 26.7, 26.3, 23.9, 17.4, 16.3, 15.1, 14.6, 12.4, 11.8, 10.1.

HRMS calculated for $C_{30}H_{43}O_9^+$ $[M+H]^+$: 547.2907; found 547.2899.

FTIR (ATR) 3354 (br), 2924, 2854, 1709, 1669, 1462, 1377, 1259, 1211, 1074, 1020, 798 cm^{-1}

$[\alpha]^{22}_D$ = -50° (*c* = 0.02, CH_2Cl_2)

^{1}H-NMR (400 MHz, $CDCl_3$)

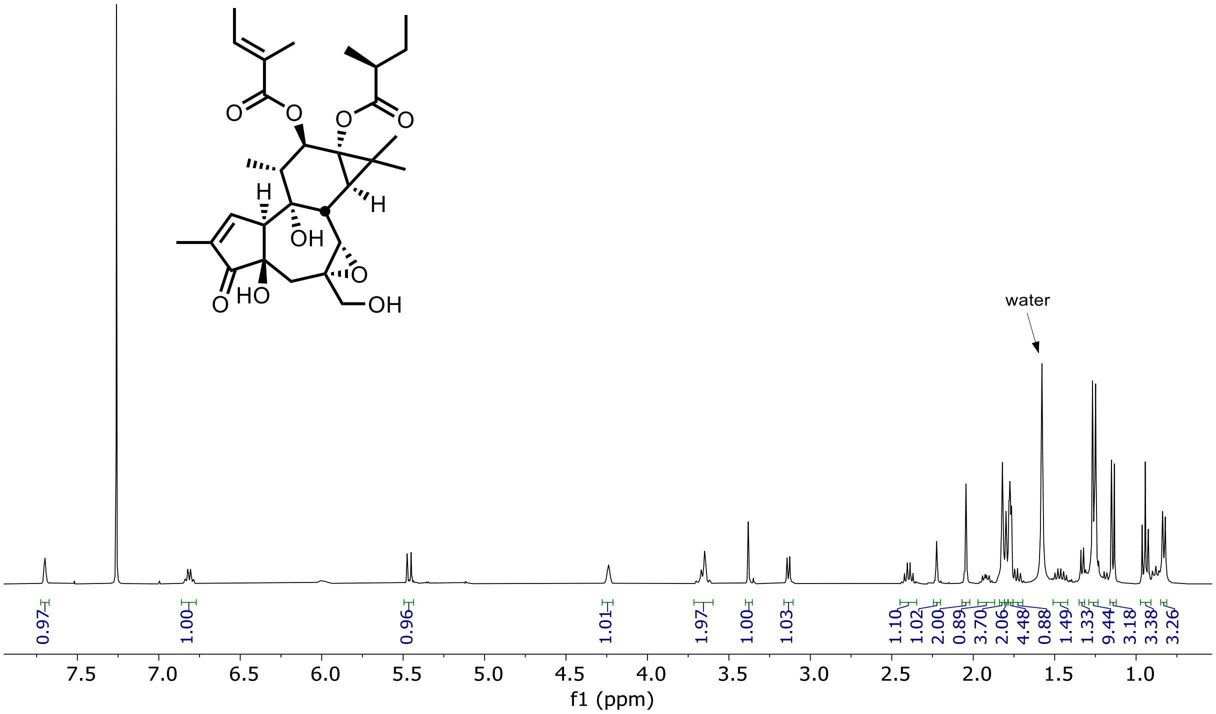

^{13}C-NMR (126 MHz, $CDCl_3$)

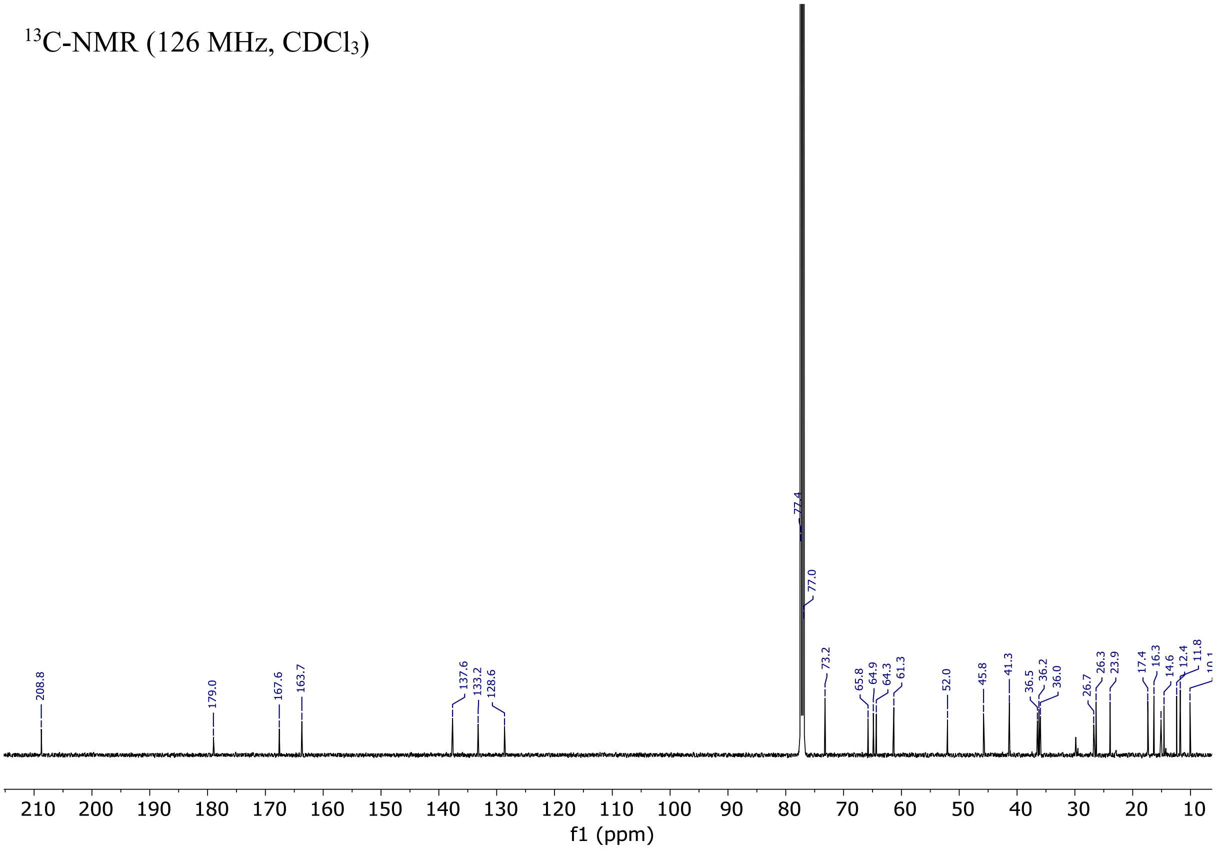

C13-diversifiable intermediate **4** preparation

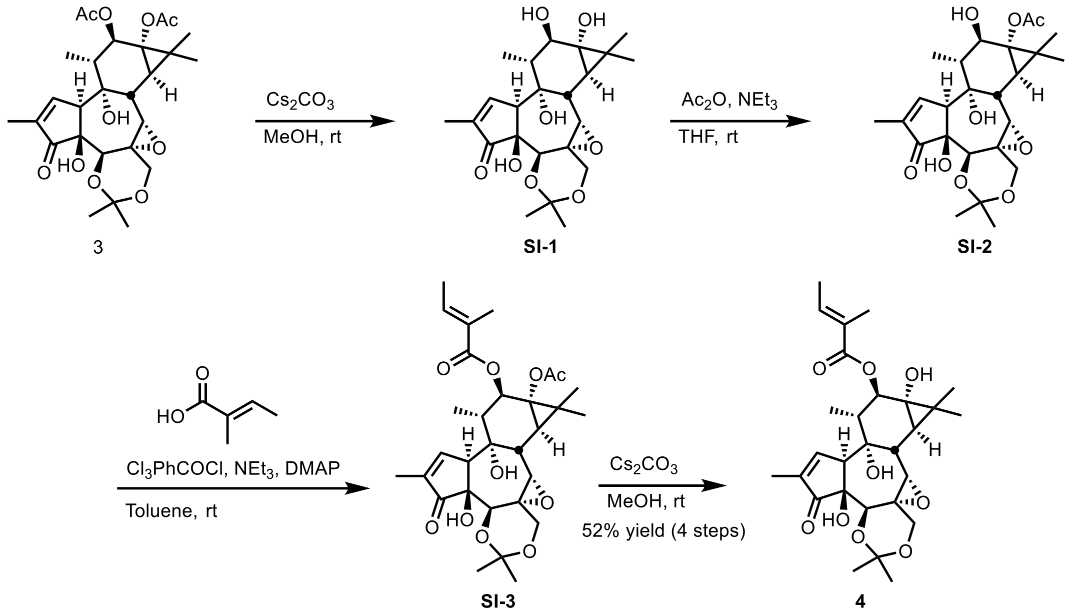

To a vial equipped with a stir bar was added **3** (97 mg, 0.186 mmol, 1.0 equiv). A solution of cesium carbonate (155 mg) was prepared in methanol (9.0 mL) and sonicated to dissolution. This basic solution of methanol was added directly to the reaction vessel as a single portion. The reaction mixture was stirred at rt for 18 hours. TLC analysis indicated complete conversion to intermediate **SI-1**. The reaction was quenched with sat. NH_4Cl (15 mL), diluted with brine (50 mL), and extracted with EtOAc (3x50 mL) until TLC of the aqueous layer no longer showed product. The resulting intermediate **SI-1** was used directly in the next step.

To a vial equipped with a stir bar was added crude residue **SI-1** followed by anhydrous THF (2 mL). Acetic anhydride (1.0 mL) and triethylamine (1.0 mL) were added directly each as one portion. The reaction was stirred at rt for 1 hour. TLC analysis indicated complete consumption of **SI-1**. The reaction was quenched with methanol (1 mL) and diluted with brine (20 mL). The aqueous layer was extracted with EtOAc (3x20 mL). The combined organic layers were dried over Na_2SO_4, filtered, and concentrated. The resulting intermediate **SI-2** was used directly in the next step.

To a flame dried vial equipped with a stir bar was added tiglic acid (136 mg), triethylamine (0.35 mL), and trichlorobenzoyl chloride (0.31 g) followed by anhydrous toluene (3.5 mL). This mixture was stirred vigorously at rt for 2 hours. **SI-2** (222 mg, 0.464 mmol, 1.0 equiv) was dissolved in anhydrous toluene (4.0 mL) in a separate vial equipped with a stir bar. The substrate solution was added to the previously described solution of in situ generated mixed anhydride in one portion followed by addition of DMAP (147 mg). The reaction mixture was stirred at rt for 2 hours. TLC analysis indicated complete consumption of **SI-2**. The reaction was diluted with EtOAc (100 mL) and washed with sat. NH_4Cl (50 mL), water (50 mL), sat. $NaHCO_3$ (50 mL), and brine (50 mL). The combined organic layers were dried over Na_2SO_4, filtered, and concentrated. The resulting intermediate **SI-3** was used directly in the next step.

To a vial equipped with a stir bar was added crude **SI-3**. A solution of cesium carbonate (300 mg) was prepared in methanol (18.0 mL) and sonicated to dissolution. This basic solution of methanol was added directly to the reaction vessel as a single portion. The reaction mixture was stirred at rt for 18 hours. TLC analysis indicated complete conversion to intermediate **4**. The reaction was quenched with sat. NH_4Cl (15 mL), diluted with brine (50 mL), and extracted with EtOAc (3x50 mL) until TLC of the aqueous layer no longer showed product. Purification was accomplished by silica gel flash column chromatography (10-40% EtOAc/Hex, 2x12 cm) affording diversifiable intermediate **4** (50.0 mg, 52% yield over 4 steps, 85% average yield). Compound purity was established by TLC (one spot) analysis.

SI-1 TLC R_f = 0.26 (50% Acetone/Hex, UV active, dark green spot in *p*-anisaldehyde)
SI-2 TLC R_f = 0.21 (50% EtOAc/Hex, UV active, green spot in *p*-anisaldehyde)
SI-3 TLC R_f = 0.55 (50% EtOAc/Hex, UV active, green spot in *p*-anisaldehyde)
4 TLC R_f = 0.20 (50% EtOAc/Hex, UV active, green spot in *p*-anisaldehyde)

^{1}H NMR (400 MHz, $CDCl_3$) δ 7.59 (dd, *J* = 2.6, 1.3 Hz, 1H), 6.86 (qq, *J* = 7.0, 1.3 Hz, 1H), 4.85 (d, *J* = 9.4 Hz, 1H), 4.73 (s, 1H), 4.11 (s, 1H), 3.98 (d, *J* = 13.0 Hz, 1H), 3.81 (app p, *J* = 2.3 Hz, 1H), 3.58 (d, *J* = 12.9 Hz, 1H), 3.32 (s, 1H), 3.05 (s, 1H), 2.98 (d, *J* = 7.5 Hz, 1H), 2.06 (s, 1H), 1.92 (dq, *J* = 9.4, 6.6 Hz, 1H), 1.82-1.79 (m, 6H), 1.78 (dd, *J* = 2.9, 1.3 Hz, 3H), 1.49 (s, 3H), 1.46 (s, 3H), 1.21 (s, 3H), 1.11 (d, *J* = 7.5 Hz, 1H), 1.05 (s, 3H), 1.01 (d, *J* = 6.6 Hz, 3H).

^{13}C NMR (126 MHz, $CDCl_3$, 28 peaks total) δ 205.8, 170.4, 160.5, 138.7, 134.7, 128.3, 101.4, 87.9, 77.9, 72.6, 68.5, 66.2, 65.7, 61.2, 61.1, 50.9, 45.8, 37.0, 34.8, 28.3, 24.8, 22.4, 22.3, 17.5, 16.9, 14.7, 12.2, 10.1.

HRMS calculated for $C_{28}H_{39}O_9^+$ $[M+H]^+$: 519.2589; found 519.2579.

FTIR (ATR) 3461 (br), 2978, 2931, 2865, 1704, 1689, 1648, 1457, 1375, 1259, 1223, 1088, 1022, 922, 881, 831, 739, 669 cm^{-1}

$[\alpha]^{24}_D$ = 25° (*c* = 0.40, CH_2Cl_2)

^{1}H-NMR (400 MHz, $CDCl_3$)

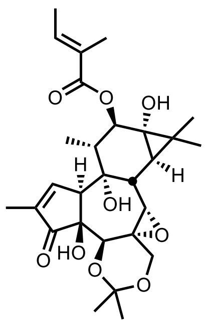

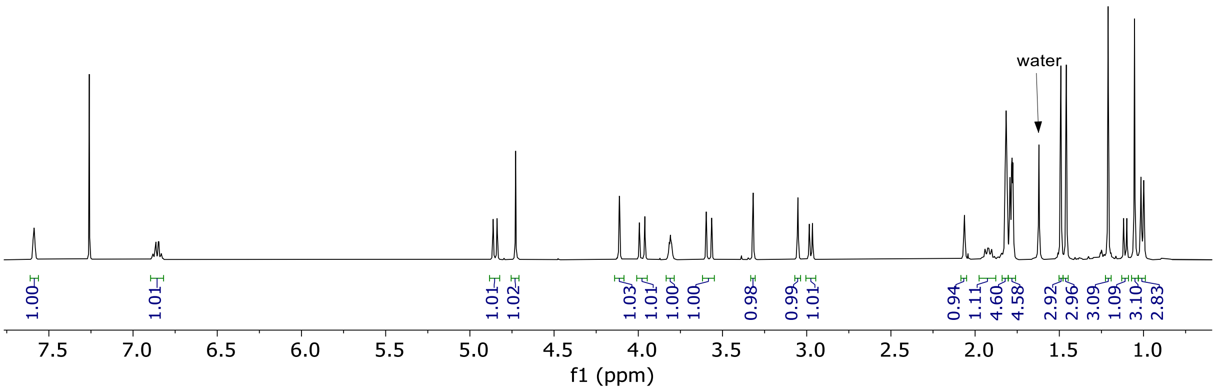

^{13}C-NMR (126 MHz, $CDCl_3$)

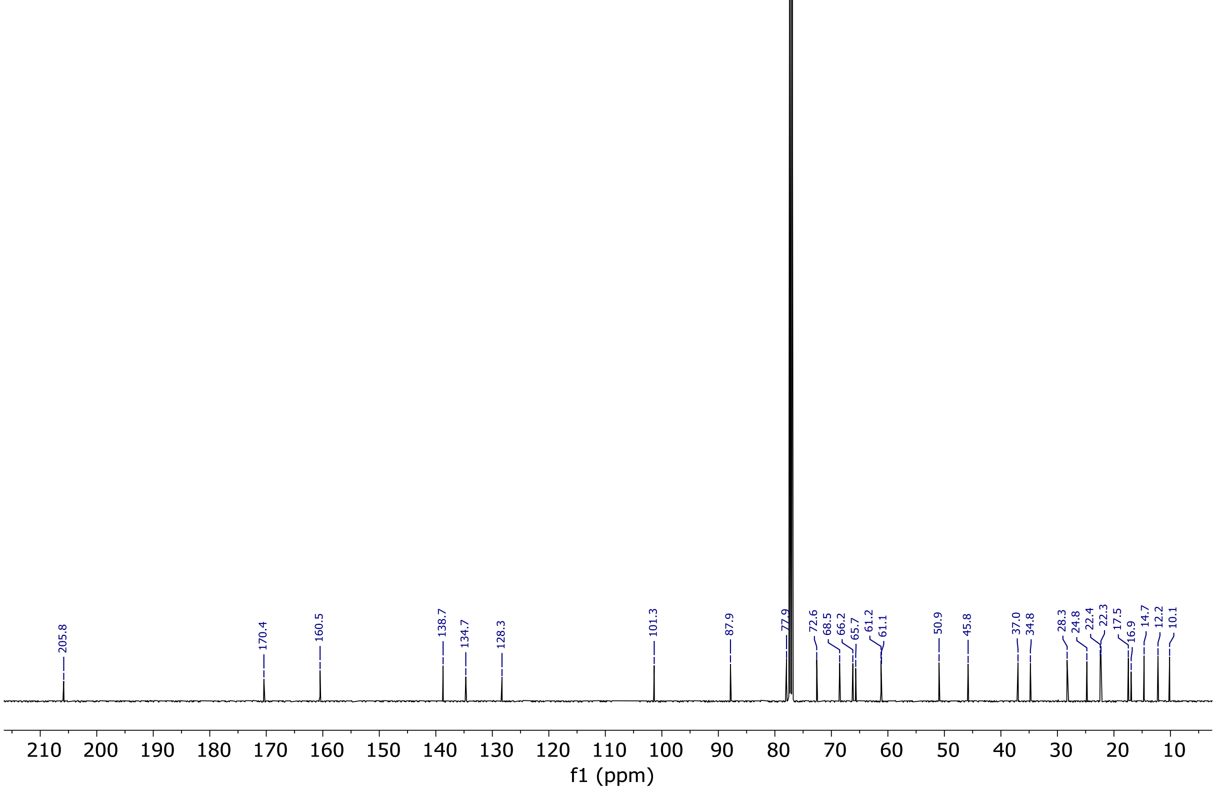

SUW406 preparation (esterification and deprotection)

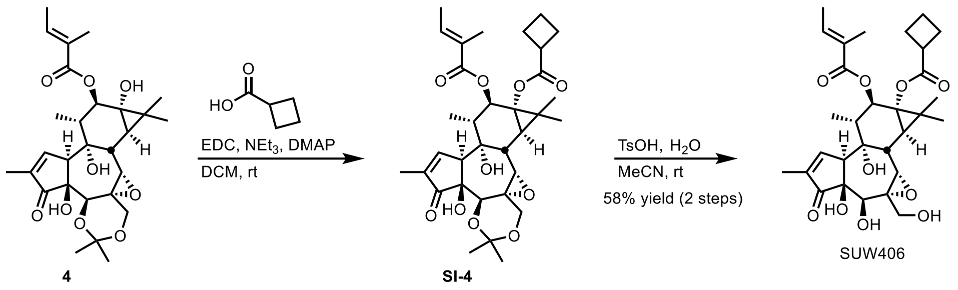

In a flame-dried vial, EDC (23.7 mg, 123 µmol, 2 equiv), triethylamine (25.8 µL, 185 µmol, 3 equiv), cyclobutanecarboxylic acid (11.1 mg, 111 µmol, 1.8 equiv), and DMAP (1.9 mg, 15.4 µmol, 0.25 equiv) were dissolved in anhydrous DCM (1.0 mL) and sonicated until homogeneous. In a separate flame-dried vial, **4** (32.0 mg, 61.7 µmol, 1 equiv) was dissolved in anhydrous THF (1.0 mL) and the DCM solution of activated acid was added directly in one portion. The reaction was stirred at rt for 34 hours. TLC analysis indicated complete consumption of **4** and conversion to cyclobutane ester **SI-4**. The reaction was quenched with methanol (0.5 mL) and diluted with brine (5 mL) and sat. $NaHCO_3$ (5 mL). The aqueous layer was extracted with EtOAc (3x10 mL). The combined organic layers were dried over Na_2SO_4, filtered, and concentrated. The resulting intermediate **SI-4** was used directly in the next step.

In a vial equipped with a stir bar, the crude **SI-4** was dissolved in 2 mL TsOH in MeCN (0.21 M). The reaction was stirred at rt for 18 hours. TLC analysis indicated complete consumption of **SI-4**. The reaction was quenched with sat. $NaHCO_3$ (5 mL) and diluted with brine (5 mL). The aqueous layer was extracted with EtOAc (3x10 mL). Solid NaCl (1 g) was added to the aqueous layer during each EtOAc extraction to remove product from the aqueous layer. The combined organic layers were dried over Na_2SO_4, filtered, and concentrated. Purification was accomplished by silica gel flash column chromatography (30-70% EtOAc/Hex, 1x10 cm) affording SUW406 (20.0 mg, 58%, 2 steps) as a white solid. Compound purity was established by TLC (one spot) analysis.

SUW406 TLC R_f = 0.41 (70% EtOAc/Hex, UV active, green spot in *p*-anisaldehyde)

^{1}H NMR (400 MHz, $CDCl_3$) δ 7.73 (app s, 1H), 6.82 (qq, *J* = 7.0, 1.5 Hz, 1H), 6.04 (br s, 1H), 5.45 (d, *J* = 9.9 Hz, 1H), 4.24 (s, 1H), 4.09 (app p, *J* = 2.8 Hz, 1H), 3.95 (br s, 1H), 3.89-3.79 (m, 2H), 3.67 (s, 1H), 3.28 (s, 1H), 3.22-3.11 (m, 2H), 2.44-2.13 (m, 5H), 2.03-1.87 (m, 3H), 1.82 (app s, 3H), 1.79 (app d, *J* = 7.1 Hz, 3H), 1.76 (dd, *J* = 2.9, 1.3 Hz, 3H), 1.31 (d, *J* = 6.6 Hz, 1H), 1.26 (s, 3H), 1.22 (s, 3H), 0.87 (d, *J* = 6.5 Hz, 3H).

^{13}C NMR (126 MHz, $CDCl_3$, 30 peaks total) δ 210.1, 177.8, 167.7, 164.9, 137.8, 133.6, 128.6, 77.4, 76.9, 72.6, 71.6, 65.7, 65.4, 64.7, 61.9, 49.1, 46.0, 38.1, 36.23, 36.15, 26.7, 24.92, 24.91, 23.7, 18.5, 17.3, 15.2, 14.6, 12.3, 9.9.

HRMS calculated for $C_{30}H_{41}O_{10}^+$ $[M+H]^+$: 561.2700; found 561.2688.

FTIR (ATR) 3404 (br), 2952, 2925, 2870, 1709, 1377, 1255, 1155, 1134, 1074, 1022, 980, 930, 802, 731 cm^{-1}

$[\alpha]^{25}_{D}$ = -14° (*c* = 0.40, CH_2Cl_2)

^{1}H-NMR (400 MHz, $CDCl_3$)

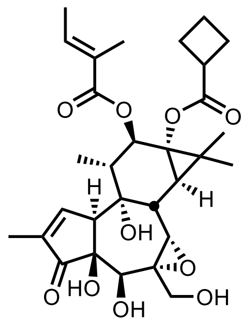

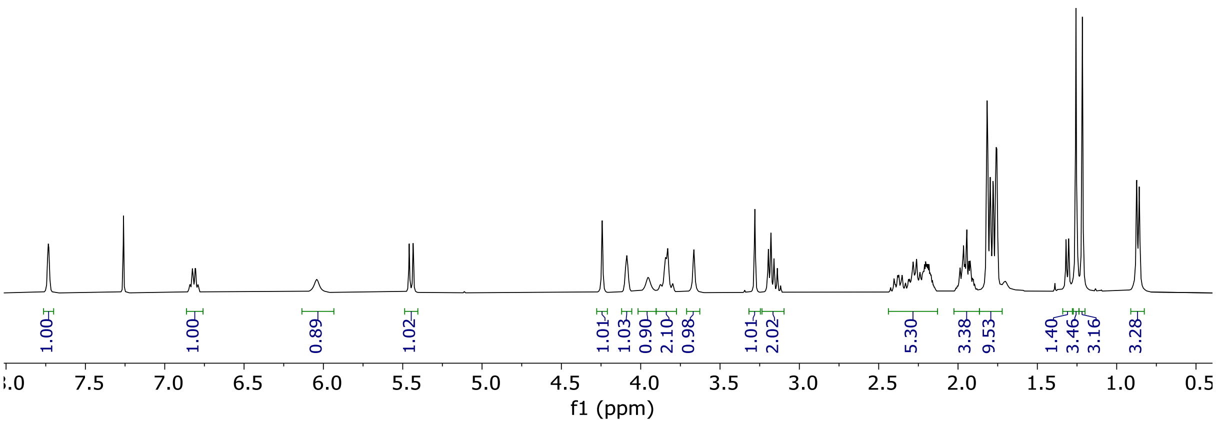

^{13}C-NMR (126 MHz, $CDCl_3$)

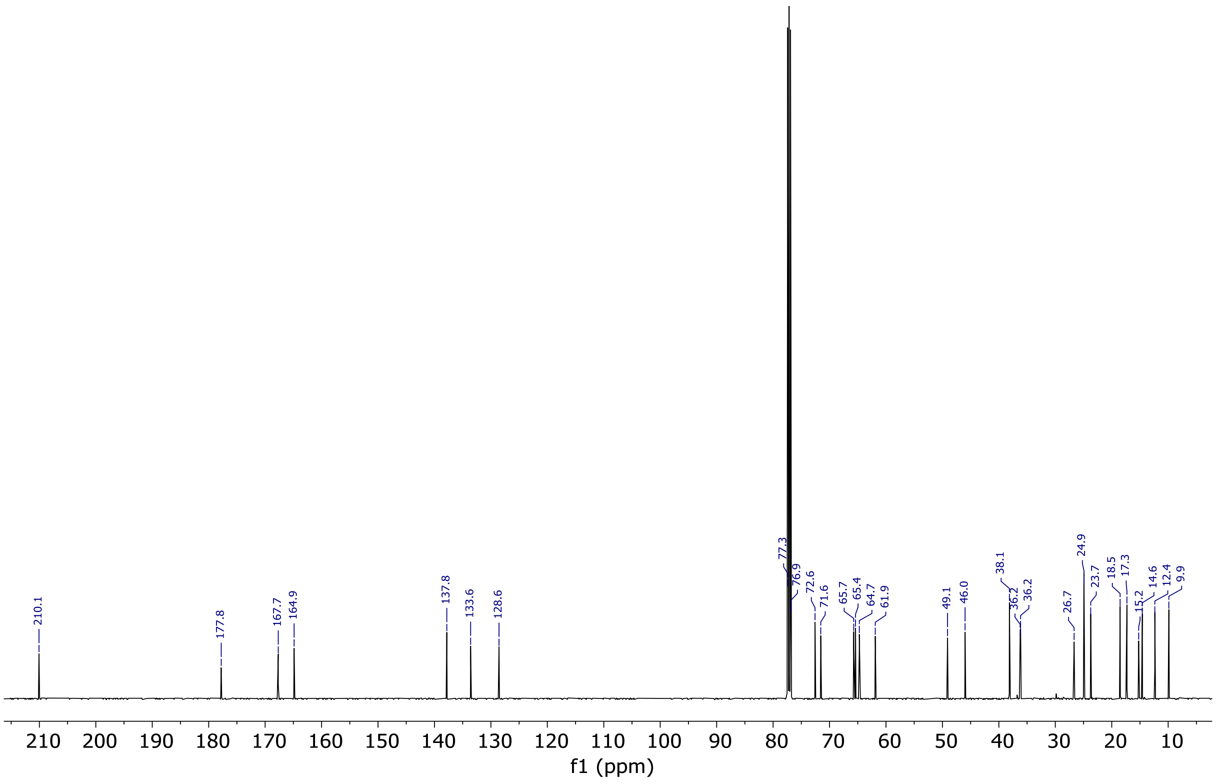

SUW407 preparation (esterification and deprotection)

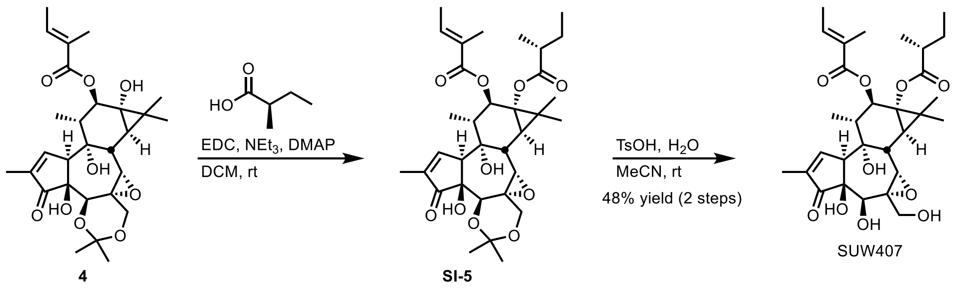

In a flame-dried vial, EDC (20.4 mg, 106 µmol, 2 equiv), triethylamine (22.3 µL, 160 µmol, 3 equiv), (R)-2-methylbutanoic acid (9.8 mg, 96 µmol, 1.8 equiv), and DMAP (1.6 mg, 13 µmol, 0.25 equiv) were dissolved in anhydrous DCM (1.0 mL) and sonicated until homogeneous. In a separate flame-dried vial, **4** (27.6 mg, 53.2 µmol, 1 equiv) was dissolved in anhydrous THF (1.0 mL) and the DCM solution of activated acid was added directly in one portion. The reaction was stirred at rt for 32 hours. TLC analysis indicated complete consumption of **4** and conversion to methyl butanoate ester **SI-5**. The reaction was quenched with methanol (0.5 mL) and diluted with brine (5 mL) and sat. $NaHCO_3$ (5 mL). The aqueous layer was extracted with EtOAc (3x10 mL). The combined organic layers were dried over Na_2SO_4, filtered, and concentrated. The resulting intermediate **SI-5** was used directly in the next step.

In a vial equipped with a stir bar, crude **SI-5** was dissolved in MeCN (1 mL). TsOH in water (0.42 M, 1 mL) was added (final TsOH concentration of 0.21 M). The reaction was stirred at rt for 18 hours. TLC analysis indicated complete consumption of **SI-5**. The reaction was quenched with sat. $NaHCO_3$ (5 mL) and diluted with brine (5 mL). The aqueous layer was extracted with EtOAc (3x10 mL). Solid NaCl (1 g) was added to the aqueous layer during each EtOAc extraction to remove product from the aqueous layer. The combined organic layers were dried over Na_2SO_4, filtered, and concentrated. Purification was accomplished by silica gel flash column chromatography (30-70% EtOAc/Hex, 1x10 cm) affording SUW407 (14.4 mg, 48%, 2 steps) as a white solid. Compound purity was established by TLC (one spot) analysis.

SUW407 TLC R_f = 0.43 (70% EtOAc/Hex, UV active, green spot in *p*-anisaldehyde)

^{1}H NMR (400 MHz, $CDCl_3$) δ 7.74 (dd, *J* = 2.7, 1.4 Hz, 1H), 6.82 (qq, *J* = 7.0, 1.5 Hz, 1H), 6.06 (br s, 1H), 5.44 (d, *J* = 9.9 Hz, 1H), 4.24 (d, *J* = 3.1, 1H), 4.08 (app p, *J* = 2.6, 1H), 3.90 (dd, *J* = 12.5, 5.8 Hz, 1H), 3.86 (d, *J* = 3.2 Hz, 1H), 3.80 (dd, *J* = 12.5, 5.8 Hz, 1H), 3.58 (d, *J* = 1.1 Hz, 1H), 3.29 (s, 1H), 3.19 (d, *J* = 6.7 Hz, 1H), 2.41 (app sex, *J* = 6.9 Hz, 1H), 2.15 (dd, *J* = 8.0, 6.0 Hz, 1H), 1.97 (dq, *J* = 10.0, 6.5 Hz, 1H), 1.83 (t, *J* = 1.2 Hz, 3H), 1.79 (dd, 7.1, 1.0 Hz, 3H), 1.77 (dd, *J* = 2.9, 1.3 Hz, 3H), 1.75-1.64 (m, 1H), 1.52-1.41 (m, 1H), 1.29 (d, *J* = 6.7 Hz, 1H), 1.27 (s, 3H), 1.24 (s, 3H), 1.19 (d, *J* = 7.0 Hz, 3H), 0.90 (t, *J* = 7.5 Hz, 3H), 0.87 (d, *J* = 6.3 Hz, 3H).

^{13}C NMR (126 MHz, $CDCl_3$, 30 peaks total) δ 210.1, 179.3, 167.7, 164.9, 137.8, 133.6, 128.6, 77.3, 76.8, 72.5, 71.8, 65.7, 65.4, 64.7, 61.8, 49.1, 46.1, 41.2, 36.4, 36.2, 26.6, 26.5, 23.9, 17.4, 16.3, 15.3, 14.6, 12.4, 11.8, 9.9.

HRMS calculated for $C_{30}H_{43}O_{10}^+$ $[M+H]^+$: 563.2856; found 563.2849.

FTIR (ATR) 3402 (br), 2963, 2925, 2880, 1711, 1459, 1379, 1340, 1327, 1257, 1195, 1155, 1131, 1076, 1024, 978, 933, 802, 730 cm^{-1}

[α]^{25}$_{D}$ = -15° (*c* = 0.20, CH_2Cl_2)

^{1}H-NMR (400 MHz, $CDCl_3$)

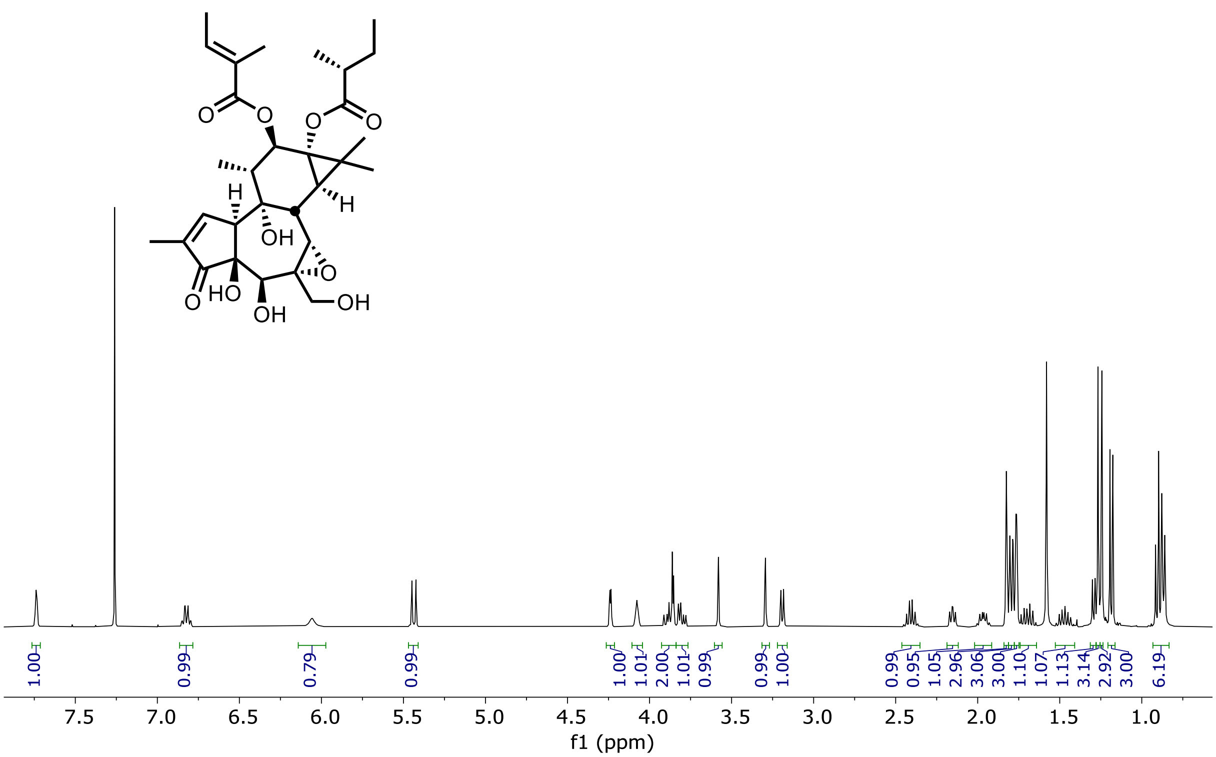

^{13}C-NMR (101 MHz, $CDCl_3$)

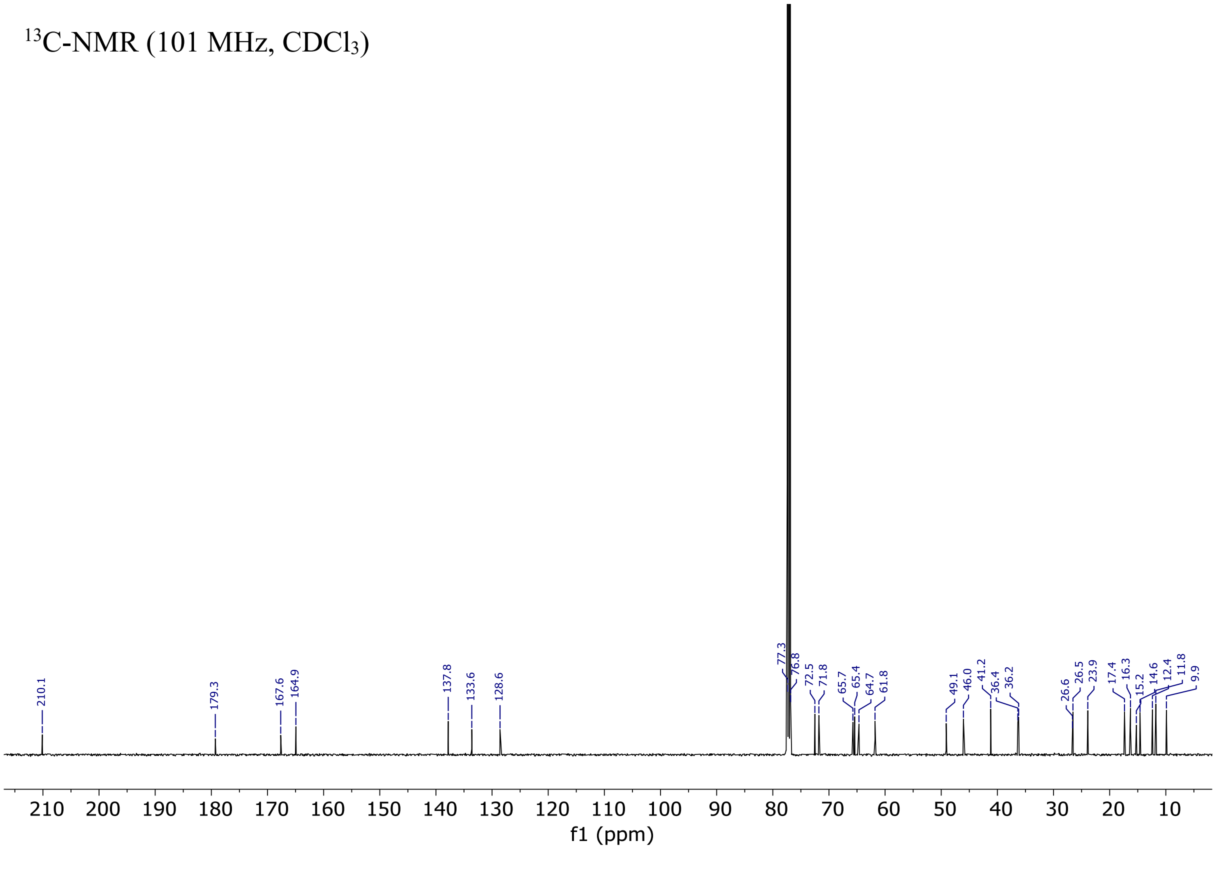

SUW413 preparation (esterification and deprotection)

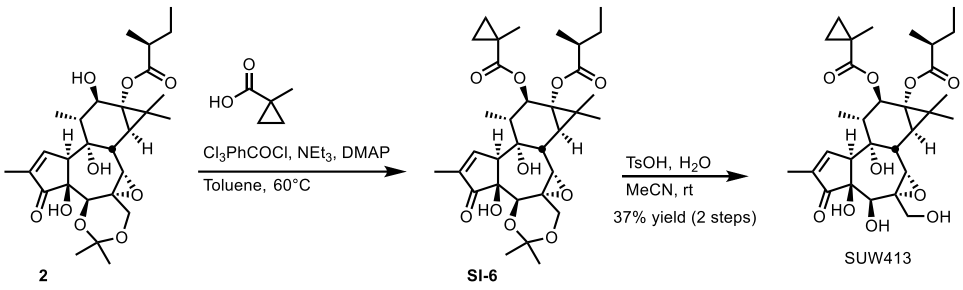

To a 2-dram vial was added cyclopropyl acid (12.7 mg, 0.127 mmol, 2.2 equiv) followed by anhydrous toluene (0.5 mL). Triethylamine (32 µL, 0.230 mmol, 4 equiv) was added in one portion followed by 2,4,6-trichlorobenzoyl chloride (18 µL, 0.115 mmol, 2 equiv). This mixture was stirred vigorously at rt for 2 hours. In a separate vial, **2** (30.0 mg, 0.0576 mmol, 1 equiv) was dissolved in anhydrous toluene (0.5 mL). The substrate solution was added to the previously described solution of in situ generated mixed anhydride in one portion followed by addition of DMAP (18.3 mg, 0.150 mmol, 2.6 equiv). The reaction mixture was stirred at 60 °C for 18 hr. TLC analysis indicated complete consumption of **2**. The reaction was diluted with sat. $NaHCO_3$ (10 mL) and extracted with DCM (3x10 mL). The combined organic layers were dried over Na_2SO_4, filtered, and concentrated. The resulting intermediate **SI-6** was used directly in the next step.

In a vial equipped with a stir bar, crude **SI-6** was dissolved in MeCN (1 mL). Tosic acid monohydrate (67.0 mg, 0.352 mmol, 6.11 equiv) in water (0.3 mL) was added to the reaction mixture in one portion. The reaction mixture was stirred at rt for 18 hr. TLC analysis indicated complete consumption of **SI-6**. The reaction was quenched with sat. $NaHCO_3$ (10 mL) and extracted with DCM (3x10 mL). The combined organic layers were dried over Na_2SO_4, filtered, and concentrated. Purification was accomplished by silica gel flash column chromatography (20%-60% EtOAc/Hex, 3x10 cm) affording SUW413 (12.0 mg, 37% over 2 steps) as a white solid. Compound purity was established by TLC (one spot) analysis.

SUW413 TLC R_f = 0.27 (60% EtOAc/Hex, UV active, green spot in *p*-anisaldehyde)

^{1}H NMR (400 MHz, $CDCl_3$) δ 7.73 (dd, *J* = 2.6, 1.5 Hz, 1H), 5.37 (d, *J* = 9.9 Hz, 1H), 4.23 (s, 1H), 4.06 (app p, *J* = 2.8 Hz, 1H), 3.88 (d, *J* = 12.5 Hz, 1H), 3.79 (d, *J* = 12.5 Hz, 1H), 3.28 (s, 1H), 3.16 (d, *J* = 6.6 Hz, 1H), 2.38 (app sex, *J* = 6.9 Hz, 1H), 1.88 (dq, *J* = 10.0, 6.6 Hz, 1H), 1.77 (dd, *J* = 2.6, 1.5 Hz, 3H), 1.75-1.68 (m, 1H), 1.51-1.39 (m, 1H), 1.28 (s, 3H), 1.26 (s, 3H), 1.23 (s, 3H), 1.18 (d, *J* = 3.7 Hz, 2H), 1.13 (d, *J* = 7.0 Hz, 3H), 0.93 (t, *J* = 7.4 Hz, 3H), 0.86 (d, *J* = 6.5 Hz, 3H), 0.71 – 0.64 (m, 2H).

^{13}C NMR (126 MHz, $CDCl_3$, 30 peaks total) δ 210.1, 179.0, 175.5, 164.9, 133.7, 77.4, 76.9, 72.5, 71.8, 65.6, 65.4, 64.7, 61.8, 49.1, 46.0, 41.3, 36.19, 36.17, 26.7, 26.4, 23.9, 19.7, 18.6, 17.3, 16.9, 16.6, 16.3, 15.2, 11.8, 9.9.

HRMS calculated for $C_{30}H_{42}NaO_{10}^+$ [M+Na]$^+$: 585.2676; found: 585.2666.

FTIR (ATR) 3411 (br), 2961, 2925, 2854, 1716, 1458, 1379, 1322, 1261,1159, 1086, 1026, 802, 669 cm^{-1}

$[\alpha]^{24}_D$ = 18° (*c* = 0.05, CH_2Cl_2)

^{1}H-NMR (400 MHz, $CDCl_3$)

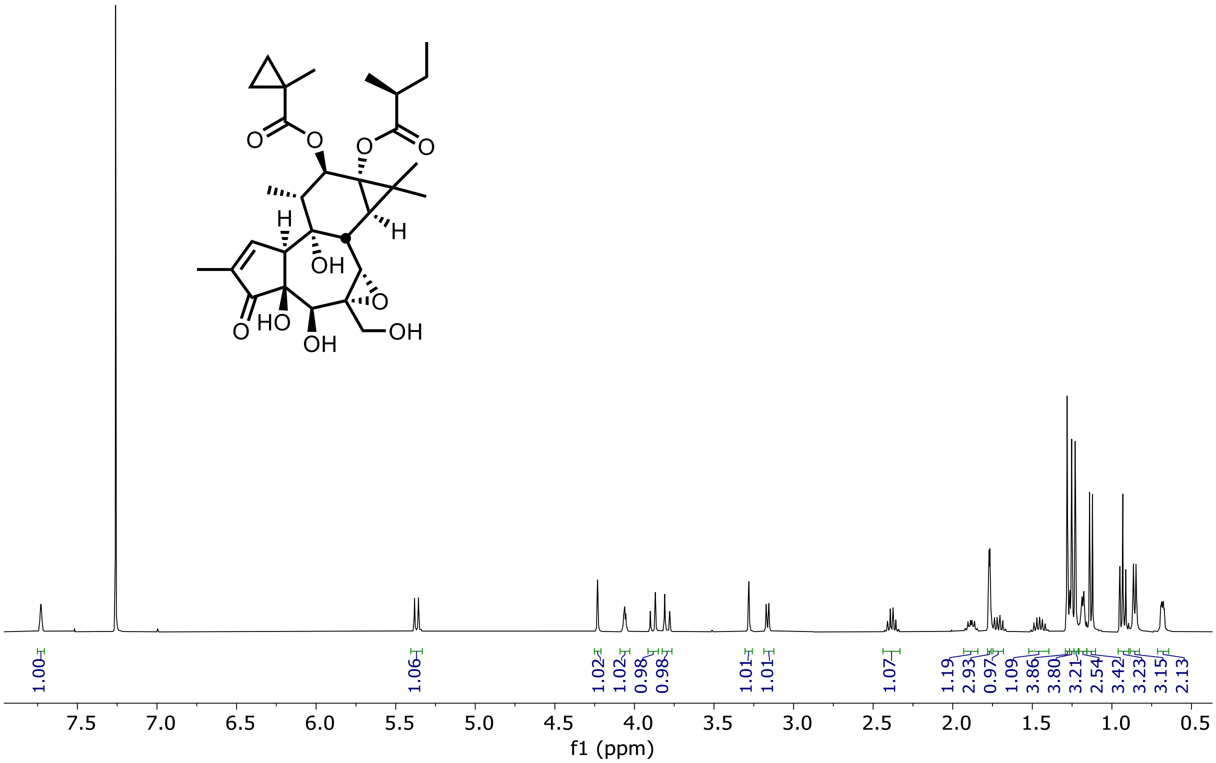

^{13}C-NMR (126 MHz, $CDCl_3$)

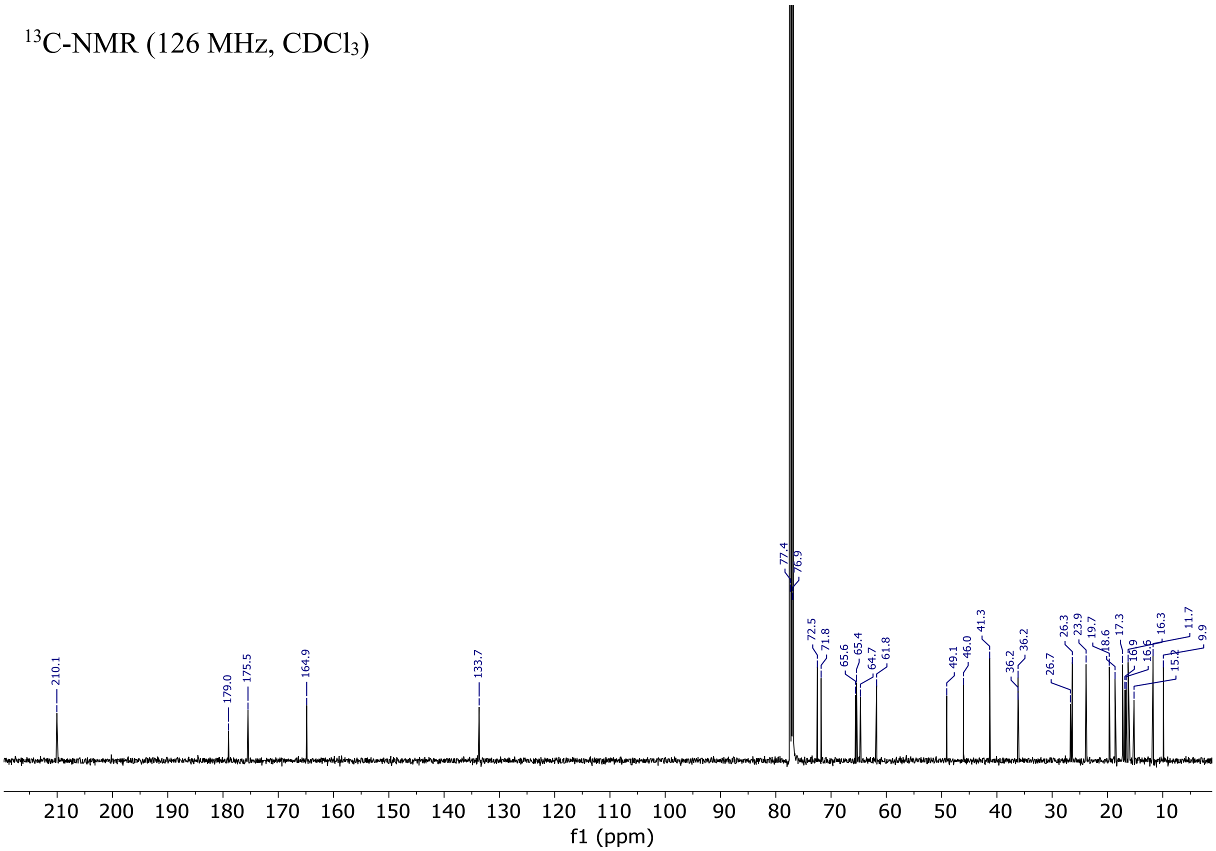

Compound **6** preparation (TBS protection)

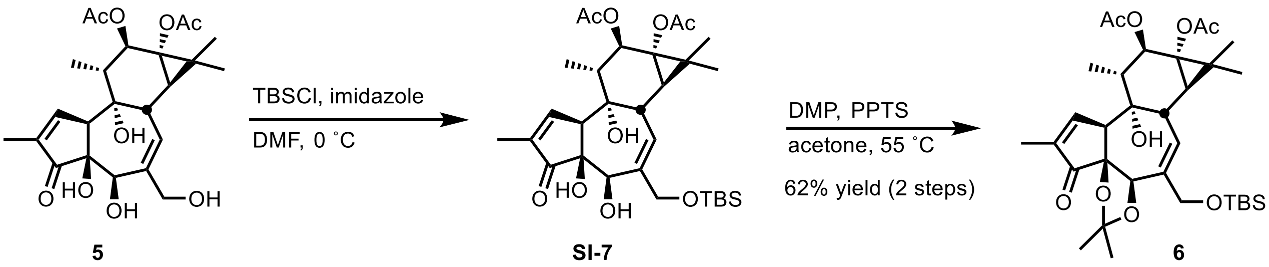

To a one-neck, round-bottom flask was added **5** (2.70 g, 5.8 mmol, 1.0 equiv) followed by anhydrous DMF (58.0 mL). Imidazole (1.58 g, 23.25 mmol, 4.0 equiv) was added as single portion followed by TBS-Cl (2.6 g, 17.44 mmol, 3.0 equiv). The reaction was stirred at 0 ˚C and allowed to warm to room temperature for 20 minutes. TLC analysis indicated complete consumption of intermediate **5**. The reaction was quenched with methanol (5 mL) and diluted with sat. NH_4Cl (50 mL) and diluted with brine (250 mL). The aqueous layer was extracted with EtOAc (2x150 mL). The combined organic layers were dried over Na_2SO_4, filtered, and concentrated. Residual DMF was removed by azeotroping with heptane (2x100 mL). The resulting intermediate **SI-7** was used directly in the next step.

To a 500 mL round-bottom flask equipped with a stir bar was added crude **SI-7**. Anhydrous acetone (65 mL) and PPTS (1.46g, 5.8 mmol) were added to the reaction mixture each as one portion. The reaction mixture was sonicated for five minutes to dissolve the PPTS. Afterwards, dimethoxypropane (130 mL) was added directly as a single portion. The flask was equipped with a Vigreux condenser and an argon balloon, and the reaction mixture was heated to 60 ˚C for 2 hr. TLC analysis indicated complete consumption of intermediate **SI-7**. The reaction was quenched with sat. $NaHCO_3$ (100 mL) and diluted with brine (250 mL). The aqueous layer was extracted with EtOAc (2x250 mL). The combined organic layers were dried over Na_2SO_4, filtered, and concentrated. Purification was accomplished by silica gel flash column chromatography (20-30% Et_2O/Hex, 3x13 cm) affording 4,5-acetonide **6** as a white foam (2.5g, 62% yield over 2 steps). Compound purity was established by TLC (one spot) analysis.

SI-7 TLC R_f = 0.84 (75% EtOAc/Hex, UV active, green spot in *p*-anisaldehyde)
6 TLC R_f = 0.95 (20% EtOAc/Et_2O, UV active, blue spot in *p*-anisaldehyde)

^{1}H NMR (400 MHz, $CDCl_3$) δ 7.56 (dd, *J* = 2.3, 1.4 Hz, 1H), 5.80 (dd, *J* = 6.7, 1.6 Hz, 1H), 5.74 (br s, 1H), 5.46 (d, *J* = 10.2 Hz, 1H), 4.88 (d, *J* = 1.5 Hz, 1H), 4.21-4.12 (m, 2H), 3.13 (app p, *J* = 2.9 Hz, 1H), 2.80 (app t, *J* = 6.0 Hz, 1H), 2.11 (s, 3H), 2.10 (s, 3H), 1.94 (dq, *J* = 10.4, 6.4 Hz, 1H), 1.77 (dd, *J* = 3.1, 1.4 Hz, 3H), 1.53 (s, 3H), 1.50 (s, 3H), 1.30 (s, 3H), 1.20 (s, 3H), 1.14 (d, *J* = 5.3 Hz, 1H), 0.99 (d, *J* = 6.5 Hz, 3H), 0.85 (s, 9H), 0.06 (s, 3H), 0.03 (s, 3H).

^{13}C NMR (126 MHz, $CDCl_3$, 33 peaks total) δ 204.8, 173.9, 171.1, 158.1, 141.5, 133.6, 133.3, 110.1, 84.5, 77.3, 75.2, 74.6, 66.7, 66.0, 55.1, 44.8, 39.3, 36.4, 26.9, 26.5, 26.0 (3 peaks), 25.4, 24.3, 21.2, 21.2, 18.6, 18.4, 14.7, 10.6, -5.16, -5.22.

HRMS calculated for $C_{33}H_{50}NaO_9Si^+$ [M+Na]$^+$: 641.3098; found: 641.3116.

FTIR (ATR) 3384 (br), 2957, 2922, 2852, 1740, 1716, 1663, 1464, 1377, 1261, 1230, 1090, 1020, 838, 800 cm^{-1}

$[\alpha]^{24}_{D}$ = 80° (*c* = 0.02, CH_2Cl_2)

^{1}H-NMR (400 MHz, $CDCl_3$)

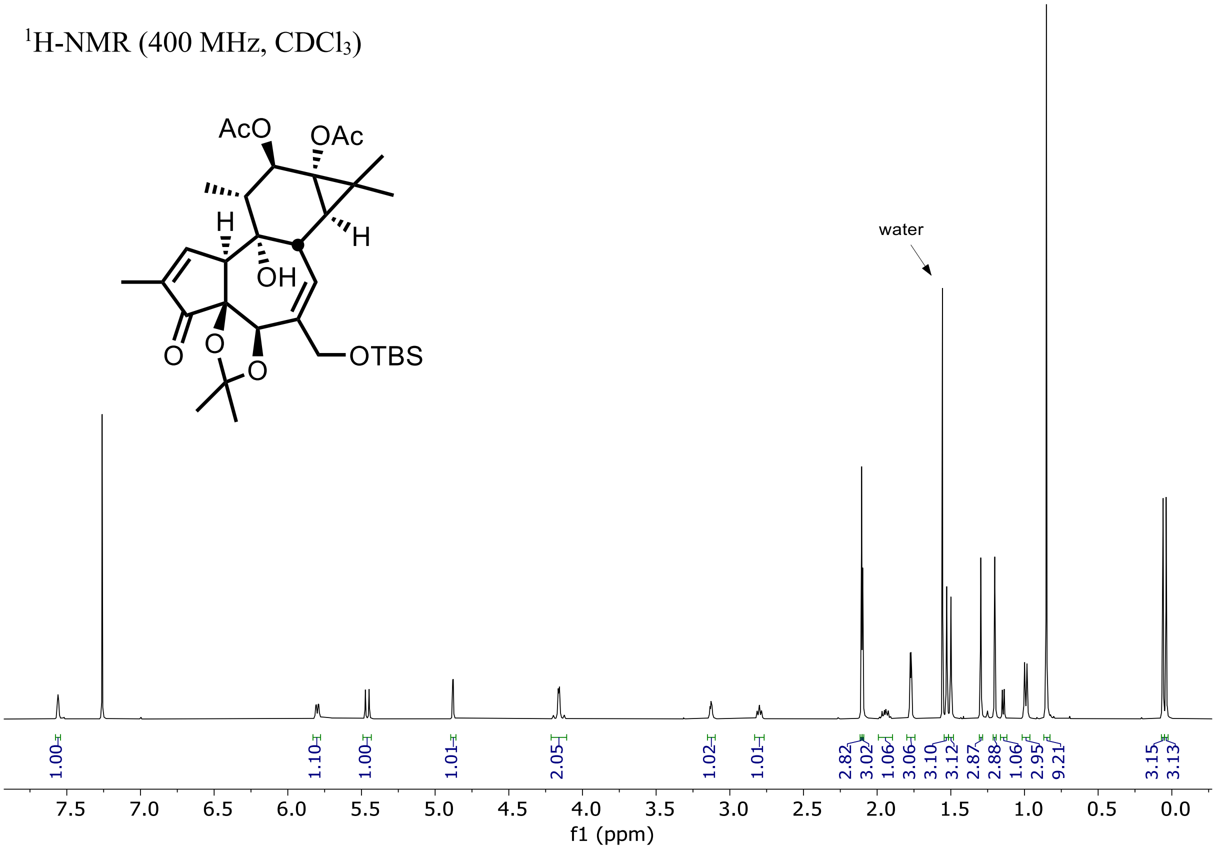

^{13}C-NMR (126 MHz, $CDCl_3$)

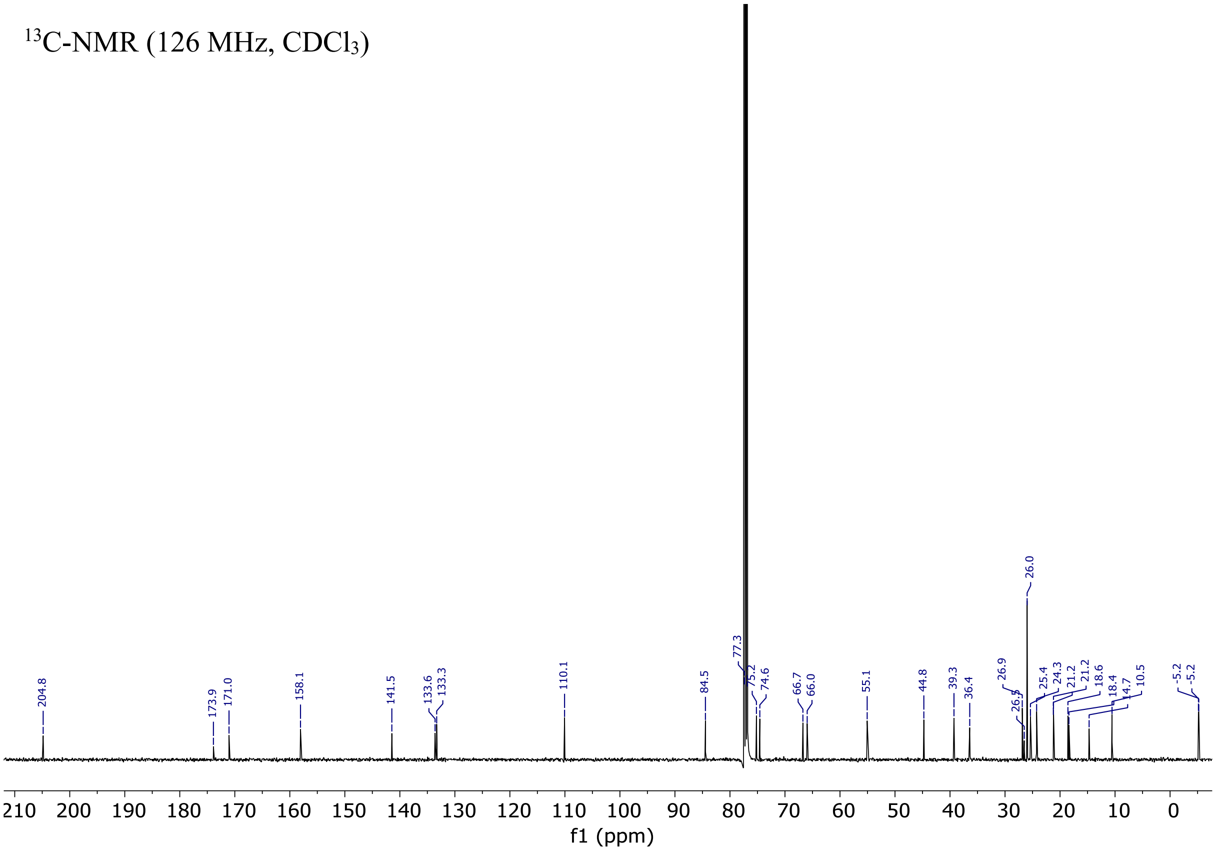

Compound **7** preparation (acetate removal and methyl butyration)

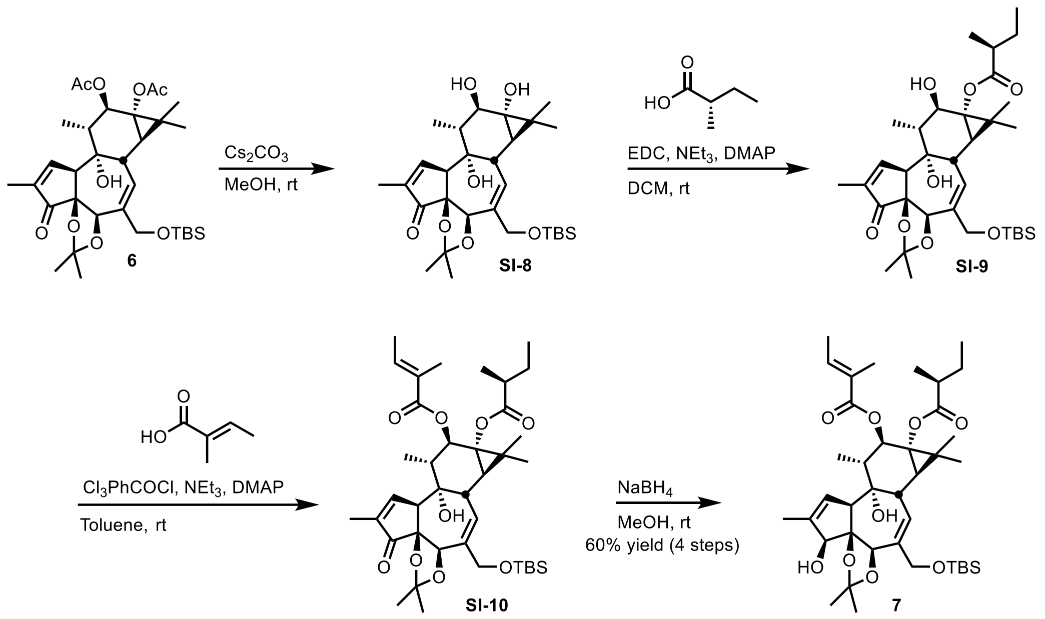

To a vial equipped with a stir bar was added **6** (623 mg, 1.00 mmol, 1.0 equiv). A solution of cesium carbonate (128 mg) was prepared in methanol (9.5 mL) and sonicated to dissolution. This basic solution of methanol was added directly to the reaction vessel as a single portion. The reaction mixture was stirred at rt for 18 hours. TLC analysis indicated complete conversion to intermediate **SI-8**. The reaction was quenched with sat. NH_4Cl (25 mL), diluted with brine (75 mL), and extracted with EtOAc (3x100 mL) until TLC of the aqueous layer no longer showed product. The resulting intermediate **SI-8** was used directly in the next step.

In a flame-dried vial, EDC (396 mg, 2.06 mmol, 2.05 equiv), triethylamine (300 µL, 2.12 µmol, 2.1 equiv), (S)-2-methylbutanoic acid (220 µL, 2.02 mmol, 2.0 equiv), and DMAP (25 mg, 202 µmol, 0.2 equiv) were dissolved in anhydrous DCM (5.0 mL) and sonicated until homogeneous. In a separate vial equipped with a stir bar, crude **SI-8** was dissolved in anhydrous DCM (5.0 mL) and the DCM solution of activated acid was added directly in one portion. The reaction was stirred at rt for 1 hour. TLC analysis indicated complete consumption of **SI-8** and conversion to methyl butanoate **SI-9**. The reaction was quenched with methanol (5 mL) and diluted with brine (50 mL) and 1 M HCl (1.0 mL). The aqueous layer was extracted with DCM (3x50 mL). The combined organic layers were dried over Na_2SO_4, filtered, and concentrated. The resulting intermediate **SI-9** was used directly in the next step.

To a flame dried vial equipped with a stir bar was added tiglic acid (333 mg, 3.33 mmol, 3.3 equiv), triethylamine (0.840 mL, 6.06 mmol, 6.0 equiv), and trichlorobenzoyl chloride (0.470 mL, 1.56 mmol, 3.0 equiv) followed by anhydrous toluene (10.0 mL). This mixture was stirred vigorously at rt for 2 hours. Crude **SI-9** was dissolved in anhydrous toluene (2.0 mL) in a separate vial equipped with a stir bar. The substrate solution was added to the previously described solution of in situ generated mixed anhydride in one portion followed by addition of DMAP (382 mg, 3.13 mmol, 3.1 equiv). The reaction mixture was stirred at rt for 2 hours. TLC analysis indicated complete consumption of monoester **SI-9**. The reaction was diluted with EtOAc (200 mL) and

washed with sat. NH_4Cl (100 mL), water (2x100 mL), sat. $NaHCO_3$ (100 mL), and brine (100 mL). The combined organic layers were dried over Na_2SO_4, filtered, and concentrated. The resulting intermediate **SI-10** was used directly in the next step.

In a vial equipped with a stir bar, crude **SI-10** was dissolved in MeOH (10.0 mL). Sodium borohydride (120 mg, 3.17 mmol, 3.1 equiv) was added directly to the vial as a single portion. The reaction was stirred at rt for 15 minutes. TLC analysis indicated complete consumption of diester **SI-10**. The reaction was diluted with water (50 mL) and brine (50 mL) and extracted with ethyl acetate (2x100 mL). The combined organic layers were dried over Na_2SO_4, filtered, and concentrated. Purification was accomplished by silica gel flash column chromatography (10-30% Et_2O/Hex, 2x12 cm) affording **7** as a white foam (426 mg, 60% yield over 4 steps, 88% average yield per step). Compound purity was established by TLC (one spot) analysis.

SI-8 TLC R_f = 0.24 (20% EtOAc/Et_2O, UV active, blue spot in *p*-anisaldehyde)
SI-9 TLC R_f = 0.28 (40% Et_2O/Hex, UV active, blue spot in *p*-anisaldehyde)
SI-10 TLC R_f = 0.67 (40% Et_2O/Hex, UV active, blue spot in *p*-anisaldehyde)
7 TLC R_f = 0.51 (40% Et_2O/Hex, UV active, blue spot in *p*-anisaldehyde)

^{1}H NMR (400 MHz, $CDCl_3$) δ 6.82 (dq, *J* = 7.1, 1.5 Hz, 1H), 5.86-5.77 (m, 3H), 5.51 (d, *J* = 9.9 Hz, 1H), 4.77 (d, *J* = 1.6 Hz, 1H), 4.24 (d, *J* = 12.1, 1H), 4.19 (d, *J* = 12.9 Hz, 1H), 4.13 (d, *J* = 12.2 Hz, 1H), 3.08-3.00 (m, 2H), 2.37 (app sex, *J* = 7.0 Hz, 1H), 1.90 (dq, *J* = 9.8, 6.5 Hz, 1H), 1.83 (s, 3H), 1.82-1.78 (m, 4H), 1.77-1.70 (m, 1H), 1.68 (dd, *J* = 3.1, 1.5 Hz, 3H), 1.52 (s, 3H), 1.49-1.39 (m, 1H), 1.33 (s, 3H), 1.18 (s, 3H), 1.13 (d, *J* = 7.0 Hz, 3H), 1.09 (d, *J* = 5.7 Hz, 1H), 0.99 (d, *J* = 6.6 Hz, 3H), 0.93 (t, *J* = 7.4 Hz, 3H), 0.89 (s, 9H), 0.09 (s, 3H), 0.06 (s, 3H).

^{13}C NMR (101 MHz, $CDCl_3$, 39 peaks total) δ 179.0, 167.6, 140.0, 137.4, 134.6, 133.4, 128.8, 127.5, 108.2, 93.5, 81.4, 77.9, 77.7, 74.2, 67.3, 65.8, 56.5, 45.8, 41.3, 39.7, 36.5, 27.0, 26.7, 26.3, 26.3, 26.1 (3 peaks), 24.4, 18.6, 18.5, 16.3, 14.7, 14.6, 13.5, 12.4, 11.8, -5.10, -5.12.

HRMS calculated for $C_{39}H_{61}O_9Si^+$ $[M+H]^+$: 701.4085; found: 701.4082.

FTIR (ATR) 3410 (br), 2956, 2929, 2856, 1714, 1651, 1462, 1381, 1255, 1207, 1190, 1153, 1085, 1065, 1012, 974, 893, 837, 777, 735 cm^{-1}

$[\alpha]^{24}_D$ = 25° (*c* = 0.40, CH_2Cl_2)

^{1}H-NMR (400 MHz, CDCl$_3$)

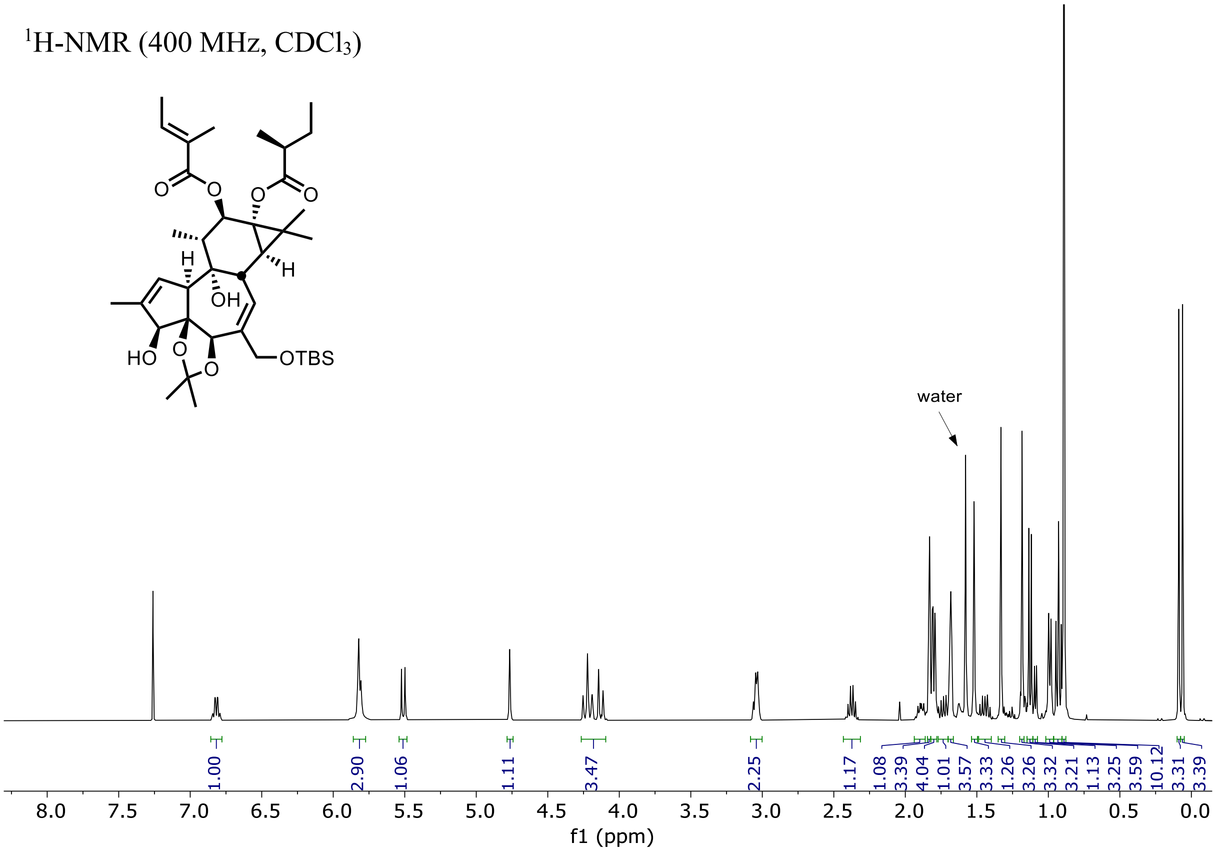

^{13}C-NMR (126 MHz, CDCl$_3$)

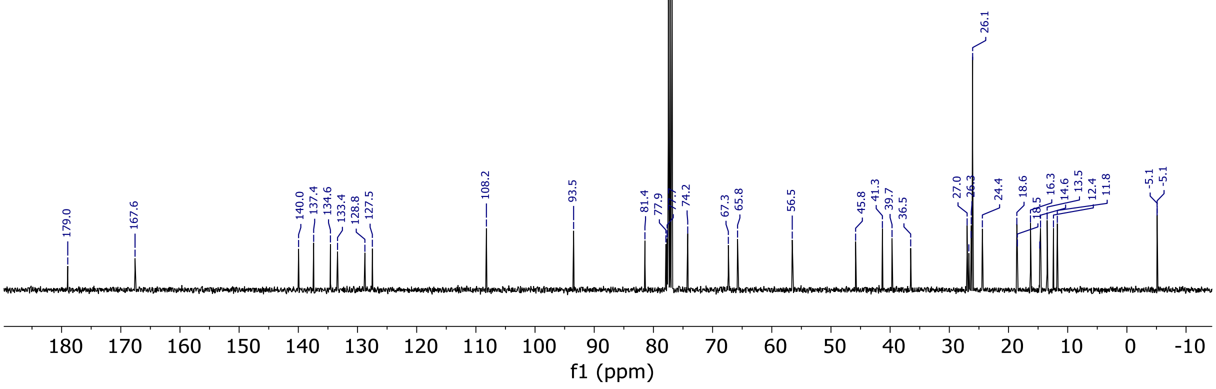

SUW422 preparation (benzoylation and deprotection)

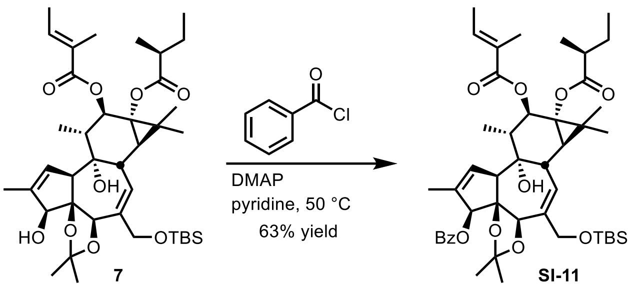

To a flame-dried vial equipped with a stir bar was added **7** (49.1 mg, 69.8 µmol, 1 equiv) followed by distilled pyridine (1.0 mL). Benzoyl chloride (81 µL, 698 µmol, 10 equiv) was added directly as a single portion followed by DMAP (4.3 mg, 35 µmol, 0.5 equiv). The reaction was sealed with a Teflon cap and heated to 50 °C for 3 hours. TLC analysis indicated complete consumption of **7**. The reaction was quenched with sat. $NaHCO_3$ (10 mL) and diluted with brine (10 mL). The aqueous layer was extracted with EtOAc (3x20 mL). The combined organic layers were dried over Na_2SO_4, filtered, concentrated, and azeotroped with heptane (3x50 mL) to remove pyridine. Purification was accomplished by silica gel flash column chromatography (5-15% ether/Hex, 1x10 cm) affording benzoate **SI-11** (35.4 mg, 63%) as a white solid. Compound purity was established by TLC (one spot) analysis.

SI-11 TLC R_f = 0.78 (20% Et_2O/Hex, UV active, blue spot in *p*-anisaldehyde)

^{1}H NMR (400 MHz, $CDCl_3$) δ 8.18-8.14 (m, 2H), 7.60-7.55 (m, 1H), 7.48-7.42 (m, 2H), 6.82 (dq, *J* = 7.0, 1.5 Hz, 1H), 6.00-5.95 (m, 2H), 5.92-5.80 (m, 1H), 5.52 (d, *J* = 9.8 Hz, 1H), 4.76 (d, *J* = 1.5 Hz, 1H), 4.28 (app d, *J* = 12.7 Hz, 1H), 4.21 (app d, *J* = 12.7 Hz, 1H), 3.16 (dt, *J* = 3.7, 2.0 Hz, 1H), 3.04 (app t, *J* = 6.1 Hz, 1H), 2.39 (app sex, *J* = 7.0 Hz, 1H), 2.03-1.94 (m, 1H), 1.86-1.82 (m, 3H), 1.80 (app d, *J* = 7.1, 3H), 1.79-1.71 (m, 1H), 1.64-1.60 (m, 3H), 1.52-1.40 (m, 1H), 1.34 (s, 3H), 1.22 (s, 3H), 1.19 (s, 3H), 1.14 (d, *J* = 7.3 Hz, 3H), 1.11 (d, *J* = 5.6 Hz, 1H), 1.08 (d, *J* = 6.5 Hz, 3H), 0.92 (s, 9H), 0.14 (s, 3H), 0.12 (s, 3H).

^{13}C NMR (126 MHz, $CDCl_3$, 46 peaks total) δ 179.0, 167.6, 166.6, 140.2, 137.4, 134.2, 133.4, 130.8, 130.2 (2 peaks), 130.1, 128.8, 128.7, 128.6 (2 peaks), 108.3, 94.1, 82.0, 78.0, 77.7, 74.3, 67.2, 65.8, 57.2, 45.9, 41.3, 39.5, 36.6, 26.9, 26.7, 26.4, 26.3, 26.2 (3 peaks), 24.4, 18.7, 18.6, 16.3, 15.1, 14.6, 13.6, 12.4, 11.8, -5.0, -5.1.

HRMS calculated for $C_{46}H_{66}NaO_{10}Si^+$ $[M+Na]^+$: 829.4323; found 829.4304.

FTIR (ATR) 3398 (br), 2961, 2925, 2857, 1716, 1458, 1381, 1259, 1068, 837, 700, 669 cm^{-1}

$[\alpha]^{23}_D$ = 20° (*c* = 0.07, CH_2Cl_2)

^{1}H-NMR (400 MHz, $CDCl_3$)

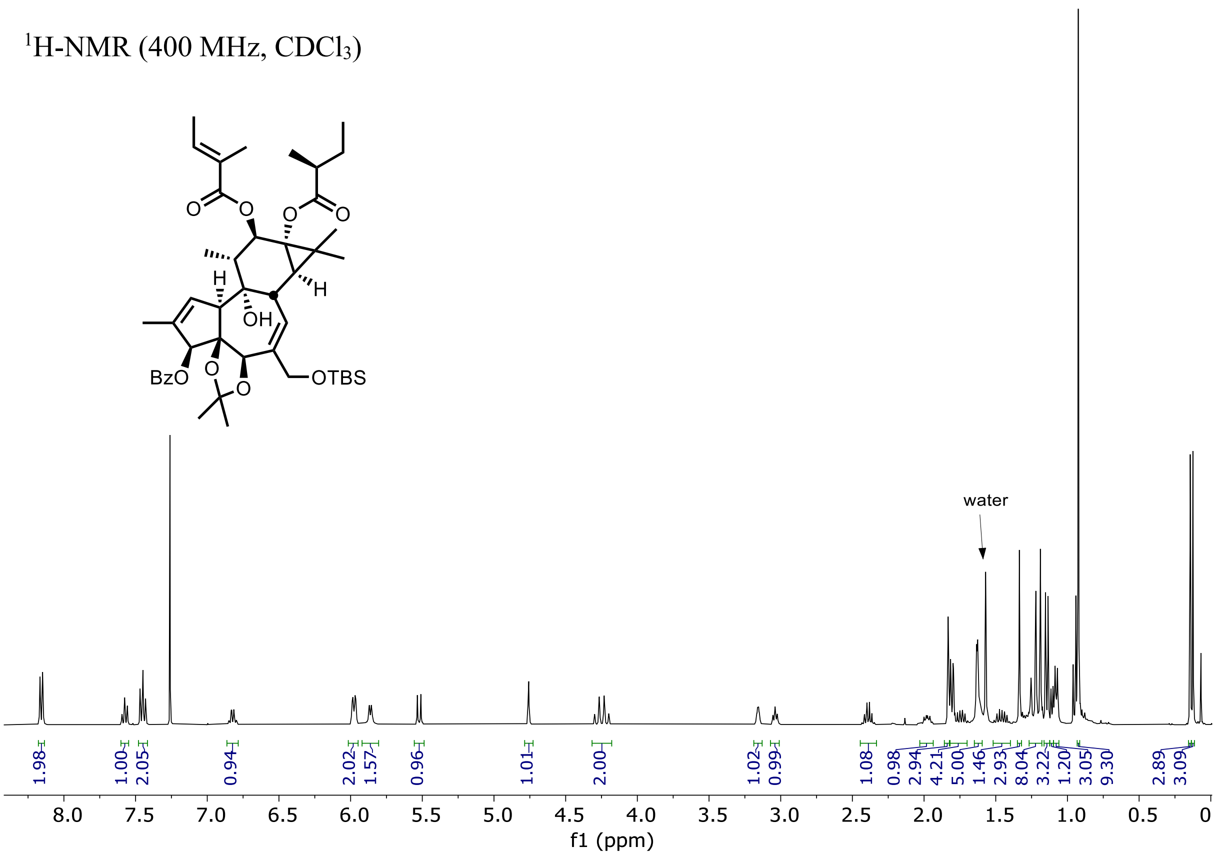

^{13}C-NMR (101 MHz, $CDCl_3$)

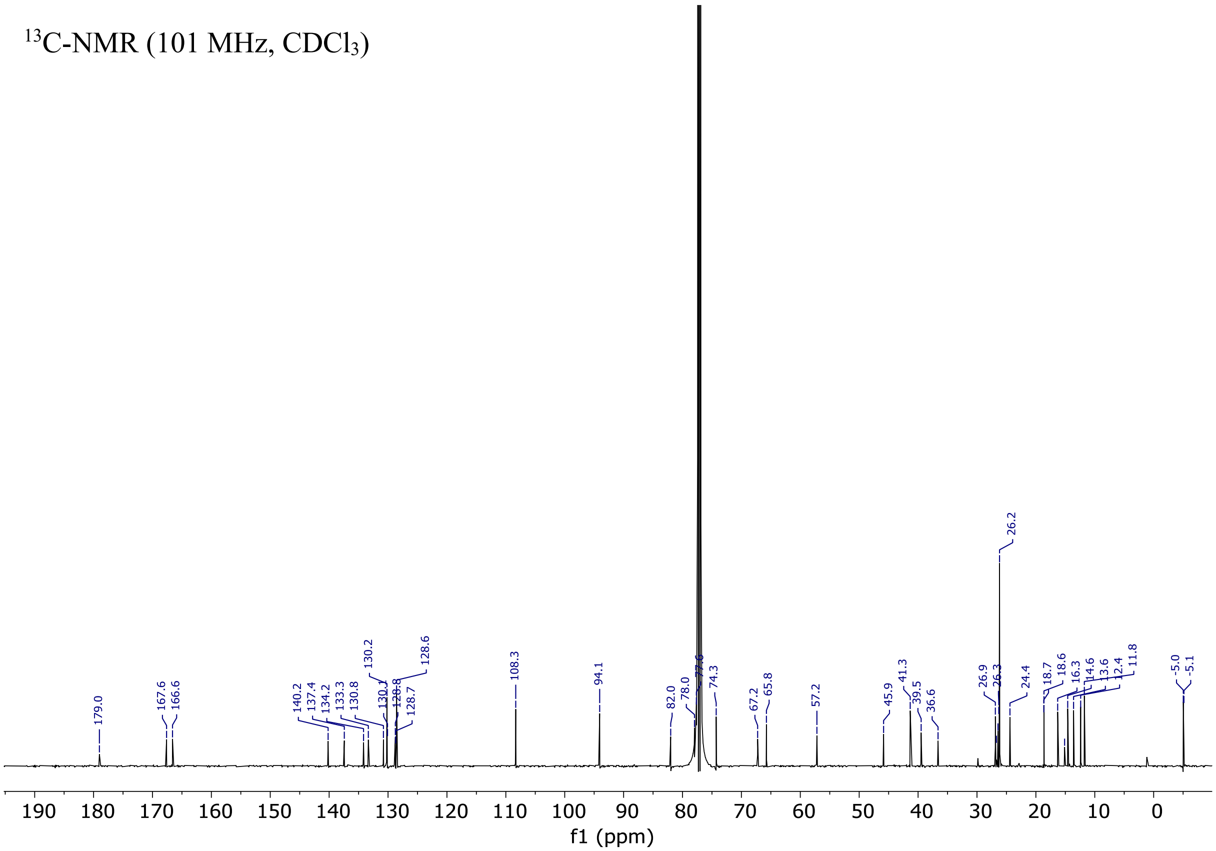

SUW422 deprotection

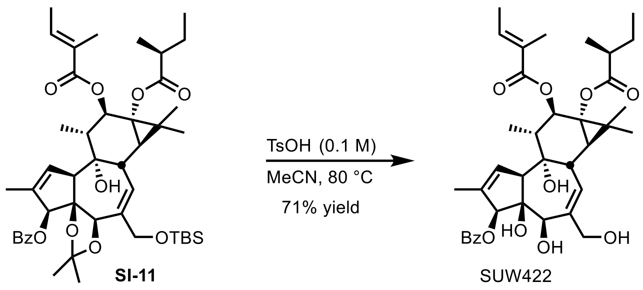

To a vial equipped with a stir bar was added **SI-11** (35.4 mg, 43.9 µmol, 1 equiv) followed by MeCN (1.8 mL). TsOH in water (0.42 M, 0.6 mL) was added (final TsOH concentration of 0.105 M). The reaction mixture was stirred at 80 °C for 30 minutes. TLC analysis indicated complete consumption of **SI-11**. The reaction with quenched with sat. $NaHCO_3$ (5 mL) and diluted with brine (5 mL). The aqueous layer was extracted with EtOAc (3x10 mL). The combined organic layers were dried over Na_2SO_4, filtered, and concentrated. Purification was accomplished by silica gel flash column chromatography (20-60% EtOAc/Hex, 1x10 cm) affording SUW422 (20.4 mg, 71%) as a white solid. Compound purity was established by TLC (one spot) analysis.

SUW422 TLC R_f = 0.26 (50% EtOAc/Hex, UV active, blue spot in *p*-anisaldehyde)

^{1}H NMR (400 MHz, $CDCl_3$) δ 8.13-8.09 (m, 2H), 7.64-7.58 (m, 1H), 7.51-7.44 (m, 2H), 6.81 (dq, *J* = 7.1, 1.5 Hz, 1H), 6.00 (app d, *J* = 1.8 Hz, 1H), 5.73 (app d, *J* = 5.2 Hz, 1H), 5.73 (br s, 1H), 5.46 (d, *J* = 10.1 Hz, 1H), 5.41 (app d, *J* = 1.4 Hz, 1H), 4.57 (s, 1H), 4.28 (d, *J* = 12.1 Hz, 1H), 4.21 (d, *J* = 12.1, 1H), 3.25 (app t, *J* = 5.3 Hz, 1H), 3.25 (br s, 1H) 3.04 (app dt, *J* = 4.0, 2.1 Hz, 1H), 2.37 (app sex, *J* = 6.9 Hz, 1H), 2.20-2.09 (m, 1H), 1.84-1.81 (m, 3H), 1.81-1.76 (m, 6H), 1.76-1.67 (m, 1H), 1.50-1.39 (m, 1H), 1.21 (s, 3H), 1.18 (s, 3H), 1.12 (d, *J* = 7.0 Hz, 3H), 1.04 (d, *J* = 6.5 Hz, 3H), 1.01 (d, *J* = 5.4 Hz, 1H), 0.93 (t, *J* = 7.5 Hz, 3H).

^{13}C NMR (126 MHz, $CDCl_3$, 37 peaks total) δ 179.1, 168.9, 167.7, 139.4, 137.4, 134.0, 133.1, 132.1, 130.3 (2 peaks), 129.9, 129.4, 128.8, 128.7 (2 peaks), 89.3, 80.7, 80.5, 77.8, 68.7, 65.38, 65.36, 57.9, 44.7, 41.3, 38.7, 36.9, 26.3, 26.1, 23.9, 17.1, 16.3, 15.1, 14.5, 14.0, 12.4, 11.8.

HRMS calculated for $C_{37}H_{48}NaO_{10}^+$ $[M+Na]^+$: 675.3098; found 675.3130.

FTIR (ATR) 3419 (br), 2964, 2925, 2878, 1712, 1452, 1377, 1318, 1261, 1154, 1120, 1070, 1026, 976, 934, 881, 804, 712, 669, 652 cm^{-1}

$[\alpha]^{25}_D$ = -32° (*c* = 0.22, CH_2Cl_2)

^{1}H-NMR (400 MHz, $CDCl_3$)

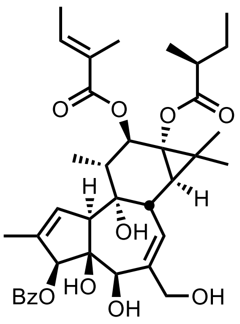

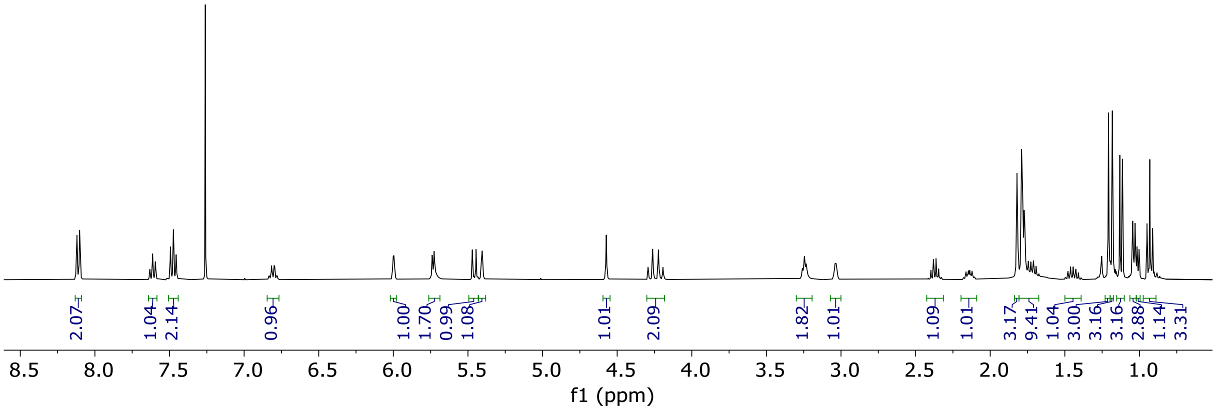

^{13}C-NMR (126 MHz, $CDCl_3$)

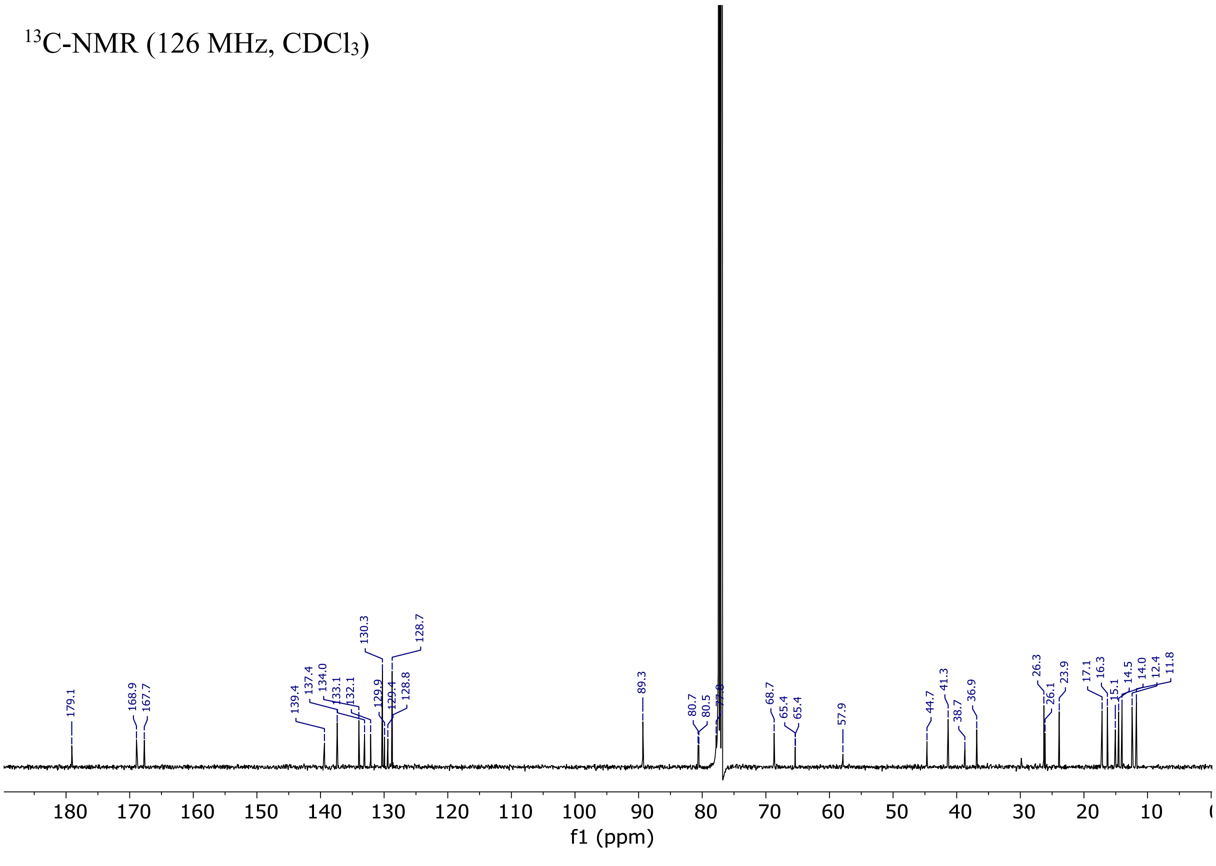

SUW425

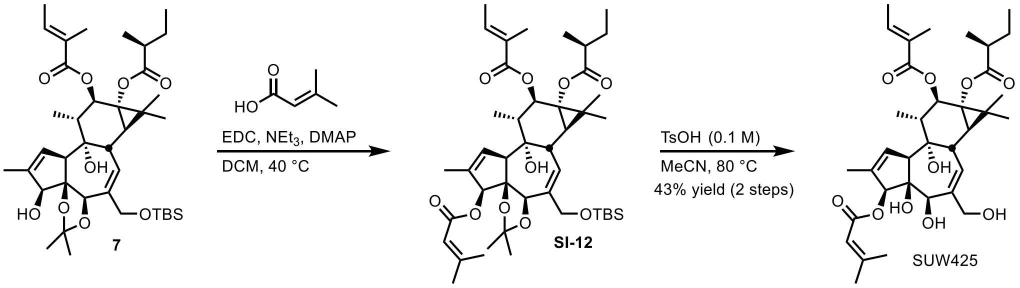

In a vial equipped with a stir bar was added senecioic acid (64 mg, 0.64 mmol, 20 equiv), triethylamine (0.10 mL, 0.72 mmol, 23 equiv) and EDC (130 mg, 0.68 mmol, 20.5 equiv) followed by DCM (1.36 mL). In a separate flame-dried vial equipped with a stir bar was added **7** (23.5 mg, 0.033 mmol, 1 equiv). A portion of the pre-activated acid solution (0.50 mL) was transferred to the vial of **7** in one portion. DMAP (8 mg, 0.064 mmol, 0.5 equiv) was added directly to the reaction mixture as a single portion. The reaction was stirred at 40 ˚C for 5 hr. TLC analysis indicated complete consumption of **7**. The reaction mixture was cooled to rt and quenched with methanol (0.10 mL) and diluted with NH_4Cl (20 mL) and brine (15 mL). The aqueous layers were extracted with EtOAc (10 mL). The combined organic layers were dried over Na_2SO_4, filtered, and concentrated. The crude product was used directly in the next step.

To a vial equipped with a stir bar was added crude **SI-12**. 1 mL of a stock solution of TsOH in 3:1 MeCN: H_2O (0.10 M) was added to the vial directly as a single portion. The reaction mixture was stirred at 80 °C for 30 minutes. TLC analysis indicated complete consumption of **SI-12**. The reaction with quenched with sat. $NaHCO_3$ (10 mL) and diluted with brine (15 mL). The aqueous layer was extracted with EtOAc (25 mL). The combined organic layers were dried over Na_2SO_4, filtered, and concentrated. Purification was accomplished by silica gel flash column chromatography (20-60% EtOAc/Hex, 1x7 cm) affording SUW425 (9 mg, 43% yield over 2 steps) as a white solid. Compound purity was established by TLC (one spot) analysis.

SUW425 TLC R_f = 0.30 (35% EtOAc/Hex, UV active, blue spot in *p*-anisaldehyde)

^{1}H NMR (400 MHz, $CDCl_3$) δ 6.80 (dq, *J* = 7.1, 1.6 Hz, 1H), 5.91 (app d, *J* = 1.8 Hz, 1H), 5.86 (app p, *J* = 1.3 Hz, 1H), 5.71 (d, *J* = 5.2 Hz, 1H), 5.68 (br s, 1H), 5.43 (d, *J* = 10.1 Hz, 1H), 5.22 (app d, *J* = 1.5 Hz, 1H), 4.47 (s, 1H), 4.25 (d, *J* = 12.1 Hz, 1H), 4.17 (d, *J* = 12.1 Hz, 1H), 3.27 (t, *J* = 5.3 Hz, 1H), 3.19 (s, 1H), 3.00-2.94 (m, 1H), 2.37 (app sex, *J* = 6.9 Hz, 1H), 2.20 (d, *J* = 1.3 Hz, 3H), 2.13 (dq, *J* = 10.1, 6.6 Hz, 1H), 1.92 (d, *J* = 4.9 Hz, 3H), 1.83-1.80 (m, 3H), 1.79 (dd, *J* = 7.2, 1.3 Hz, 3H), 1.77-1.70 (m, 1H), 1.69-1.67 (m, 3H), 1.49-1.41 (m, 1H), 1.22 (s, 3H), 1.18 (s, 3H), 1.12 (d, *J* = 7.1 Hz, 3H), 1.02-0.98 (m, 3H), 0.93 (t, *J* = 7.5 Hz, 3H).

^{13}C NMR (126 MHz, $CDCl_3$, 35 peaks total) δ 179.1, 168.8, 167.7, 160.8, 139.3, 137.4, 133.0, 131.6, 130.2, 128.8, 115.3, 87.6, 80.5, 78.0, 77.5, 77.4, 68.8, 65.4, 57.8, 44.7, 41.3, 38.7, 36.9, 27.9, 26.3, 26.1, 23.9, 20.8, 17.1, 16.3, 15.2, 14.5, 13.8, 12.4, 11.8.

HRMS calculated for $C_{35}H_{51}O_{10}^+$ $[M+H]^+$: 631.3482; found 631.3472.

FTIR (ATR) 3415 (br), 2964, 2925, 2878, 1712, 1649, 1456, 1379, 1257, 1146, 1074, 975, 883, 803, 734, 669, 651 cm^{-1}

$[\alpha]^{25}_{D}$ = -84° (*c* = 0.36, CH_2Cl_2)

^{1}H-NMR (400 MHz, $CDCl_3$)

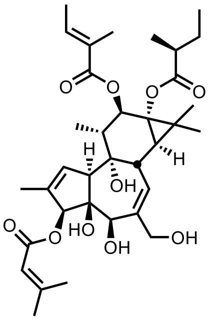

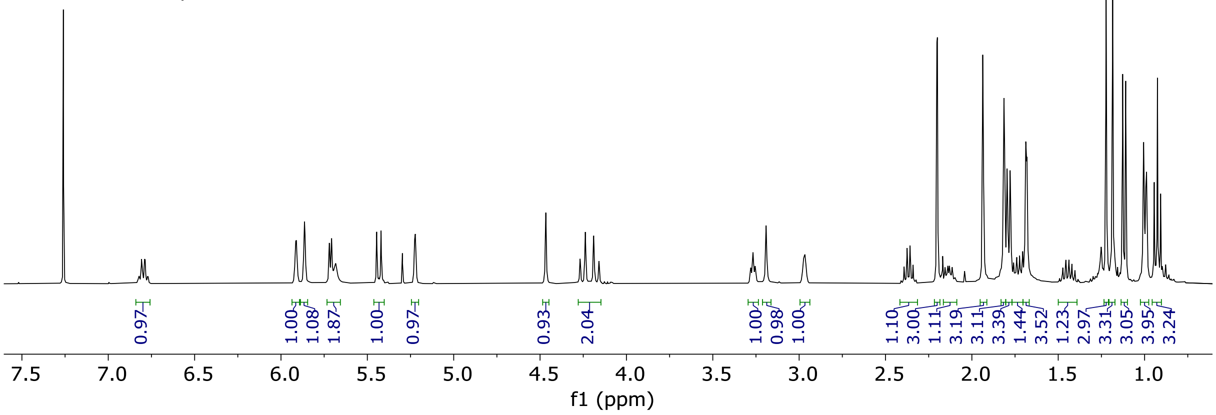

^{13}C-NMR (126 MHz, $CDCl_3$)

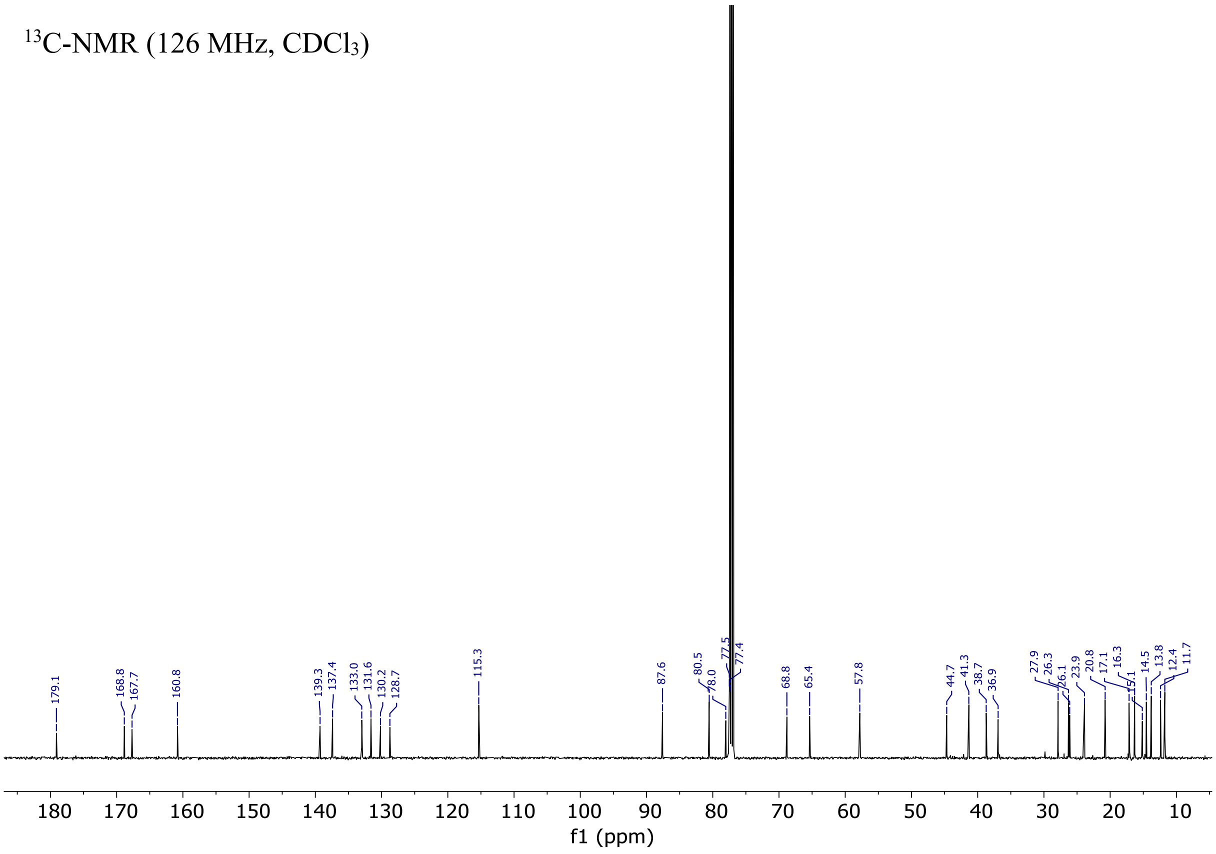

SUW427 preparation

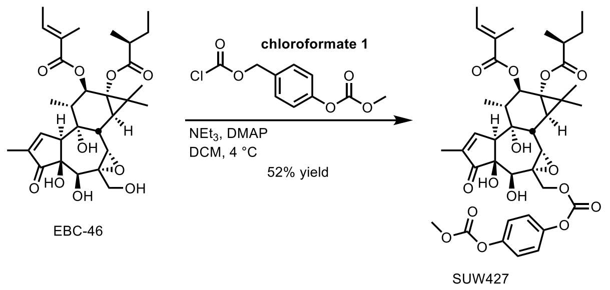

To a flame-dried vial equipped with a stir bar was added EBC-46 (3.0 mg, 5.3 µmol, 1 equiv). DMAP (0.6 mg, 5.3 µmol, 1 equiv) and triethylamine (0.5 mg, 5.3 µmol, 1 equiv) were added directly as a single portion. Chloroformate 1 (0.6 mg, 2.6 µmol, 0.5 equiv) was added as a single portion from a freshly prepared stock solution. The reaction was stirred at 4 °C for 2 hr at which point another 0.5 equiv chloroformate was added. The reaction was stirred at 4 °C for 16 hr. TLC analysis indicated complete consumption of EBC-46. The reaction was quenched with MeOH (50 µL) and diluted with NH_4Cl (15 mL) and brine (15 mL). The aqueous layer was extracted with EtOAc (2x30 mL). The combined organic layers were dried over Na_2SO_4, filtered, and concentrated. Purification was accomplished by silica gel flash column chromatography (10-30% EtOAc/Hex, 1x10 cm) affording SUW427 as a colorless oil (2.1 mg, 52% yield). Compound purity was established by TLC (one spot) analysis.

SUW427 TLC R_f = 0.67 (50% EtOAc/Hex, UV active, dark blue spot in *p*-anisaldehyde)

^{1}H NMR (400 MHz, $CDCl_3$) δ 7.71 (dd, *J* = 2.6, 1.5 Hz, 1H), 7.44-7.39 (m, 2H), 7.21-7.16 (m, 2H), 6.86-6.78 (m, 1H), 5.44 (d, *J* = 9.9 Hz, 1H), 5.17 (d, *J* = 12.1 Hz, 1H), 5.13 (d, *J* = 12.1 Hz, 1H), 4.84 (d, *J* = 11.8 Hz, 1H), 4.27 (s, 1H), 4.08 (app t, *J* = 2.8 Hz, 1H), 4.03 (d, *J* = 11.9 Hz, 1H), 3.90 (s, 3H), 3.18 (d, *J* = 6.0 Hz, 1H), 3.18 (s, 1H), 2.39 (app sex, *J* = 7.0 Hz, 1H), 2.02-1.92 (m, 1H), 1.82 (app t, *J* = 1.4 Hz, 3H), 1.79 (app d, *J* = 7.1, 3H), 1.77-1.68 (m, 4H), 1.51-1.41 (m, 1H), 1.26 (s, 3H), 1.24 (s, 3H), 1.14 (d, *J* = 7.0 Hz, 3H), 0.94 (t, *J* = 7.4 Hz, 3H), 0.86 (d, *J* = 6.5 Hz, 3H).

^{13}C NMR (126 MHz, $CDCl_3$, 40 peaks total) δ 209.7, 179.1, 167.6, 164.6, 155.0, 154.2, 151.4, 137.8, 133.6, 133.1, 129.9 (2 peaks), 128.6, 121.4 (2 peaks), 77.4, 76.8, 72.4, 69.9, 69.3, 68.8, 65.6, 65.0, 60.3, 55.6, 48.9, 46.0, 41.3, 36.2, 36.0, 26.8, 26.3, 23.8, 17.4, 16.3, 15.2, 14.6, 12.4, 11.8, 9.9.

HRMS calculated for $C_{40}H_{51}O_{15}^+$ $[M+H]^+$: 771.3223; found 771.3214.

FTIR (ATR) 3388 (br), 2960, 2922, 2852, 1765, 1712, 1259, 1084, 1018, 798 cm^{-1}

$[\alpha]^{21}_D$ = -30° (*c* = 0.03, CH_2Cl_2)

^{1}H-NMR (400 MHz, $CDCl_3$)

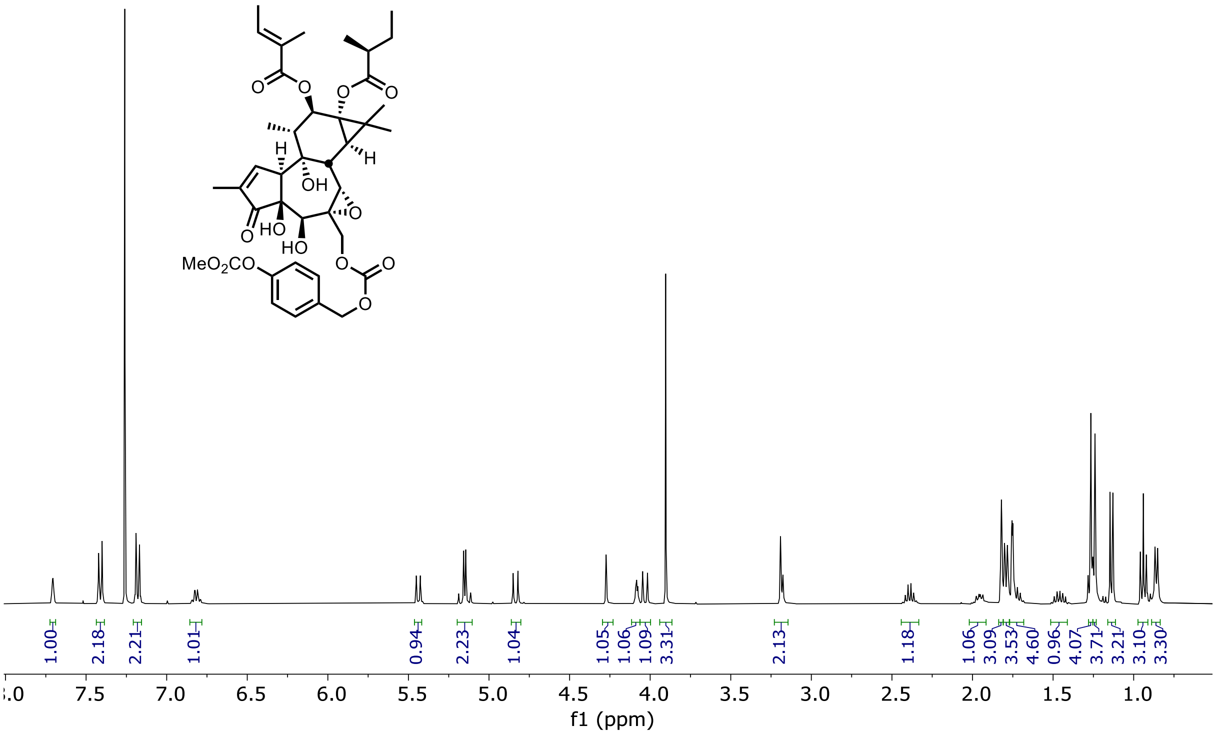

^{13}C-NMR (126 MHz, $CDCl_3$)

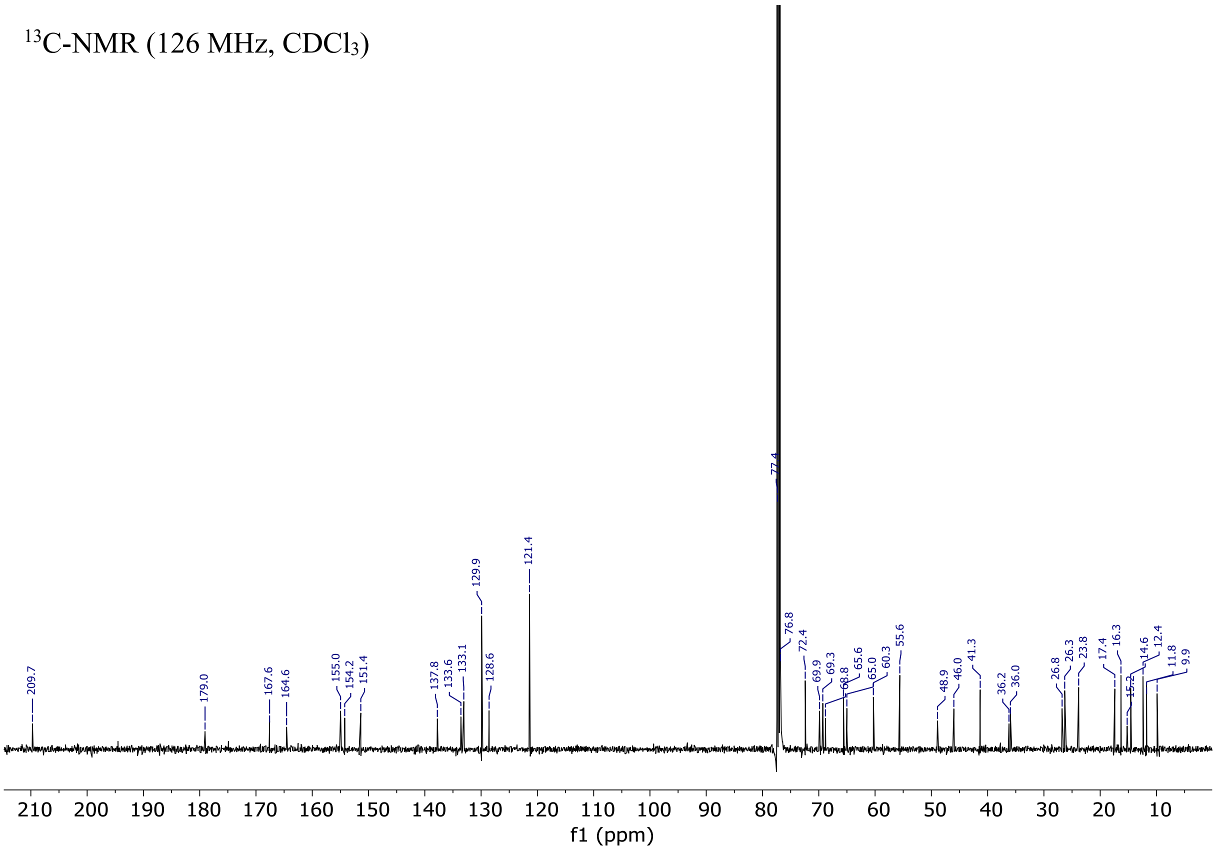

SUW428 preparation

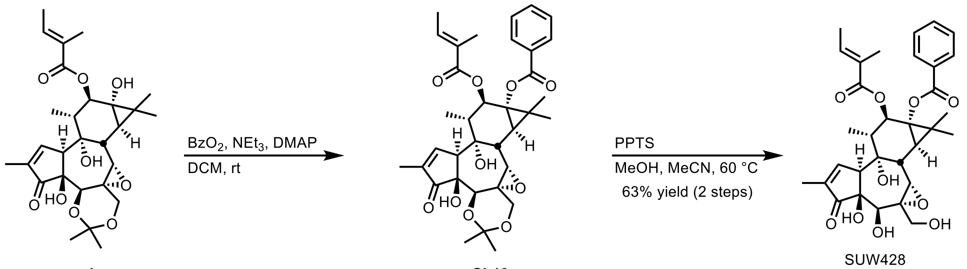

4 SI-13 SUW428

To a flame-dried vial equipped with a stir bar was added **4** (22 mg, 0.042 mmol, 1 equiv) followed by DCM (0.75 mL). Triethylamine (20 µL, 0.142 mmol, 3.5 equiv) was added followed by benzoic anhydride (25 mg, 0.142 mmol, 3.5 equiv) and DMAP (13 mg, 0.155 mmol, 3.9 equiv). The reaction was stirred at rt for 2 hr. TLC analysis indicated complete consumption of **4**. The reaction was quenched MeOH (0.5 mL). The reaction diluted with NH_4Cl (25 mL) and water (25 mL). The aqueous layer was extracted with EtOAc (2x50 mL). The combined organic layers were dried over Na_2SO_4, filtered, and concentrated. The resulting intermediate **SI-13** was used directly in the next step.

In a vial equipped with a stir bar, crude **SI-13** was dissolved in a PPTS MeOH:MeCN stock solution (64 mg PPTS in 3 mL 5:1 MeOH:MeCN). The reaction was stirred at 60 °C for 6 hr. TLC analysis indicated complete consumption of intermediate **SI-13**. The reaction was diluted with brine (25 mL). The aqueous layer was extracted with EtOAc (25 mL). The organic layer was dried over Na_2SO_4, filtered, and concentrated. Purification was accomplished by silica gel flash column chromatography (20-70% EtOAc/Hex, 1x7 cm) affording SUW428 as a white solid (15.6 mg, 63% yield over 2 steps). Compound purity was established by TLC (one spot) analysis.

SI-13 TLC R_f = 0.86 (80% EtOAc/Hex, UV active, green spot in *p*-anisaldehyde)
SUW428 TLC R_f = 0.43 (80% EtOAc/Hex, UV active, dark blue spot in *p*-anisaldehyde)

^{1}H NMR (600 MHz, $CDCl_3$) δ 8.02 (d, *J* = 7.8, 2H), 7.77 (app s, 1H), 7.58 (t, *J* = 7.4 Hz, 1H), 7.44 (t, *J* = 7.7 Hz, 2H), 6.85-6.80 (m, 1H), 5.61 (d, *J* = 9.8 Hz, 1H), 4.26 (s, 1H), 4.13 (app p, *J* = 2.7 Hz, 1H), 3.90 (d, *J* = 12.6 Hz, 1H), 3.81 (d, *J* = 12.5 Hz, 1H), 3.32 (s, 1H), 3.26 (d, *J* = 6.7 Hz, 1H), 2.08-2.01 (m, 1H), 1.82 (app s, 3H), 1.80 (app d, *J* = 7.1 Hz, 3H), 1.78 (app d, *J* = 2.0 Hz, 3H), 1.48 (d, *J* = 6.7 Hz, 1H), 1.36 (s, 3H), 1.34 (s, 3H), 0.92 (d, *J* = 6.5 Hz, 3H).

^{13}C NMR (126 MHz, $CDCl_3$, 32 peaks total) δ 210.1, 168.6, 167.6, 164.9, 137.9, 133.9, 133.7, 130.2 (2 peaks), 129.5, 128.7 (2 peaks), 128.6, 77.4, 77.0, 72.5, 71.8, 66.5, 65.4, 64.7, 61.8, 49.1, 46.2, 36.4, 36.3, 27.2, 23.9, 17.4, 15.4, 14.6, 12.4, 9.9.

HRMS calculated for $C_{32}H_{38}O_{10}^+$ $[M+H]^+$: 583.2538; found 583.2530.

FTIR (ATR) 3404 (br), 2976, 2927, 2866, 1699, 1452, 1379, 1325, 1284, 1250, 1117, 1070, 1024, 714 cm^{-1}

$[\alpha]^{23}_D$ = -62° (*c* = 0.20, CH_2Cl_2)

^{1}H-NMR (400 MHz, $CDCl_3$)

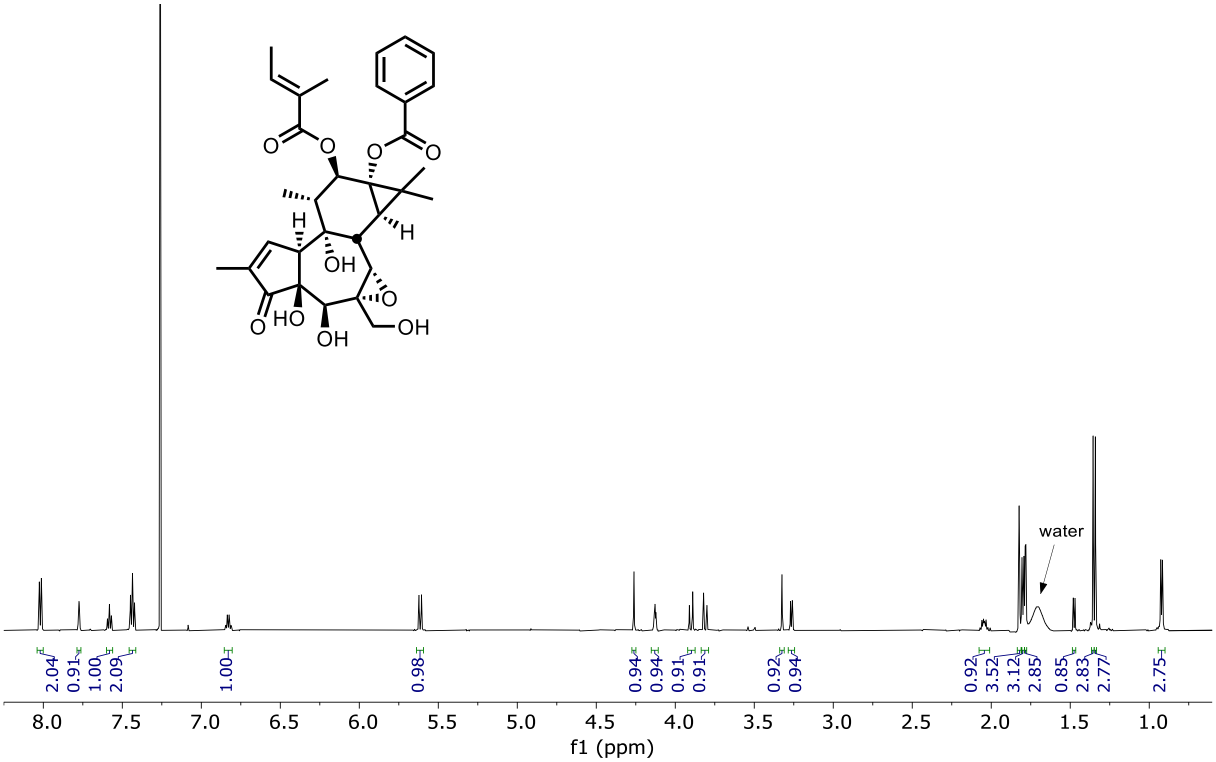

^{13}C-NMR (126 MHz, $CDCl_3$)

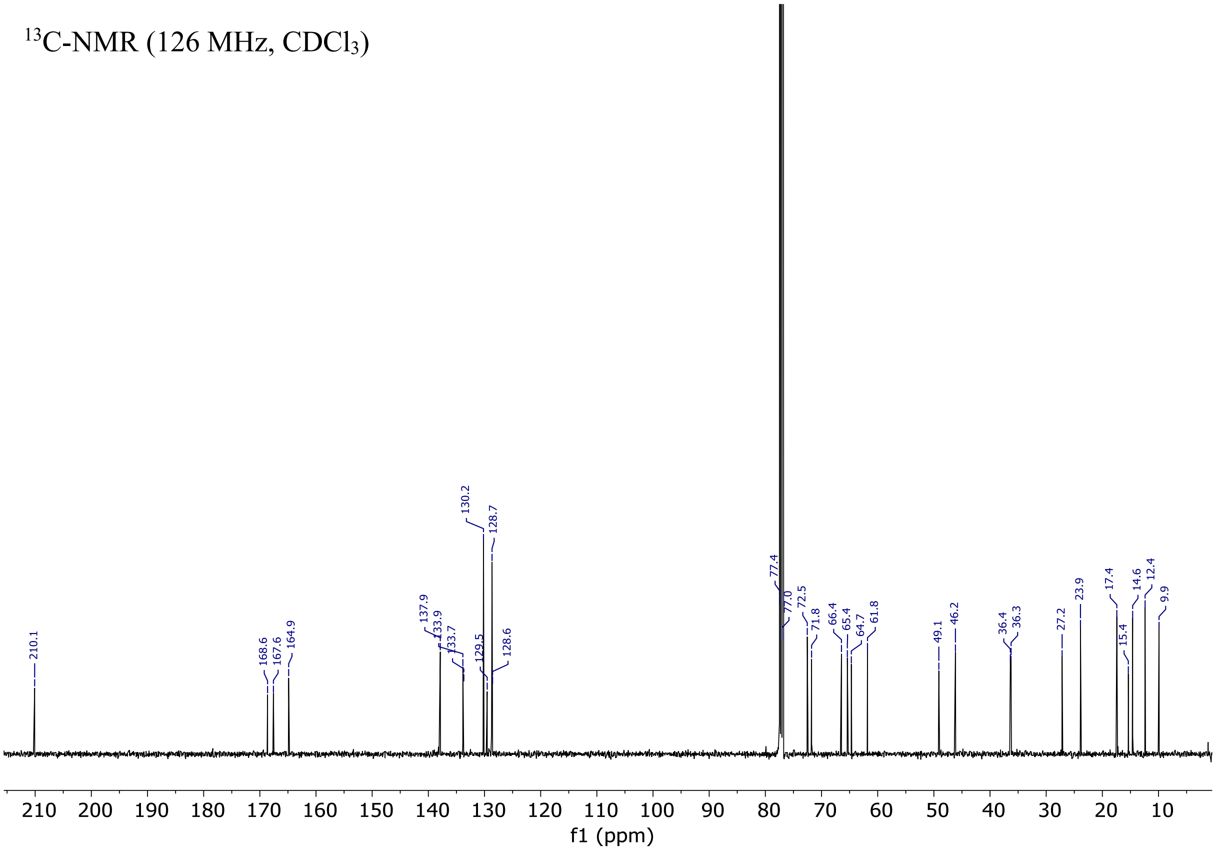

SUW430 preparation (tertbutylation)

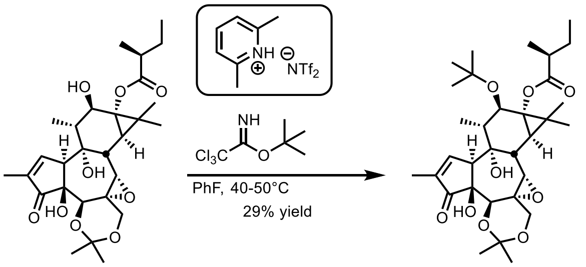

2 SI-14

To a flame dried vial equipped with a stir bar was added **2** (25 mg, 0.048 mmol, 1 equiv) followed by fluorobenzene (0.15 mL). The acid catalyst (lutidinium bistriflimide) was prepared according to the literature (61) as a solution in lutidine. The acid catalyst stock solution (0.18 mL, 0.15 M, 0.026 mmol, 0.5 equiv) was added directly as a single portion followed by tert-butyl 2,2,2-trichloro acetimidate (0.25 mL, 1.221 g/mL, 11.6 equiv). The headspace of the reaction vessel was displaced with argon and the reaction was stirred at 40 °C for 24 hr. TLC analysis indicated partial consumption of **2** and formation of a nonpolar product. The reaction was then stirred at 50 °C for an additional 48 hr. TLC analysis indicated more formation of a nonpolar product. The reaction mixture was filtered through a silica plug (0.5 cm x 4.0 cm). The plug was washed with DCM (2x10 mL) followed by EtOAc (2x5 mL). Purification was accomplished by silica gel flash column chromatography (10-40% Et_2O/Hex, 1x7 cm) affording **SI-14** as a white solid (8.0 mg, 29% yield). Compound purity was established by TLC (one spot) analysis.

SI-14 TLC R_f = 0.21 (40% Et_2O/Hex, UV active, blue spot in *p*-anisaldehyde)

^{1}H NMR (400 MHz, $CDCl_3$) δ 7.64 (app s, 1H), 6.10 (s, 1H), 4.13 (s, 1H), 3.99 (d, *J* = 12.8 Hz, 1H), 3.99 (app s, 1H), 3.80 (d, *J* = 8.7 Hz, 1H), 3.58 (d, *J* = 12.8 Hz, 1H), 3.30 (s, 1H), 3.11 (d, *J* = 7.2 Hz, 1H), 3.00 (s, 1H), 2.44 (app sex, *J* = 6.9 Hz, 1H), 1.83-1.68 (m, 5H), 1.51 (s, 3H), 1.48 (s, 3H), 1.29 (s, 6H), 1.21 (s, 9H), 1.17 (d, *J* = 7.2 Hz, 1H), 0.99 (d, *J* = 6.8 Hz, 3H), 0.96 (t, *J* = 7.4 Hz, 3H).

^{13}C NMR (126 MHz, $CDCl_3$, 32 peaks total) δ 206.5, 178.6, 162.1, 133.7, 101.2, 76.8, 76.5, 73.6, 72.8, 68.8, 67.1, 65.9, 65.5, 60.5, 49.4, 48.4, 41.8, 36.3, 35.3, 28.7 (3 peaks), 26.7, 26.3, 24.9, 23.9, 22.4, 18.0, 16.8, 16.1, 12.0, 10.1.

HRMS calculated for $C_{32}H_{49}O_9^+$ $[M+H]^+$: 577.3371; found 577.3365.

FTIR (ATR) 3348 (br), 2962, 2924, 2854, 1714, 1462, 1377, 1088, 1055, 827, 766, 669 cm^{-1}

$[\alpha]^{23}_D$ = 26° (*c* = 0.10, CH_2Cl_2)

^{1}H-NMR (400 MHz, $CDCl_3$)

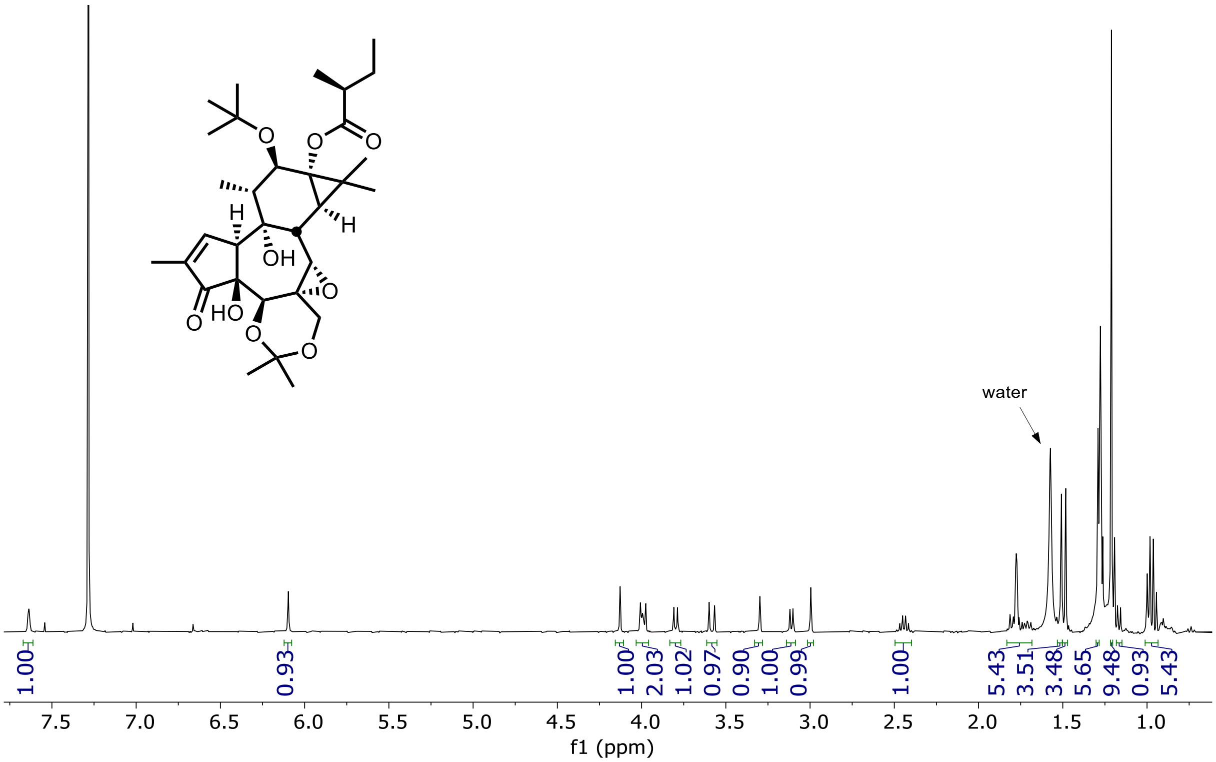

^{13}C-NMR (126 MHz, $CDCl_3$)

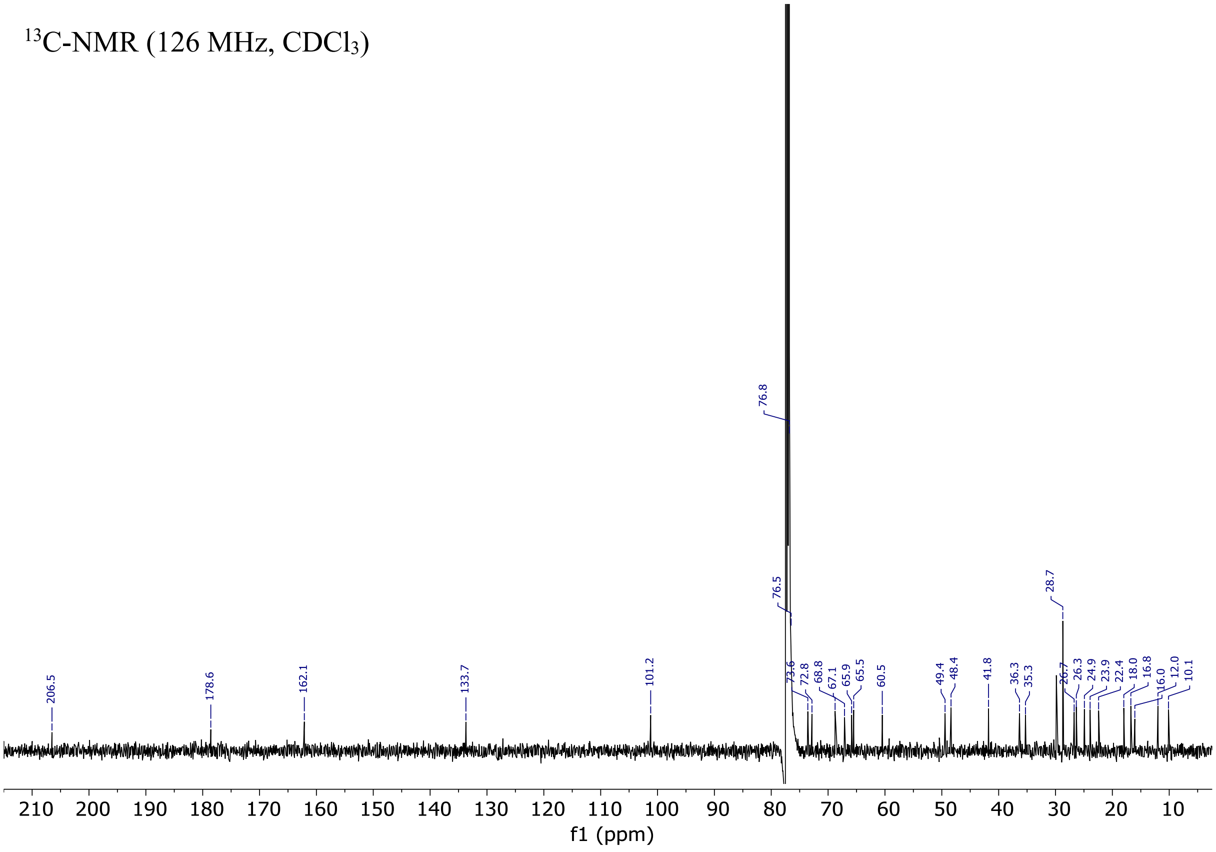

SUW430 preparation (deprotection)

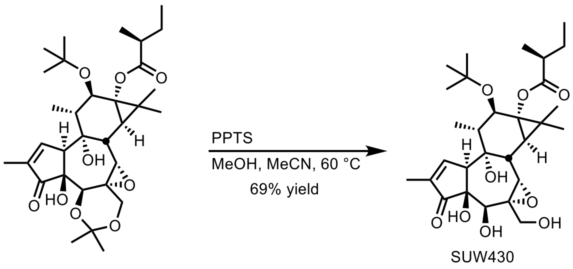

In a vial equipped with a stir bar, **SI-14** (7.3 mg, 0.013 mmol, 1 equiv) was dissolved in 5:1 MeOH:MeCN (0.5 mL). PPTS (37 mg, 0.15 mmol, 12 equiv) was added directly as a single portion. The reaction mixture was stirred at 60 °C for 20 hr. TLC analysis indicated complete consumption of **SI-14**. The reaction was quenched with $NaHCO_3$ (10 mL) and diluted with brine (10 mL). The aqueous layer was extracted with EtOAc (2x10 mL). The combined organic layers were dried over Na_2SO_4, filtered, and concentrated. Purification was accomplished by silica gel flash column chromatography (20-60% EtOAc/Hex, 1x7 cm) affording SUW430 as a white solid (4.7 mg, 69% yield). Compound purity was established by TLC (one spot) analysis.

SUW430 TLC R_f = 0.18 (75% EtOAc/Hex, UV active, blue spot in *p*-anisaldehyde)

^{1}H NMR (600 MHz, $CDCl_3$) δ 7.76 (dd, *J* = 2.6, 1.4 Hz, 1H), 6.13 (s, 1H), 4.21 (s, 1H), 4.01 (app t, *J* = 2.8 Hz, 1H), 3.88 (d, *J* = 12.4 Hz, 1H), 3.79 (d, *J* = 8.8 Hz, 1H), 3.78 (d, *J* = 12.4 Hz, 1H), 3.26 (s, 1H), 3.11 (d, *J* = 7.2 Hz, 1H), 2.43 (app sex, *J* = 7.1 Hz, 1H), 1.76 (dd, *J* = 3.8, 1.9 Hz, 3H), 1.75-1.68 (m, 1H), 1.52-1.44 (m, 1H), 1.25 (s, 3H), 1.24 (s, 3H), 1.19 (s, 9H), 1.16 (d, *J* = 7.2 Hz, 1H), 1.00 (d, *J* = 6.6 Hz, 3H), 0.95 (td *J* = 7.4, 3H).

^{13}C NMR (126 MHz, $CDCl_3$, 29 peaks total) δ 210.6, 178.9, 165.7, 133.5, 77.3, 76.6, 73.8, 72.7, 72.2, 67.2, 65.8, 65.0, 61.9, 49.5, 48.6, 42.0, 36.3, 35.8, 28.9 (3 peaks), 27.0, 26.5, 24.1, 18.1, 16.9, 16.4, 12.2, 10.1.

HRMS calculated for $C_{29}H_{45}O_9^+$ $[M+H]^+$: 537.3058; found 537.3050.

FTIR (ATR) 3398 (br), 2958, 2920, 2850, 1716, 1464, 1086, 1020, 800, 669 cm^{-1}

$[\alpha]^{23}_D$ = 19° (*c* = 0.10, CH_2Cl_2)

^{1}H-NMR (600 MHz, $CDCl_3$)

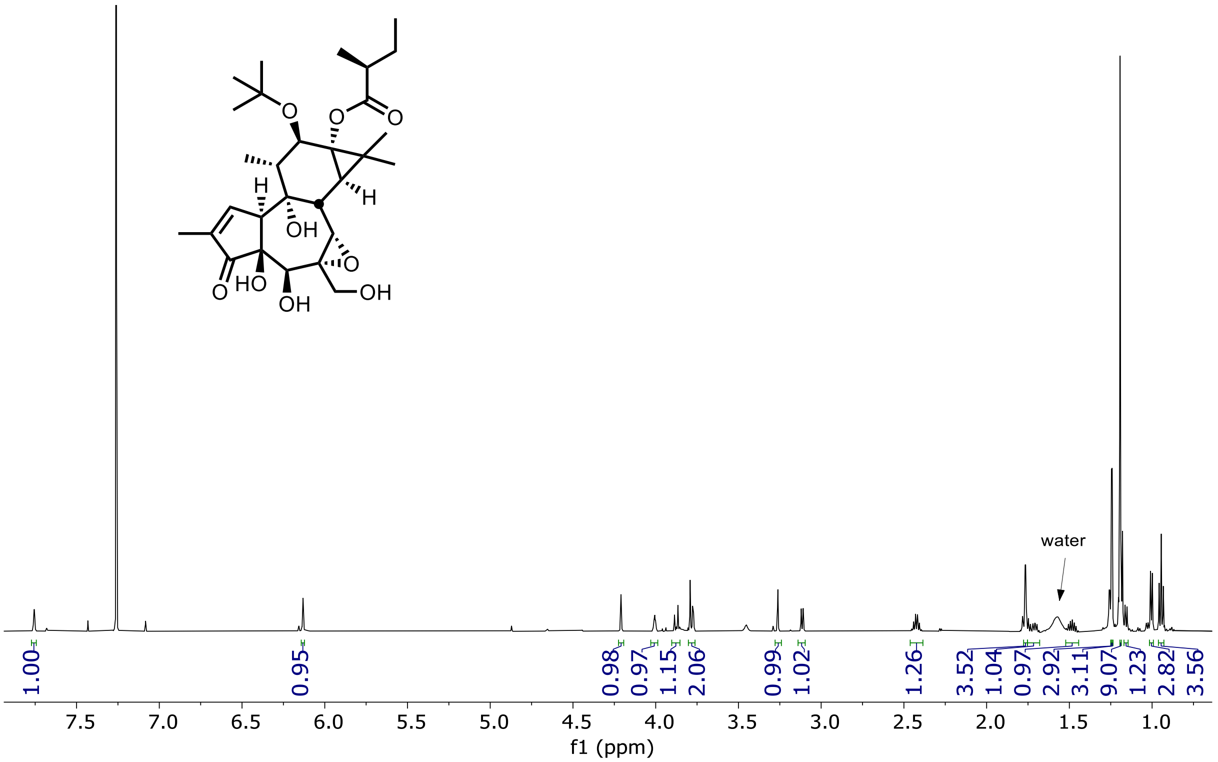

^{13}C-NMR (126 MHz, $CDCl_3$)

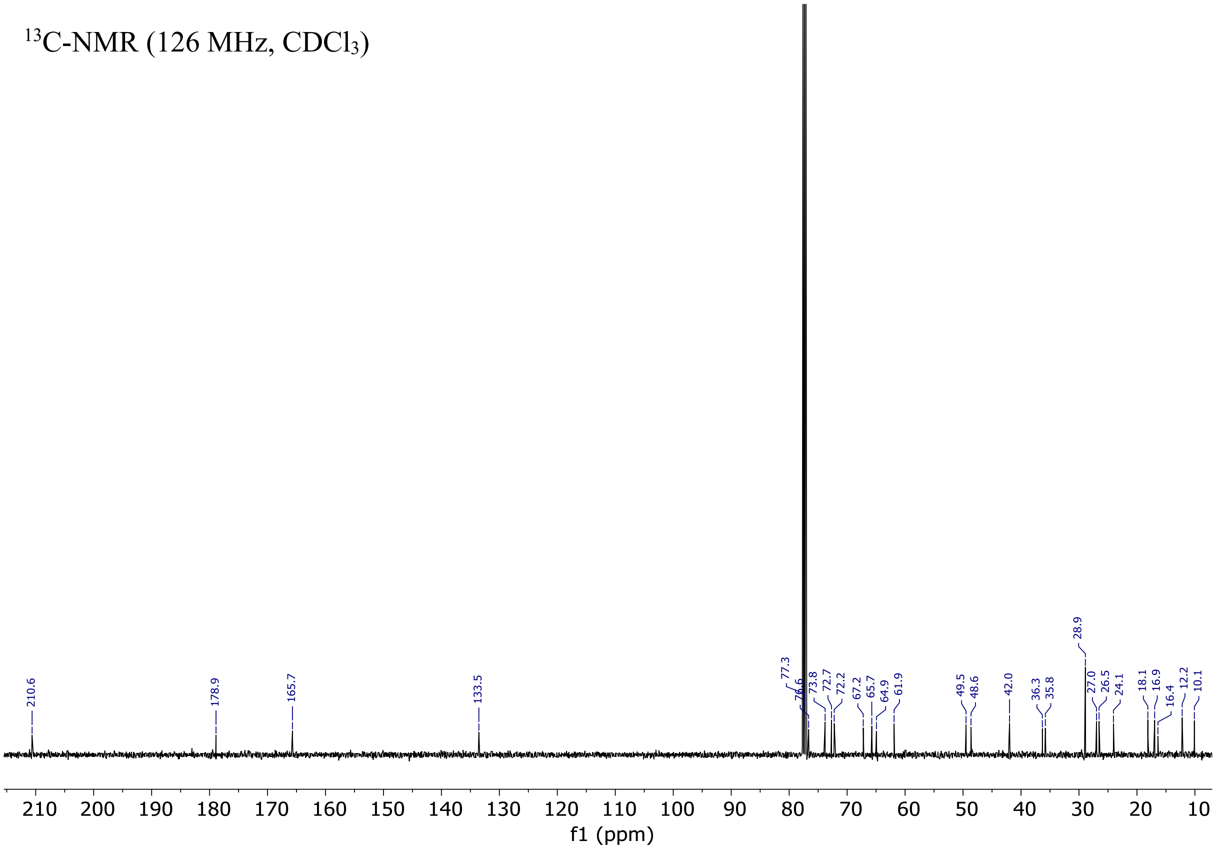

SUW431

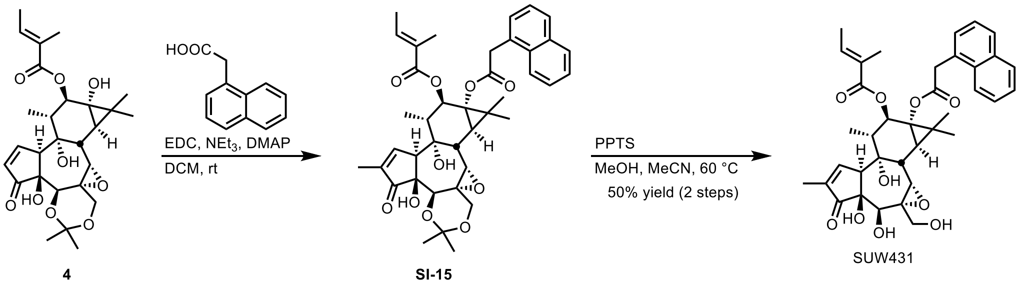

To a flame-dried vial equipped with a stir bar was added 2-(napthalen-1-yl) acetic acid (16 mg, 0.085 mmol, 2 equiv) followed by DCM (0.33 mL), triethylamine (12 µL, 0.089 mmol, 2.1 equiv), and EDC (16 mg, 0.085 mmol, 2 equiv). The acid mixture was stirred for 5 min. at rt. To a separate flame-dried vial equipped with a stir bar was added **4** (22 mg, 0.042 mmol, 1 equiv). The solution of pre-activated acid was transferred to the vial of **4** in one portion. DMAP (6 mg, 0.049 mmol, 0.5 equiv) was added directly to the reaction mixture as a single portion. The reaction was stirred for 90 min. at rt. TLC analysis indicated complete consumption of **4**. The reaction mixture was quenched with NH_4Cl (25 mL). The aqueous layer was extracted with EtOAc (2x25 mL). The combined organic layers were dried over Na_2SO_4, filtered, and concentrated. The resulting intermediate **SI-15** was used directly in the next step.

In a vial equipped with a stir bar, crude **SI-15** was dissolved in a PPTS MeOH:MeCN stock solution (66 mg PPTS in 3 mL 5:1 MeOH:MeCN). The reaction was stirred at 60 °C for 6 hr. TLC analysis indicated complete consumption of intermediate **SI-15**. The reaction was diluted with brine (25 mL). The aqueous layer was extracted with EtOAc (25 mL). The organic layer was dried over Na_2SO_4, filtered, and concentrated. Purification was accomplished by silica gel flash column chromatography (10-33% EtOAc/Hex, 1x7 cm) affording SUW431 as a white solid (13.8 mg, 50% yield over 2 steps). Compound purity was established by TLC (one spot) analysis.

SI-15 TLC R_f = 0.72 (50% EtOAc/Hex, UV active, blue spot in *p*-anisaldehyde)
SUW431 TLC R_f = 0.20 (66% EtOAc/Hex, UV active, blue spot in *p*-anisaldehyde)

^{1}H NMR (400 MHz, $CDCl_3$) δ 8.01-7.96 (m, 1H), 7.87-7.83 (m, 1H), 7.81-7.76 (m, 1H), 7.70 (app s, 1H), 7.56-7.51 (m, 1H), 7.51-7.46 (m, 1H), 7.42-7.39 (m, 2H), 6.88-6.80 (m, 1H), 5.51 (d, *J* = 10.0 Hz, 1H), 4.19 (s, 1H), 4.14 (d, *J* = 5.2 Hz, 1H), 4.14 (br s, 1H), 4.02 (app p, *J* = 2.8 Hz, 1H), 3.84 (d, *J* = 12.5 Hz, 1H), 3.75 (d, *J* = 12.5 Hz, 1H), 3.18 (s, 1H), 3.12 (d, *J* = 6.6 Hz, 1H), 1.99-1.91 (m, 1H), 1.88-1.84 (m, 3H), 1.82 (dd, *J* = 7.1, 1.2 Hz, 3H), 1.75 (dd, *J* = 2.9, 1.3 Hz, 3H), 1.17 (s, 3H), 0.87 (d, *J* = 6.5 Hz, 3H), 0.80 (s, 3H).

^{13}C NMR (101 MHz, $CDCl_3$, 37 peaks total) δ 210.0, 174.1, 167.8, 164.8, 137.9, 133.9, 133.6, 132.2, 129.8, 128.8, 128.5, 128.4, 128.3, 126.6, 125.9, 125.5, 124.1, 77.3, 76.6, 72.5, 71.5, 66.4, 65.2, 64.6, 61.8, 49.0, 45.8, 39.2, 36.0, 35.9, 26.6, 23.2, 17.1, 15.2, 14.6, 12.4, 9.9.

HRMS calculated for $C_{37}H_{43}O_{10}^+$ $[M+H]^+$: 647.2851; found 647.2838.

FTIR (ATR) 3421 (br), 2959, 2924, 2855, 1709, 1456, 1259, 1155, 1074, 1024, 785, 669 cm^{-1}

$[\alpha]^{24}_{D}$ = -17° (*c* = 0.50, CH_2Cl_2)

^{1}H-NMR (400 MHz, $CDCl_3$)

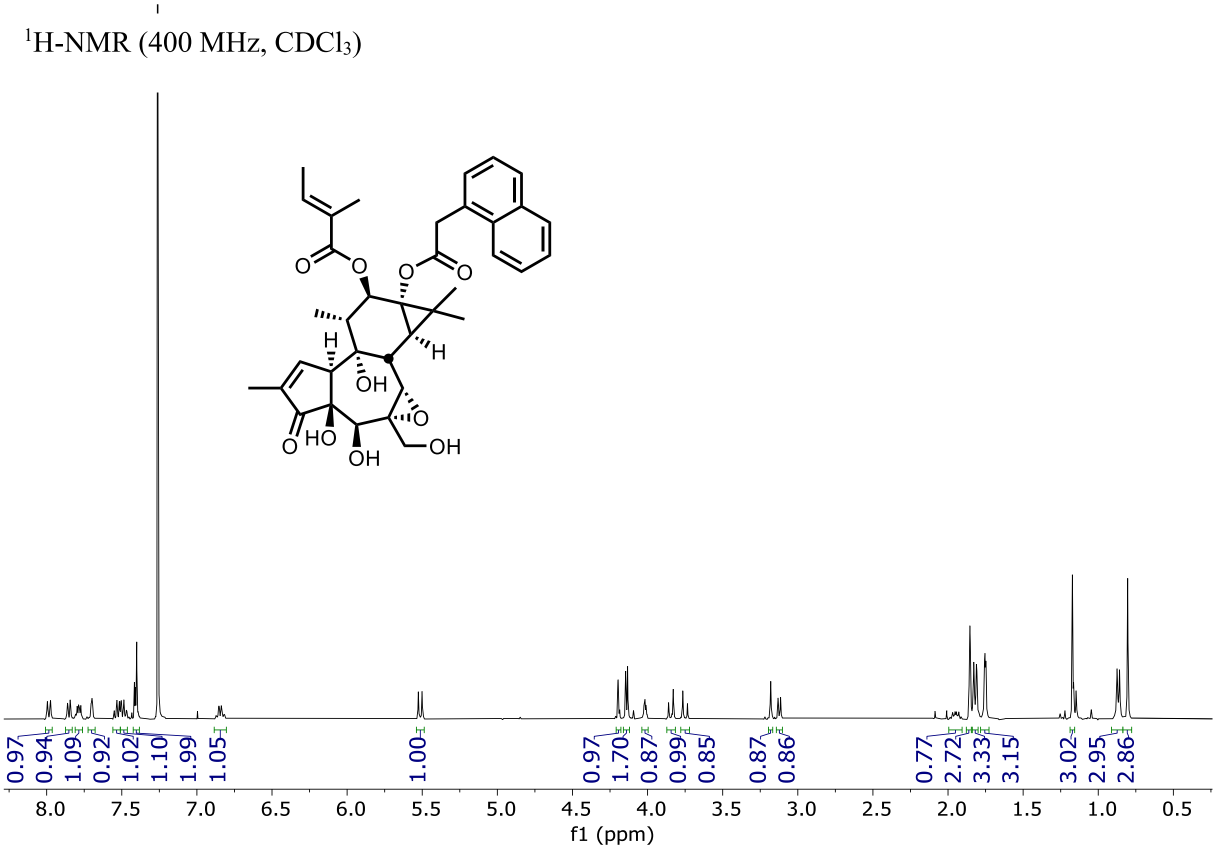

^{13}C-NMR (126 MHz, $CDCl_3$)

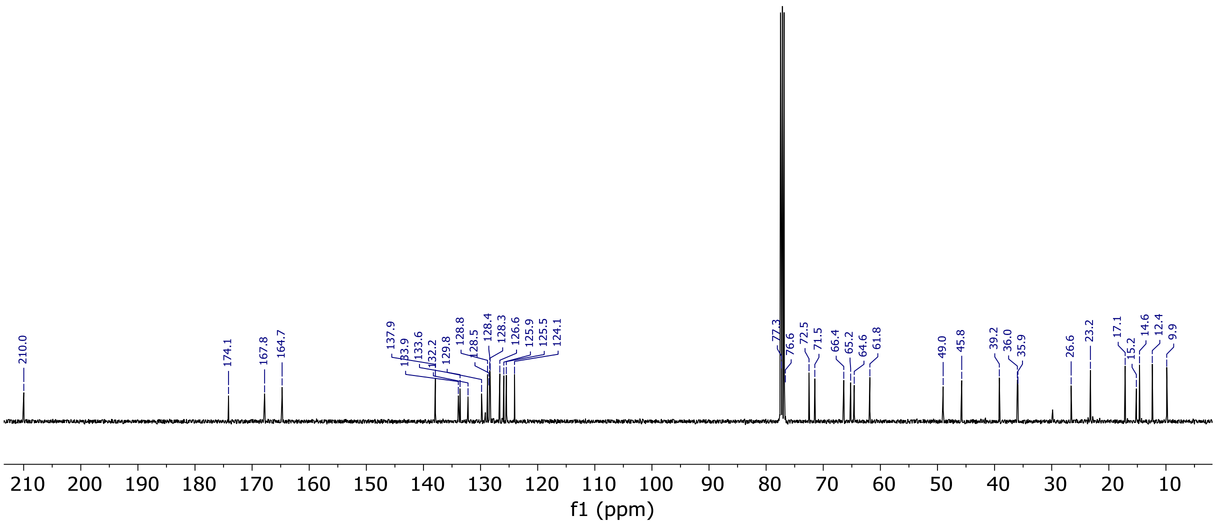

SUW421

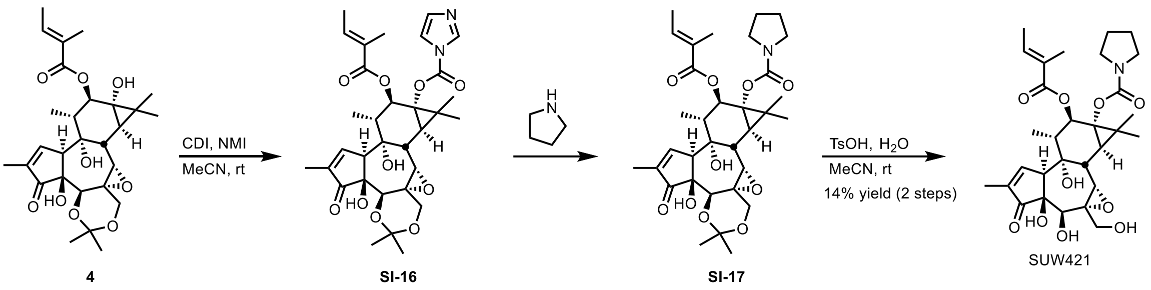

To a flame-dried vial equipped with a stir bar was added **4** (20 mg, 0.039 mmol, 1 equiv) and anhydrous MeCN (2 mL). N-methylimidazole (0.10 mL, 1.25 mmol, 32 equiv) followed by carbonyldiimidazole (21 mg, 0.129 mmol, 3 equiv). The reaction was stirred at rt for 18 hr. TLC analysis indicated complete consumption of **4** and formation of **SI-16**. Pyrrolidine (1.0 mL, dried over sieves) was added directly as a single portion. The reaction was stirred at rt for another 18 hr. TLC analysis indicated complete consumption of **SI-16**. The reaction mixture was diluted with EtOAc (20 mL) and washed with $NaHCO_3$ (20 mL) followed by NH_4Cl (20 mL) and brine (20 mL). The organic layer was dried over Na_2SO_4, filtered, and concentrated. The resulting intermediate **SI-17** was used directly in the next step.

To a vial equipped with a stir bar was added crude **SI-17** followed by MeCN (4 mL). TsOH in water (0.42 M, 4.0 mL) was added (final TsOH concentration of 0.21 M). The reaction mixture was stirred at rt for 15 hr. TLC analysis indicated complete consumption of **SI-17**. The reaction with quenched with sat. $NaHCO_3$ (20 mL) and diluted with brine (20 mL). The aqueous layer was extracted with EtOAc (20 mL). The combined organic layers were dried over Na_2SO_4, filtered, and concentrated. Purification was accomplished by HPLC (method) affording SUW421 (3 mg, 14%) as a white solid. Compound purity was established by TLC (one spot) analysis.

SI-16 TLC R_f = 0.05 (70% Et_2O/Hex, UV active, red spot in *p*-anisaldehyde)
SI-17 TLC R_f = 0.29 (70% Et_2O/Hex, UV active, blue spot in *p*-anisaldehyde)
SUW421 TLC R_f = 0.30 (75% EtOAc/Hex, UV active, blue spot in *p*-anisaldehyde)

^{1}H NMR (600 MHz, $CDCl_3$) δ 7.74 (s, 1H), 6.86-6.78 (m, 1H), 6.82 (br s, 1H) 5.55 (d, *J* = 9.9 Hz, 1H), 4.23 (s, 1H), 4.07 (app p, *J* = 2.9 Hz, 1H), 3.88 (d, *J* = 12.5 Hz, 1H), 3.79 (d, *J* = 12.5 Hz, 1H), 3.49 (dt, *J* = 11.8, 6.2 Hz, 1H), 3.36 (dt, *J* = 11.7, 6.3 Hz, 2H), 3.30 (s, 1H), 3.22 (dt, *J* = 12.6, 6.3 Hz, 1H), 3.16 (d, *J* = 6.7 Hz, 1H), 1.96 (dq, *J* = 10.1, 6.6 Hz, 1H), 1.93-1.84 (m, 4H), 1.83 (s, 3H), 1.78-1.74 (m, 3H), 1.44 (d, *J* = 6.7 Hz, 1H), 1.25 (s, 3H), 1.25 (s, 3H), 0.88 (d, *J* = 6.5 Hz, 3H).

^{13}C NMR (126 MHz, $CDCl_3$, 30 peaks total) δ 210.3, 167.8, 165.4, 155.6, 137.5, 133.4, 128.7, 78.1, 77.2, 72.5, 71.9, 65.6, 65.5, 64.7, 61.6, 49.2, 46.4, 46.2, 45.9, 36.6, 36.3, 26.7, 25.8, 25.0, 24.0, 17.5, 15.4, 14.6, 12.4, 9.9.

HRMS calculated for $C_{30}H_{42}NO_{10}^+$ $[M+H]^+$: 576.2808; found 576.2798.

FTIR (ATR) 3332 (br), 2925, 1707, 1682, 1441, 1410, 1255, 1130, 1076, 1024, 669 cm^{-1}

$[\alpha]^{24}_{D}$ = -21° (*c* = 0.50, CH_2Cl_2)

^{1}H-NMR (600 MHz, $CDCl_3$)

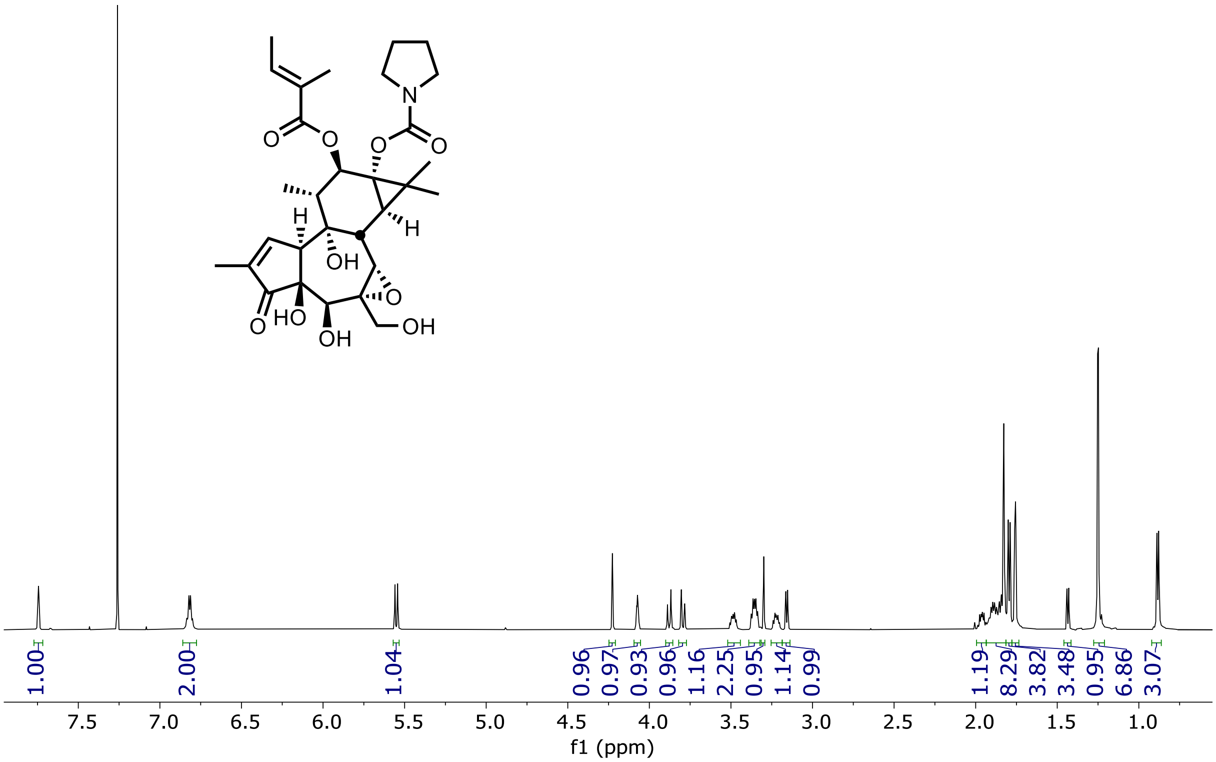

^{13}C-NMR (126 MHz, $CDCl_3$)

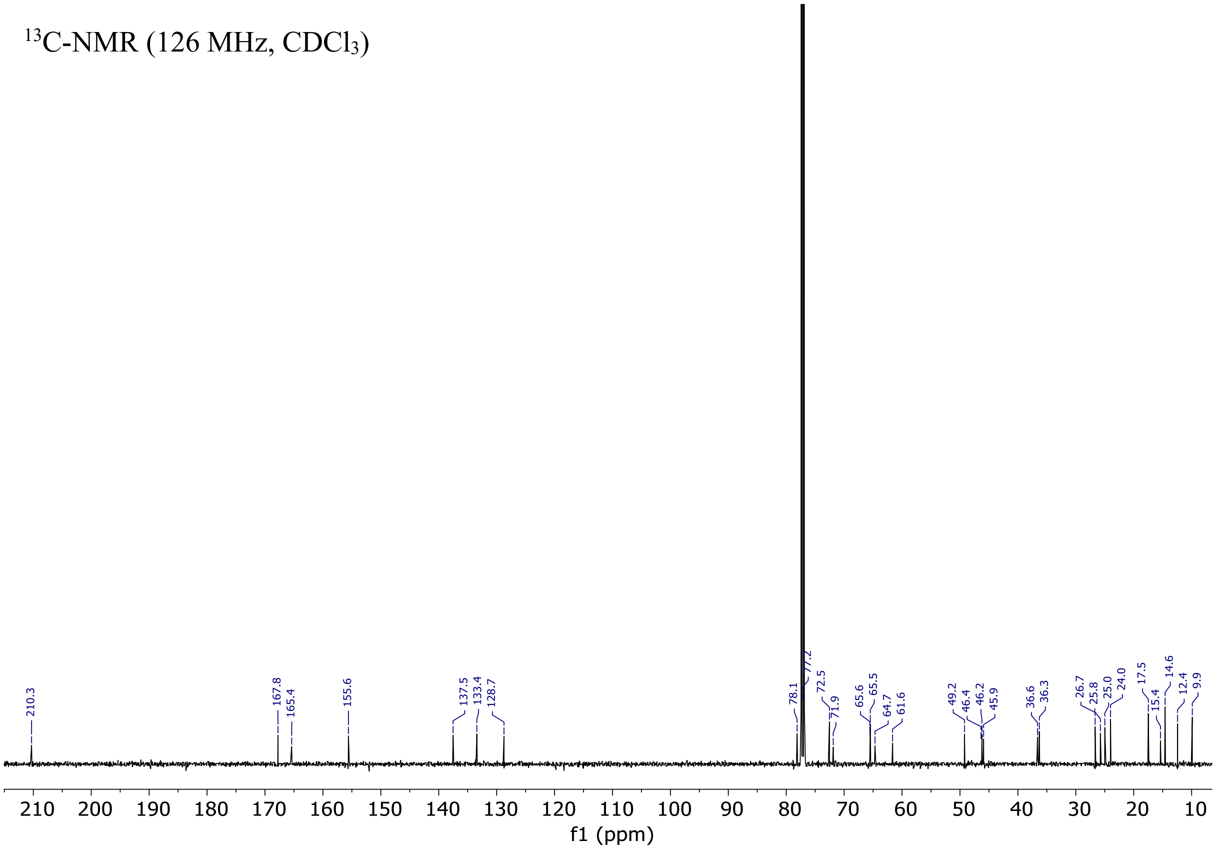

SUW424

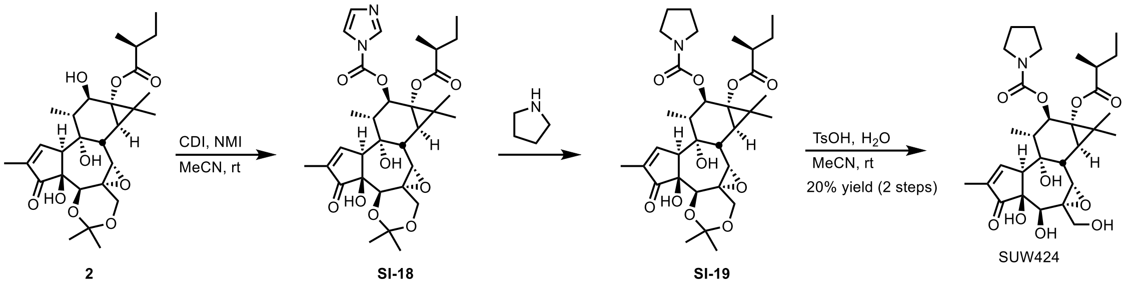

To a flame-dried vial equipped with a stir bar was added **2** (18 mg, 0.035 mmol, 1 equiv) and 1:1 anhydrous MeCN: DCM (1.0 mL). N-methylimidazole (0.10 mL, 1.25 mmol, 36 equiv) followed by carbonyldiimidazole (39 mg, 0.24 mmol, 7 equiv). The reaction was stirred at rt for 18 hr. TLC analysis indicated complete consumption of **2** and formation of **SI-18**. Pyrrolidine (1.0 mL, dried over sieves) was added directly as a single portion. The reaction was stirred at rt for 1 hr. TLC analysis indicated complete consumption of **SI-18**. The reaction mixture was diluted with EtOAc (50 mL) and washed with NH_4Cl (50 mL). The organic layer was dried over Na_2SO_4, filtered, and concentrated. The resulting intermediate **SI-19** was used directly in the next step.

To a vial equipped with a stir bar was added crude **SI-19** followed by MeCN (4 mL). TsOH in water (0.42 M, 4.0 mL) was added (final TsOH concentration of 0.21 M). The reaction mixture was stirred at rt for 15 hr. TLC analysis indicated complete consumption of **SI-19**. The reaction with quenched with sat. $NaHCO_3$ (20 mL) and diluted with brine (20 mL). The aqueous layer was extracted with EtOAc (20 mL). The combined organic layers were dried over Na_2SO_4, filtered, and concentrated. Purification was accomplished by silica gel flash column chromatography (0-30% EtOAc/ether, 1x7 cm) affording SUW424 (4 mg, 20% yield over 2 steps, note 1) as a white foam. Compound purity was established by TLC (one spot) analysis.

Note 1: The poor yield is presumably due to not loading all the compound onto the silica gel column because toluene was used for loading.

SI-18 TLC R_f = 0.05 (70% Et_2O/Hex, UV active, red spot in *p*-anisaldehyde)
SI-19 TLC R_f = 0.31 (70% Et_2O/Hex, UV active, blue spot in *p*-anisaldehyde)
SUW424 R_f = 0.35 (75% EtOAc/Hex, UV active, blue spot in *p*-anisaldehyde)

^{1}H NMR (400 MHz, $CDCl_3$) δ 7.75 (dd, *J* = 2.6, 1.4 Hz, 1H), 5.26 (d, *J* = 9.9 Hz, 1H), 4.23 (s, 1H), 4.06 (app p, *J* = 2.7 Hz, 1H), 3.88 (d, *J* = 12.5 Hz, 1H), 3.80 (d, *J* = 12.5 Hz, 1H), 3.39 (br d, *J* = 6.6 Hz, 2H), 3.30 (br d, *J* = 8.5 Hz, 2H), 3.29 (s, 1H), 3.16 (d, *J* = 6.7 Hz, 1H), 2.41 (app sex, *J* = 7.0 Hz, 1H), 1.96-1.81 (m, 5H), 1.80-1.70 (m, 4H), 1.53-1.41 (m, 1H), 1.26 (d, *J* = 2.5 Hz, 3H), 1.25 (s, 3H), 1.15 (d, *J* = 7.0 Hz, 3H), 0.97-0.93 (m, 3H), 0.92 (d, *J* = 3.3 Hz, 3H).

^{13}C NMR (126 MHz, $CDCl_3$, 30 peaks total) δ 210.2, 179.1, 165.2, 154.9, 133.5, 125.7, 77.6, 72.5, 71.9, 65.7, 65.5, 64.7, 61.8, 49.2, 46.6, 46.4, 46.0, 41.3, 36.2, 36.1, 26.6, 26.3, 25.8, 25.1, 23.9, 17.3, 16.3, 15.3, 11.8, 9.9.

HRMS calculated for $C_{30}H_{44}NO_{10}^+$ $[M+H]^+$: 578.2960; found 578.2956.

FTIR (ATR) 3411 (br), 2959, 2924, 2876, 1703, 1455, 1415, 1261, 1095, 1022, 669 cm^{-1}

$[\alpha]^{23}_{D}$ = -40° (*c* = 0.06, CH_2Cl_2)

^{1}H-NMR (400 MHz, $CDCl_3$)

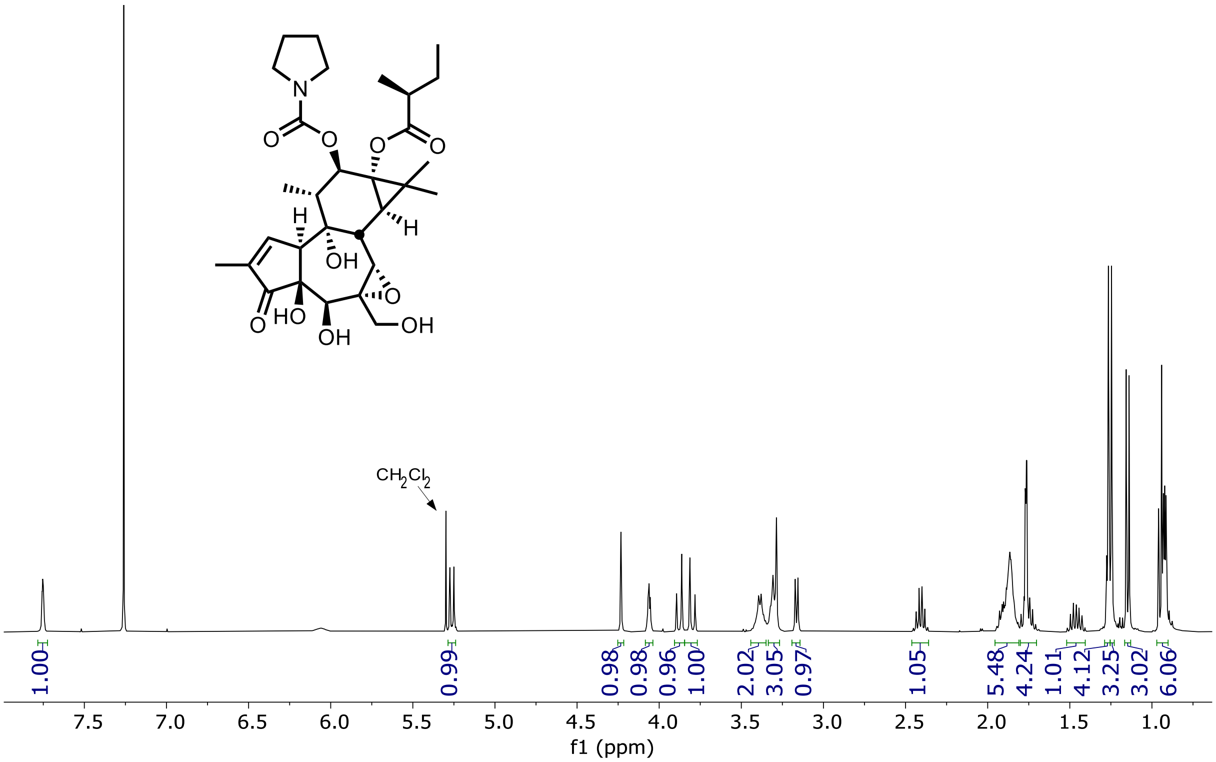

^{13}C-NMR (126 MHz, $CDCl_3$)

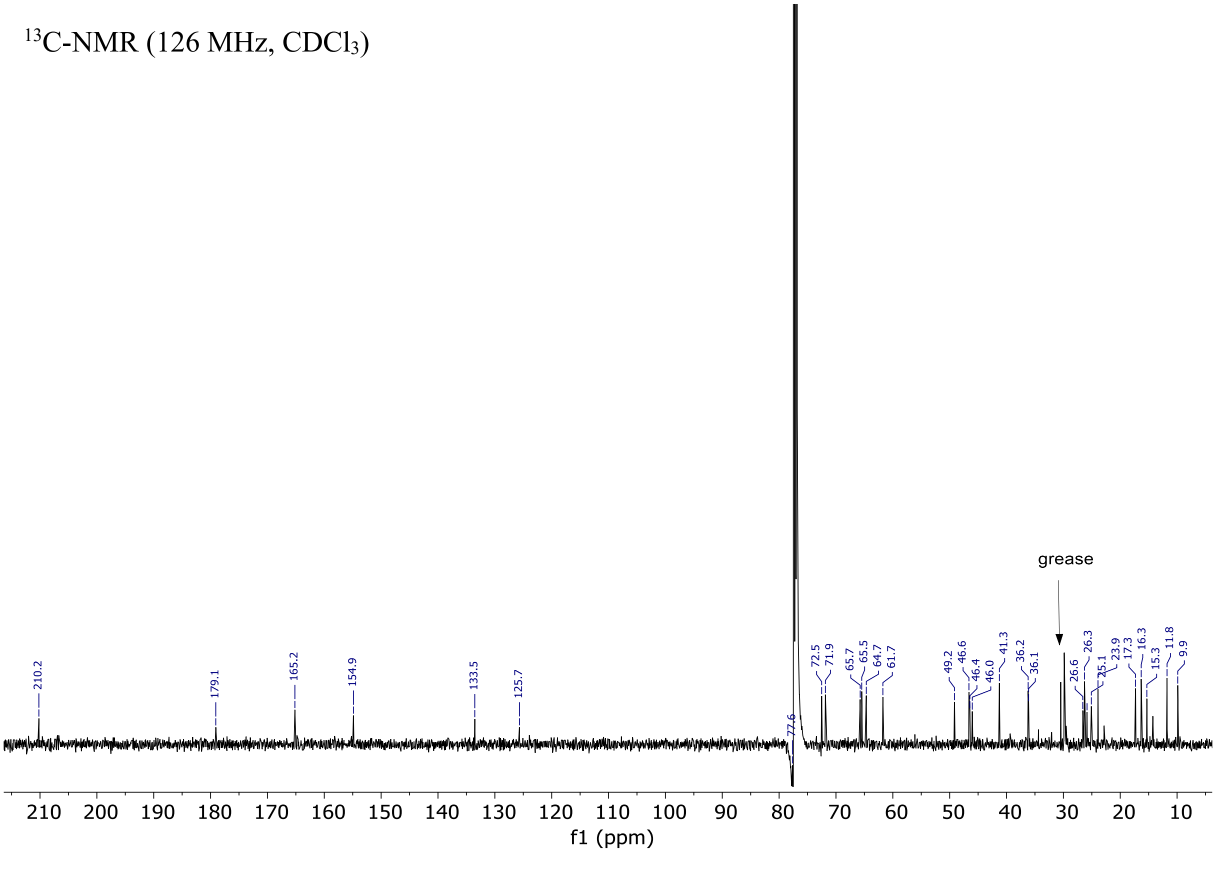

SUW426 preparation

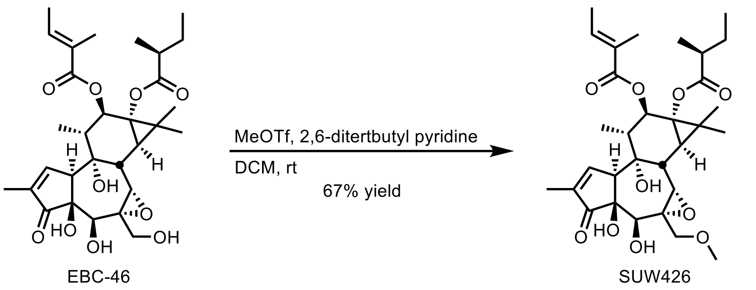

To a flame-dried vial equipped with a stir bar was added EBC-46 (9 mg, 0.016 mmol, 1 equiv) and anhydrous DCM (0.70 mL). 2,6-ditertbutyl pyridine (20 mg, 0.16 mmol, 10 equiv) was added directly as a single portion. The reaction mixture was cooled to 4 °C and methyl triflate (17 µL, 0.096 mmol, 6.0 equiv) was added directly as a single portion. The reaction was allowed to warm to rt and stirred for 18 hr. TLC analysis indicated about 75% consumption of EBC-46 and formation of a new nonpolar product. The reaction mixture was diluted with sat. $NaHCO_3$ (10 mL). The aqueous layer was extracted with EtOAc (3x10 mL). The combined organic layers were dried over Na_2SO_4, filtered, and concentrated. Purification was accomplished by silica gel flash column chromatography (20-50% EtOAc/hexane, 1x7 cm) affording SUW426 (6 mg, 67% yield) as a white solid. Compound purity was established by TLC (one spot) analysis.

SUW426 TLC R_f = 0.54 (70% EtOAc/Hex, UV active, blue spot in *p*-anisaldehyde)

^{1}H NMR (600 MHz, $CDCl_3$) δ 7.70 (app s, 1H), 6.81 (dq, *J* = 7.1, 1.6 Hz, 1H), 5.45 (d, *J* = 9.9 Hz, 1H), 4.27 (d, *J* = 2.8 Hz, 1H), 4.19-4.14 (m, 1H), 4.00 (d, *J* = 11.4 Hz, 1H), 3.76 (br d, *J* = 3.6 Hz, 1H), 3.70 (s, 1H), 3.42 (s, 3H), 3.34 (d, *J* = 11.4 Hz, 1H), 3.17 (d, *J* = 6.5 Hz, 1H), 3.15 (s, 1H), 2.39 (app sex, *J* = 7.0 Hz, 1H), 1.97 (dq, *J* = 9.9, 6.6 Hz, 1H), 1.82 (app s, 3H), 1.81-1.78 (m, 3H), 1.78-1.70 (m, 4H), 1.50-1.42 (m, 1H), 1.25 (s, 3H), 1.24 (s, 3H), 1.14 (d, *J* = 7.0 Hz, 3H), 0.94 (t, *J* = 7.4 Hz, 3H), 0.87 (t, *J* = 6.6 Hz, 3H).

^{13}C NMR (126 MHz, $CDCl_3$, 31 peaks total) δ 209.6, 178.9, 167.6, 164.3, 137.7, 133.5, 128.6, 77.37, 77.36, 74.3, 72.7, 70.0, 65.7, 64.5, 61.9, 60.0, 48.9, 46.0, 41.3, 36.4, 35.9, 26.7, 26.3, 23.9, 17.4, 16.3, 15.2, 14.6, 12.4, 11.8, 9.9.

HRMS calculated for $C_{31}H_{44}O_{10}^+$ $[M+H]^+$: 577.3007; found 577.3003.

FTIR (ATR) 3398 (br), 2963, 2922, 2854, 1711, 1458, 1377, 1257, 1072, 1018, 800, 669 cm^{-1}

$[\alpha]^{23}_D$ = -50° (*c* = 0.05, CH_2Cl_2)

^{1}H-NMR (400 MHz, CDCl$_3$)

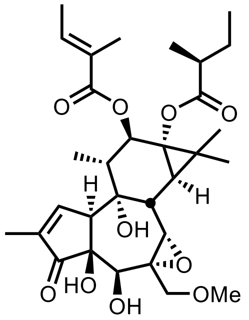

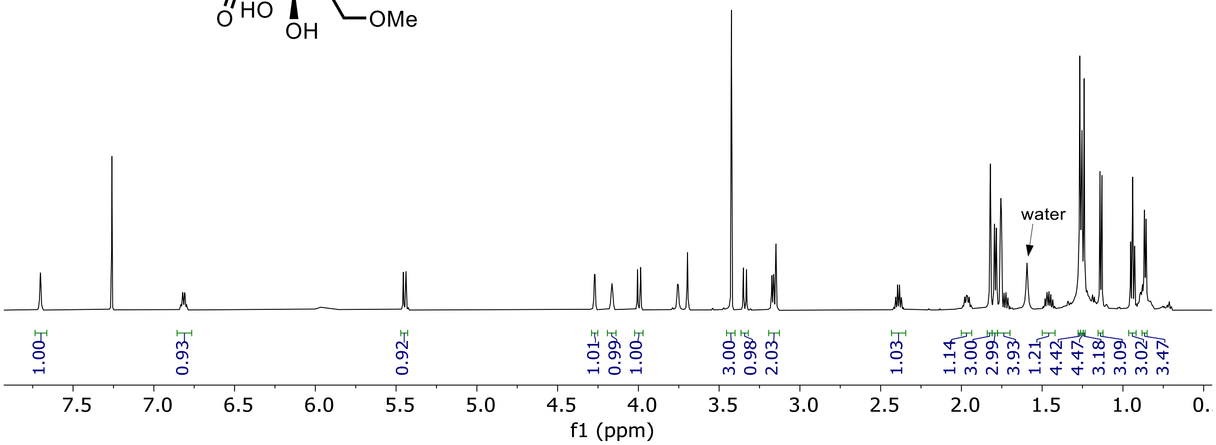

^{13}C-NMR (126 MHz, CDCl$_3$)

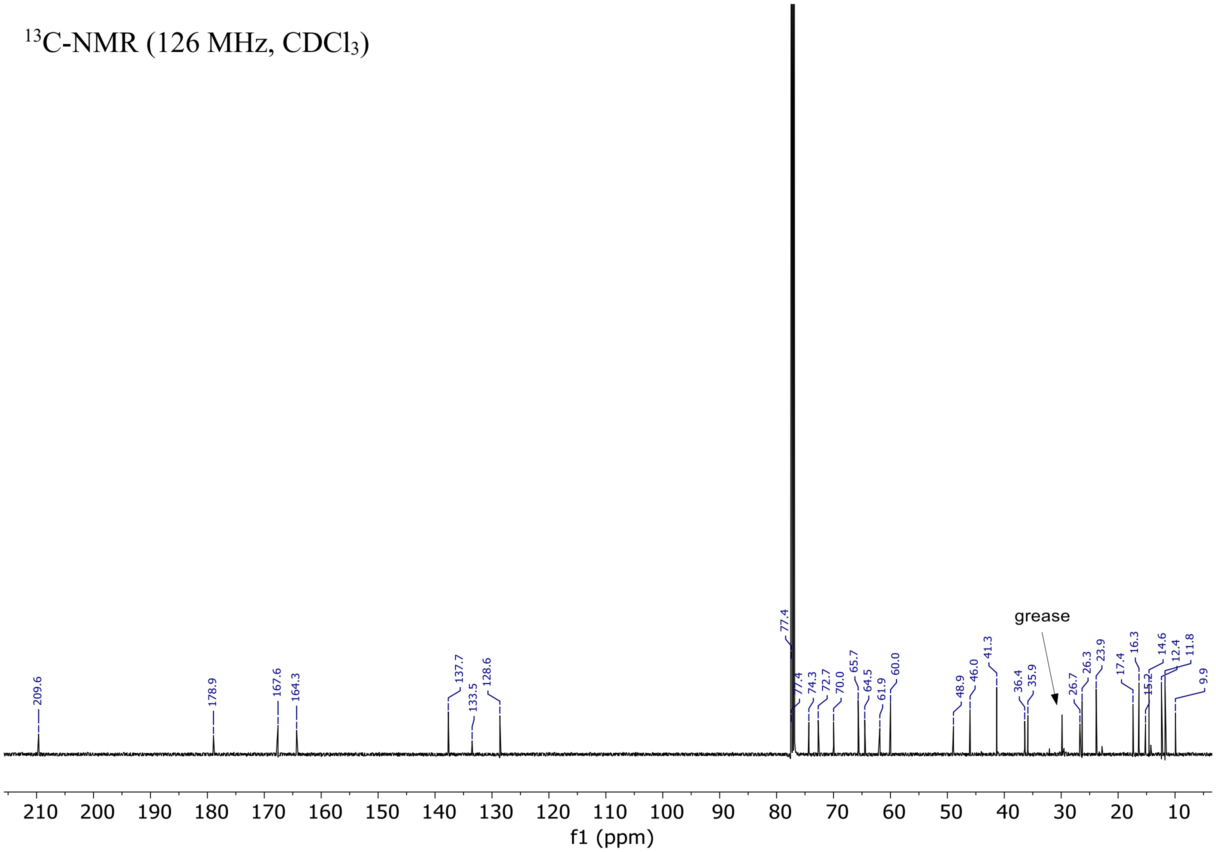

Improved EBC-46 synthesis step (C12, C13 acetate protection)

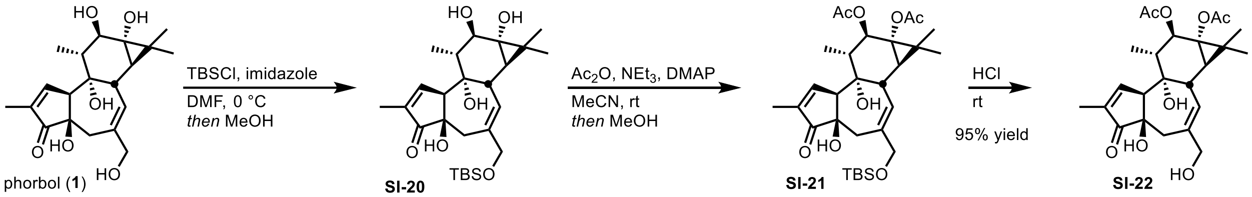

To a one-neck 1L round-bottom flask equipped with a stir bar was added **1** (10.0 g, 27.4 mmol, 1 equiv) and DMF (196 mL). The reaction mixture was sonicated for 10 minutes until phorbol dissolved. Imidazole (7.5 g, 109.6 mmol, 4.0 equiv) was added directly as a single portion. The reaction mixture was cooled to 0 °C and TBSCl (12.4 g, 82.2 mmol, 3 equiv) was added directly as a solid as a single portion. TLC analysis indicated complete consumption of **1**. The excess TBSCl was quenched by addition of MeOH (3.9 mL, 96 mmol, 3.5 equiv) in one portion on ice. NEt_3 (45.6 mL, 329 mmol, 12 equiv), Ac_2O (31.2 mL, 329 mmol, 12 equiv), and DMAP (3.4 g, 27.4 mmol, 1 equiv) were added and the walls of the flask were rinsed with MeCN (196 mL). The cloudy reaction mixture was allowed to warm to room temperature. After stirring for 15 minutes, TLC analysis indicated complete consumption of **SI-20** to form intermediate **SI-21**. MeOH (14.4 mL) was added dropwise at room temperature followed by 1 M HCl (422 mL, 15.4 equiv). The reaction mixture became colorless, was sparged with nitrogen, sealed, and stirred at rt for 17 hr.

TLC analysis indicated complete consumption of **SI-21** and formation of **SI-22**. The reaction mixture was diluted with ethyl acetate (500 mL) and brine (1.0 L). The aqueous layer was saturated with excess NaCl and extracted with ethyl acetate (2 x 500 mL). The combined organic layers were washed with sat. $NaHCO_3$ (500 mL) and brine (500 mL). The secondary aqueous washes were combined and back extracted with ethyl acetate (500 mL). The combined organic layers were dried over Na_2SO_4, filtered, and concentrated. Purification was accomplished by silica gel vacuum column chromatography (10-55% EtOAc/toluene, 9x15 cm) affording phorbol diacetate **SI-22** (16.6g, 95% yield – determined by qNMR) as a white foam. Compound purity was established by TLC (one spot) analysis.

All characterization data of phorbol diacetate **SI-22** prepared with this reaction was identical to that which was previously reported (22).

Improved EBC-46 synthesis step (installation of C6,C7 α-epoxide)

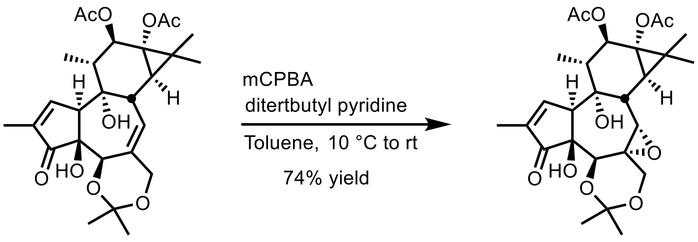

SI-23 **3**

To a one-neck 100 mL round-bottom flask equipped with a stir bar was added **SI-23** (1.526 g, 3.024 mmol, 1 equiv) and toluene (51.0 mL). 2,6-ditertbutyl pyridine (4.6 mL, 19.96 mmol, 6.6 equiv) was added directly as a single portion while stirring. The reaction mixture was cooled to 10 °C and m-chloroperbenzoic acid (77%, 3.44 g, 15.4 mmol, 5.1 equiv) was added directly as a solid as a single portion while stirring. The reaction was sealed, allowed to warm to rt, and stirred for 48 hr. TLC analysis indicated about complete consumption of **SI-23**. The reaction was quenched with 2-methyl-2-butene (1.6 mL) and diluted with ethyl acetate (2 mL). The reaction mixture was stirred at rt for 1 hr to quench remaining mCPBA.

The reaction mixture was diluted with ethyl acetate (300 mL) and washed with 400 mL of a 1:1 v/v mixture of sat. $NaHCO_3$ and sat. $Na_2S_2O_3$ and then sat. $NaHCO_3$ (5 x 100 mL) until the aqueous washes were no longer pink. TLC analysis indicated all product **3** was present in the organic layer. The combined organic layers were dried over Na_2SO_4, filtered, and concentrated. Purification was accomplished by silica gel flash column chromatography (10-36% EtOAc/hexane, 5.5x7 cm) affording **3** (1.27g, 74% yield) as a white foam. Compound purity was established by TLC (one spot) analysis.

All characterization data of epoxide **3** prepared with this reaction was identical to that which was previously reported (22).

Cell-free PKC Binding Assay

The protein kinase C (PKC) affinity of EBC-46 and EBC-46 analogs was determined via competition with 3H-phorbol-12,13-dibutyrate (3H-PDBu) as described below. This procedure entails a glass-fiber filtration method to determine bound radioligand. The method of determining the radioligand Kd is also described.

Chemicals/Materials:

- Water (MilliQ)
- Tris HCl (Fisher, ≥ 99%, stored at room temperature)
- Phosphatidylserine (Avanti Polar Lipids, porcine, 25 mg/mL CHCl3 solution, stored at -20 °C)
- Bovine serum albumin (Sigma-Aldrich, ≥ 98%, globulin free, protease free, stored at 4 °C)
- 3H-PDBu (American Radiolabeled Chemicals, Inc.; 1 mCi/mL acetone solution)
- Human PKC-β-I-GST fusion protein (Invitrogen, aliquoted and stored at -78 °C)
- Human PKC-δ-GST fusion protein (Invitrogen, aliquoted and stored at -78 °C)
- Glass-fiber filters (Whatman GF/B)

Preparation of 3H-PDBu solution:
3H-PDBu (1 mCi/mL acetone solution; specific activity: 20 Ci/mmol) was diluted with DMSO to a final concentration of 500 nM.

Preparation of phosphatidylserine (PS) vesicles:
For every 96 samples, 3.5 mg phosphatidylserine (140 µL) (25 mg/mL CHCl3 solution) was concentrated by removing chloroform under reduced pressure. The phosphatidylserine residue was resuspended in freshly prepared PKC binding assay buffer (3.5 mL), capped, and then thoroughly mixed by alternating between sonicating and vortexing, in 10 second intervals for 1 minute or until a cloudy white mixture (Branson Sonifier 250, power = 2, 50% duty cycle). The resulting cloudy white mixture (1 mg/mL) was stored on ice until use.

Preparation of the "master mix" Solution:
Constituent Stock concentration (final concentration/amount in “master mix”):

- pH 7.4 Tris-HCl 1.0 M (50 mM)
- KCl – 1.0 M (100 mM)
- $CaCl_2$ – 0.10 M (0.12 mM)
- BSA – Solid (2 mg/mL)
- PKC-GST (37.3 ng/sample)
- PS vesicles (0.103 mg/mL)
- Deionized H_2O - Final vol of 30 mL

Preparation of PKC binding assay buffer:
For every 96 samples, to a 50 mL polypropylene tube was added Tris-HCl (pH 7.4, 1 M, 1.5 mL), KCl (1 M, 3 mL), $CaCl_2$ (0.1 M, 35 µL – conventional isoforms only), and bovine serum albumin (60 mg). This mixture was diluted to 30 mL with deionized H_2O and mixed gently. The buffer was stored on ice until use.

Preparation of PKC isoform solution:
For every 96 samples, PKC isoform solution was prepared by dissolving a 4 μg aliquot of the indicated recombinant human PKC isoform into 26.5 mL of PKC binding assay buffer. The diluted PKC was stored on ice for immediate use.

Preparation of PKC "master mix" solution:
To the PKC-isoform solution was added 1 mg/mL PS vesicles solution (3.30 mL), and 500 nM 3H-PDBu solution (0.150 mL). The resulting "master mix" solution was vortexed and stored on ice.

Preparation of analog compound dilutions:
Compound dilutions were prepared with filtered DMSO by serially diluting from a chosen "high" concentration. For the saturation binding experiment (radioligand Kd determination), the maximum radioligand concentration used was 4.0 nM with 2-fold dilutions between data points (0.0625 nM, 0.125 nM, 0.25 nM, 0.50 nM, 1.0 nM, 2.0 nM, and 4.0 nM) (Note 1).

Note 1: The concentrations chosen for the saturation binding assay did not exceed 4.0 nM because the standard error in non-specific binding at high concentrations resulted in highly inaccurate and inconsistent Kd values. Literature values for the Kd of 3H-PDBu for conventional and novel PKC isoforms ranges from 0.3 – 0.8 nM. This concentration window also places [3H-PDBu] = Kd in the middle of the binding curve.

For the competition binding experiment (analog Ki determination), 7 or 14 concentrations were used to define the inhibition curve, and compounds dilutions changed by factors of 2 or √2 between data points. The concentrations were chosen such that the in-assay concentration for the data points in the middle of the binding curve were approximately equal to the IC50 of the compound (if the IC50 is unknown, an exploratory binding assay is recommended prior to Ki determination). For assays with one compound per sheet, the √2-fold dilutions were prepared for the concentrations in the middle of the curve (e.g., for SUW400, the analog dilutions used were 15000 nM, 7500 nM, 3750 nM, 1875 nM, 938 nM, 663 nM, 469 nM, 332 nM, 235 nM, 117 nM, 59 nM, 29 nM, 15 nM, 7 nM).

PKC binding assay protocol (Saturation-binding experiment and Competition-binding experiment)
1) Glass-fiber filters were prepared by soaking them in a solution of aqueous polyethyleneimine (10% by vol, 18 mL) in deionized water (600 mL) for ≥1h prior to use. 1 M Tris HCl buffer was prepared with Tris HCl and acidified to pH 7.4 with concentrated aqueous HCl (stored at 4 °C for one month). 3000 mL of 20 mM Tris buffer (pH 7.4) was prepared from the 1 M stock solution and cooled to 4 °C for ≥ 1 hour prior to use (prepared fresh). 1.0 M KCl solution and 0.10 M $CaCl_2$ solution were stored at room temperature. The incubator was preheated to 37 °C.

2) Samples were obtained in triplicate for each data point. For the saturation binding assay (Kd determination), each sample was prepared with 260 μL of "Master Mix" solution and 40 μL of each radioligand dilution. For the competition binding assay (Ki determination), each sample was prepared with 280 μL of "Master Mix" solution and 20 μL of each compound dilution. For the saturation binding experiment, 3H-PDBu binding was assessed at seven concentrations (0.0625 nM to 4.0 nM) without PKC (non-specific binding) and with PKC (specific + non-specific binding)

and a DMSO negative control (48 samples total). For the competition binding assay, compounds were tested at either 7 or 14 different concentrations (two compounds per sheet or one compound per sheet), and the positive control was 5 μM PDBu (in assay concentration).

3) Samples were prepared by first adding "Master Mix" to glass test tubes and leaving them in an ice-water-cooled test-tube rack (Note 2). Compound dilutions were added to the "Master Mix"-filled tubes in triplicate at 4 °C (Note 3). The tubes were vortexed and returned to the 4 °C ice water-cooled test-tube rack until all samples for one sheet were ready for incubation. Using a partially water-filled thermal block incubator (Note 4), the assay samples ("Master Mix" + compound dilution) were placed directly in the incubator at 37 °C for 5 minutes. After incubation, the samples were returned to the ice-water cooled test-tube rack for at least 10 minutes prior to filtration on the harvester.

Note 2: "Master Mix" was carefully dispensed in the bottom half of the tubes without touching the walls of the test tube with the outside of the pipet tip. The outside of the pipet tip was wiped off with a chemwipe between tubes. This was done to avoid residual droplets on the interior of the tube walls.
Note 3: If using frozen DMSO dilutions, all samples were vortexed after thawing to ensure homogeneity.
Note 4: Adding water to the wells of the heating blocks ensured that the samples had uniform heating contact.

4) Using a Brandel Harvester, the sample contents of each polypropylene tube were vacuum-filtered through polyethylenimine-soaked filters, washed with rinsing buffer (3x) and dried under vacuum for 5 minutes. Following harvesting, the filter sheets were allowed to air dry at room temperature until all sheets for the assay had been harvested. The resulting filters had circular perforations for each data point, which were removed with forceps and placed into scintillation vials. The scintillation vials were filled with Bio-Safe II scintillation fluid (5 mL) and measured for radioactivity using a Beckman LS 6000SC scintillation counter.

5) Counts per minute (cpm) were averaged for each triplicate dilution. The data were plotted – cpm vs. log(concentration) – using PrismR by GraphPad Software and an IC50 was determined using the same program's built-in one-site competition least squares regression function. Ki values were calculated using the equation: Ki = IC50 / (1 + ([3H-PDBu] / Kd)). The Kd of 3H-PDBu for PKC isoforms was measured separately via saturation binding experiments. The Kd values were determined each time a new batch of protein was used and were typically between 0.3 nM and 0.8 nM (i.e. 0.37 nM for PKC-β and 0.62 nM for PKC-δ – consistent with literature reports (63). The legitimacy of the Kd determination assays was confirmed by Scatchard analysis of the saturation binding curve and visual inspection of the Scatchard plot.

Cell maintenance

Human A549-Dual™ Cells supplied by InvivoGen (Cat. code a549d-nfis) and CHO-K1 cells were passaged when about 90% confluency was reached (approximately every three days). With each passage, media was aspirated from the cell layer, which was then washed with PBS. Following aspiration of the PBS, cells were detached from the culture flask with 0.25% Gibco™ Trypsin-EDTA and transferred to a 50 mL conical, which was volumed up with complete pre-warmed DMEM (4.5 g/L glucose, 10% FBS, and 1% Pen-Strep-Glutamine (100 X, 50 mg/mL)) or Ham's F-12K (Kaighn's) Medium supplemented with 10% FBS and 1% Pen-Strep (100 X, 50 mg/mL). This conical was spun down at 1800 rpm for 3 minutes, the supernatant was removed, and cells were resuspended in their respective fresh, pre-warmed media. To count cells, 10 µL of resuspended cells were added to a sterile microcentrifuge tube and mixed with 10 µL of trypan blue after which 10 µL of the mixture was pipetted into a Countess chamber slide. A Countess™ 3 Automated Cell Counter was subsequently utilized to determine cell viability and concentration. 1×10^6 cells were seeded with each passage into a T75 culture flask, and cells were incubated at 37 °C in 5% CO_2.

NF-κB assay

Human A549 lung carcinoma cells (InvivoGen Cat. code a549d-nfis), stably integrated with a secreted embryonic alkaline phosphatase (SEAP) reporter, allow for the activity of the NF-κB pathway to be monitored. The SEAP reporter is under control of the interferon β (IFN-β) minimal promoter, which is fused to five NF-κB binding sites.

5×10^4 A549 cells were plated on Corning® 96 Well TC-Treated Microplates (CLS3595) in 180 µl of pre-warmed DMEM (4.5 g/L glucose, 10% heat-inactivated FBS (30 min at 56 °C), and 1% Pen-Strep-Glutamine (100 X, 50 mg/mL)). Note that if cells were grown in media containing FBS that is not heat-inactivated, media will contain alkaline phosphatase. In this case, all supernatant must thoroughly be aspirated from the cell pellet obtained after centrifugation before resuspending cells in media containing heat inactivated FBS for plating. Following incubation at 37 °C in 5% CO_2 for approximately 24 hours to allow for cell adherence onto the 96 well plate, 1.8 µL of each compound dissolved in DMSO was added in quadruplicate to their respective wells to achieve a final compound concentration of 1500 nM, 500 nM, or 50 nM. 1.8 µL of DMSO was added to wells as negative controls while wells treated with EBC-46 served as positive controls. 24 hours following compound addition, 180 µL of QUANTI-Blue™ Solution (InvivoGen Cat. code rep-qbs)- medium containing QB buffer and QB Reagent for detection and quantification of alkaline phosphatase- were added into wells on a new clear flat-bottom 96-well plate. 20 µL of supernatant of either SEAP-expressing cells incubated with compound or SEAP-expressing cells incubated with DMSO only were added to the wells in the new 96-well plate containing QUANTI-Blue™ Solution. Supernatant and QUANTI-Blue™ Solution were incubated at 37 °C in 5% CO_2 for approximately an hour and a half. Following incubation, optical density (OD) at 630 nm was measured for each well using a BioTek Synergy H1 microplate reader under normal read speed and with a delay of 100 msec.

Bacterial culture

Agar stabs of DH5-alpha (high copy) bacterial cells, containing plasmids for expression of mammalian PKC-δ (Plasmid #: 178856) and PKC-β (Plasmid #: #112265) fused to EGFP under constitutive mammalian promoters CAG and CMV, respectively, were purchased from Addgene. Upon receipt, these stabs were incubated at 37 °C in 5% CO_2 for 24 hours to allow sufficient bacterial growth. Following incubation, a sterile pipette tip was utilized to streak bacteria onto LB agar plates that are positive for the respective antibiotic that selects for each plasmid. For bacteria containing Plasmid #: 178856, bacteria were streaked onto CellPro™ LB (Lennox) + Ampicillin 100 μg/ml while bacteria containing Plasmid #: 112265 was streaked onto CellPro™ LB (Miller) + Kanamycin 50 μg/ml and incubated for 24 hours at 37 °C in 5% CO_2 in order to isolate single colonies of bacteria. Single, isolated bacterial colonies were selected utilizing a sterile pipette tip and placed in polypropylene culture tubes with vented stoppers containing 2mL of LB Broth Miller (Catalog No. BP1426-500) and the respective antibiotic for each plasmid at the recommended concentration of 100 μg/ml for ampicillin and 50 μg/ml for kanamycin. Following inoculation, these liquid cultures were placed on a shaker at 37 °C at 225rpm for 24 hours. The next day, the plasmids were sequestered and purified from the bacteria utilizing the Thermo Scientific™ GeneJET Plasmid Miniprep Kit (Catalog No. K0502).

PKC-β/PKC-δ plasmid purification

To sequester and purify plasmid from each culture tube, bacteria was resuspended in culture and transferred to sterile 1.7mL microcentrifuge tubes with 1 mL of bacteria placed into each tube. To harvest the bacteria, microcentrifuge tubes were placed in a microcentrifuge and spun at 8000 rpm for 2 minutes at room temperature. Note that all subsequent centrifugations were performed in a tabletop microcentrifuge at room temperature. Following centrifugation, the supernatant was decanted and 250 μL of Resuspension solution was added to the pelleted cells and vortexed. Afterwards, 250 μL of Lysis Solution was added to the resuspended bacterial cells and tubes were inverted 4-6 times and allowed to incubate for no more than 5 minutes to avoid denaturation of the plasmid DNA. After lysing the bacterial cells, 350 μL of Neutralization Solution was immediately added and tubes were inverted 4-6 times. Tubes were then centrifugated for 5 minutes at 14,000 rpm to pellet the cell debris and chromosomal DNA. The supernatant was then carefully aspirated using a pipette, taking care not to distribute the white precipitate of cell and DNA debris and transferred to the GeneJET spin columns, provided in the GeneJET Miniprep Kit. The spin columns were subsequently centrifugated for 1 minute, the flow-through was discarded, and the column was placed back into the same collection tube. 500 μL of Wash Solution, diluted with 96% ethanol, was added to the column, and the column was centrifugated for 1 minute at 14,000 rpm. The flow-through was discarded and the column was placed back into the same collection tube. This wash process was repeated a second time, after which the spin columns were centrifugated for an additional 1 minute to ensure all residual Wash solution was removed from the plasmids. The GeneJET spin columns were then transferred to new 1.7mL microcentrifuge tubes, and 50 μL of Elution Buffer was added to the center of the spin column to allow optimal elution of the plasmid DNA. Before centrifugating for 2 minutes, the columns were allowed to incubate for 2 minutes at room temperature. Following centrifugation, the spin columns were discarded, and the purified plasmid was stored at -20 °C. A Thermo Scientific NanoDrop One UV-Vis Spectrophotometer was used to quantify the concentration of plasmid sequestered in each microcentrifuge and verify its purity, using the ratio of absorbance at 260 and 280 nm. A ratio of ~1.8 is accepted as pure. Samples of plasmid were subsequently sent out for sequencing verification.

PKC-β/PKC-δ plasmid transfection of CHO-K1 cells

3 x 10^5 CHO-K1 cells were seeded to 70-90% confluency in 6-well Clear Flat Bottom TC-treated Multiwell Cell Culture Plates in Ham's F-12K (Kaighn's) Medium supplemented with 10% FBS and 1% Pen-Strep (100 X, 50 mg/mL)). A master mix of dilute Lipofectamine 3000 Reagent in Opti-MEM™ Medium was prepared such that each well would receive 3.75 μL of Lipofectamine™ 3000 Reagent diluted in 125 μL of Opti-MEM™ Medium. This master mix was vortexed for 2-3 seconds. A second master mix containing either PKC-β or PKC-δ plasmid DNA, Opti-MEM™ Medium, and P3000™ Reagent was prepared such that each well would receive 2500ng of plasmid DNA and 5 μL of P3000™ Reagent suspended in 125 μL of Opti-MEM™ Medium. Plasmid DNA was first diluted in Opti-MEM™ Medium and then P3000™ Reagent was added to the master mix, which was subsequently mixed by gently pipetting the mixture up and down. The master mix containing plasmid DNA, Opti-MEM™ Medium, and P3000™ Reagent was then added to the master mix of dilute Lipofectamine 3000 Reagent in Opti-MEM™ Medium in a drop-wise fashion (1:1 ratio) and allowed to incubate at room temperature for 10-15 minutes. Following incubation, 250 μL of DNA-lipid complex was added to each respective well of CHO-K1 cells in Ham's F-12K (Kaighn's) Medium supplemented with 10% FBS and 1% Pen-Strep (100 X, 50 mg/mL)). After incubating cells for approximately 16 hours at 37 °C in 5% CO_2, cell culture media was aspirated, cells were washed with PBS, and 1mL of 0.25% Gibco™ Trypsin-EDTA was added to each well. After cell detachment occurred, cells were transferred to a conical, which was volumed up with pre-warmed Ham's F-12K (Kaighn's) Medium supplemented with 10% FBS and 1% Pen-Strep (100 X, 50 mg/mL). This conical was spun down at 1800 rpm for 3 minutes, the supernatant was removed, and cells were resuspended in fresh, pre-warmed Ham's F-12K (Kaighn's) media. Approximately 6 x 10^5 CHO-K1 cells, suspended in 400 μL of media, were added to the 20 mm micro-wells of 35 mm glass bottom dishes from Cellvis (#1.5 cover glass) (Catalog # D35-20-1.5-N). After cell adherence occurred within the 20 mm micro-wells (approximately 8 hours after seeding), additional media was added to the glass bottom dishes, and cells were allowed to incubate at 37 °C in 5% CO_2 for approximately 24 hours before imaging.

PKC-β/PKC-δ translocation

Prior to cell imaging at 37 °C, culture media was aspirated and replaced with 200 μL of Dulbecco's Modified Eagle Medium containing no phenol-red (4.5 g/L D-glucose, L-Glutamine, 25 mM HEPES). Cells expressing fluorescent PKC-β or PKC-δ were located under a Nikon Ti2 Crest Spinning Disk Confocal Microscope, using the 488-nm argon excitation laser and 60x oil lens. 200 μl of compound was added to the respective dish at a 2 times higher concentration to achieve the desired final concentration for translocation observation. The cells were scanned for approximately 30 minutes and images were acquired periodically.

HIV latency reversal *in-vitro* assay

Human T Lymphocyte J-Lat Full Length Cell Line 10.6 (cat #9849) was obtained through the NIH HIV Reagent Program, Division of AIDS, NIAID, NIH and was contributed to the NIH HIV Reagent Program by Dr. Eric Verdin (70). J-Lat 10.6 cells were plated at 5×10^5 cells in 180 µL of RF10 media consisting of RPMI media supplemented with 1% Penicillin/Streptomycin and 10% Fetal Bovine Serum (FBS) in 96-well U-bottom plates. J-Lat 10.6 cells were then stimulated for 48 hr by adding 20 µL of newly designed and synthesized tigilanol tiglate analogs, PKC modulators (bryostatin 1) as our positive control or media only (mock) as our negative control. Our negative control was RF10 media as the tigilanol tiglate analogs were freshly diluted with RF10 prior to plating. The final volume was 200 µL per well. Experiments were performed in three independent biological replicates, each completed in technical duplicates (n = 6). After 48 hr stimulation, 96-well U-bottom plates were centrifuged at 296 RCF for 7 minutes to pellet cells, which were resuspended in 200 µL of freshly prepared 2% paraformaldehyde (PFA) in PBS. GFP expression was measured 24-hours post-fixation using the FITC channel on a LSR Fortessa (BD Biosciences) flow cytometer. Data analysis was conducted with FlowJo software version 10.8.1.

Enzyme Linked Immunosorbent Assay (ELISA) for SUW402 and SUW431 mediated IL-8 and TNF-α expression

In efforts to replicate the conditions of the in vitro NF-κB assay, 5 x 10^4 A549 cells were plated per well on Corning® 96-well Clear Round Bottom TC-treated Microplates in 180 µl of pre-warmed DMEM (4.5 g/L glucose, 10% FBS, and 1% Pen-Strep-Glutamine (100 X, 50 mg/mL)). Following incubation at 37 °C in 5% CO2 for approximately 24 hours to allow for cell adherence, 1.8 µl of either SUW402 or SUW431 were added in triplicate to their respective wells to achieve a final compound concentration of either 50 nM or 500 nM, the two concentrations tested in the NF-κB assay. 24 hours after compound addition, the microplate containing cells treated with compound was spun at 1800 rpm for 3 minutes to allow for pelleting of cells and other debris before 150 µL of cellular supernatant was aspirated and transferred to a new Corning® 96-well Clear Round Bottom TC-treated Microplate. Note that if the supernatant is not meant to be immediately analyzed, the microplate containing cellular supernatant should be parafilm, wrapped, and stored at -80 °C. Careful attention should be paid to avoiding freeze-thaw cycles. To quantify cytokine secretion within samples, dilutions were performed using Assay Buffer A included in the LEGEND MAX™ Human IL-8 ELISA Kit (Cat # 431507) and LEGEND MAX™ Human TNF-α ELISA Kit (Cat # 430207), respectively.

Immediately before running the assay, reagents were allowed to come to room temperature and diluted. Note that if the supernatant is frozen, it should be removed at this time and allowed to thaw. The 1X Wash Buffer was prepared by diluting the 20X Wash Buffer with Invitrogen™ UltraPure™ DNase/RNase-Free Distilled Water. The lyophilized human IL-8 and TNF-α standards were reconstituted by adding the appropriate volume of Assay Buffer A to prepare the 20 ng/mL standard stock solutions. The reconstituted standards were allowed to sit at room temperature for 20 minutes before being gently vortexed to ensure complete mixing. To generate a standard curve for each cytokine, 500 µL of a 1,000 pg/mL top standard was prepared after which six two-fold serial dilutions of the top standard were performed in 1.7 mL microcentrifuge tubes using Assay Buffer A as the diluent.

The Anti-Human IL-8 pre-coated 96-well Strip Microplate and Anti-Human TNF-α pre-coated 96-well Strip Microplate were each washed 4 times by hand. With each wash, at least 300 µL of 1X Wash Buffer was added per well and aspirated, and any residual buffer was removed by blotting the plate on an absorbent paper towel. Following this wash cycle, 50 µL of Assay Buffer A was added to each well that would ultimately contain sample or standard. To rigorously ensure no cellular debris was left in the analyte, an additional centrifugation step was performed (1800 rpm for 3 minutes) before transferring 50 µL of sample or standard dilutions to the appropriate wells. Adhesive plate sealers were utilized to cover the plates, prevent contamination, and avoid sample evaporation after which each plate was placed on a cell shaker and allowed to shake at room temperature at 200 rpm for 2 hours. Following incubation, the contents of each plate were discarded and subsequently washed by hand 4 times with 1X Wash Buffer, as previously reported. 100 µL of human IL-8 or human TNF-α detection antibody was added to each well on their respective plates, and the plates were sealed with a plate sealer and allowed to shake for 1 hour at 200 rpm. Following incubation, the contents of each plate were discarded and subsequently washed by hand 4 times with 1X Wash Buffer. 100 µL of Avidin-HRP A or Avidin-HRP B were added to the IL-8 and TNF-α plates, respectively. The plates were then sealed with a plate sealer and allowed to incubate at room temperature while shaking for 30 minutes at 200 rpm. The contents of each

plate were discarded and subsequently washed by hand 5 times with 1X Wash Buffer and, between each wash, blotted on a paper towel to remove residual buffer. Unlike previous wash cycles, however, each plate was allowed to incubate for 1 minute between each wash to ensure that background was minimized. Following this final wash step, 100 μL of Substrate Solution F or Substrate Solution D were added to the IL-8 and TNF-α plates, respectively. Each plate was allowed to incubate for 15 minutes in the dark. Wells with detectable cytokine turned blue with an intensity that was directly proportional to cytokine concentration. 100 μL of stop solution was then added to each well. In wells that contain cytokine, the color of the wells turned from blue to yellow upon addition of stop solution. Absorbance was immediately read on a BioTek Synergy H1 microplate reader under normal read speed and with a delay of 100 msec at 490 nm.

In analyzing the cytokine data, a standard curve was produced by plotting endpoint absorbance readout at 490 nm within wells containing standard against the known cytokine concentrations within each of the standard wells. The standard curves produced from each assay fit a linear regression, and the resulting model was utilized to quantify cytokine production in the unknown samples for that respective assay. Where dilutions were relevant, as in the case of the IL-8 assay, the calculated cytokine concentration was multiplied by the appropriate dilution factor.

	SUW402	SUW431	DMSO	Cells only
50 nM	< 3.5 pg/mL	4.4 pg/mL	< 3.5 pg/mL	< 3.5 pg/mL
500 nM	< 3.5 pg/mL	8.4 pg/mL		

Fig. S1. TNF-α ELISA assay. A549 cells untreated, treated with DMSO, or treated with SUW402 and SUW431 at 50 and 500 nM.

	SUW402	SUW431	DMSO	Cells only
50 nM	3352 pg/mL	3369 pg/mL	2796 pg/mL	2682 pg/mL
500 nM	3106 pg/mL	3232 pg/mL		

Fig. S2. IL-8 ELISA assay. A549 cells untreated, treated with DMSO, or treated with SUW402 and SUW431 at 50 and 500 nM.

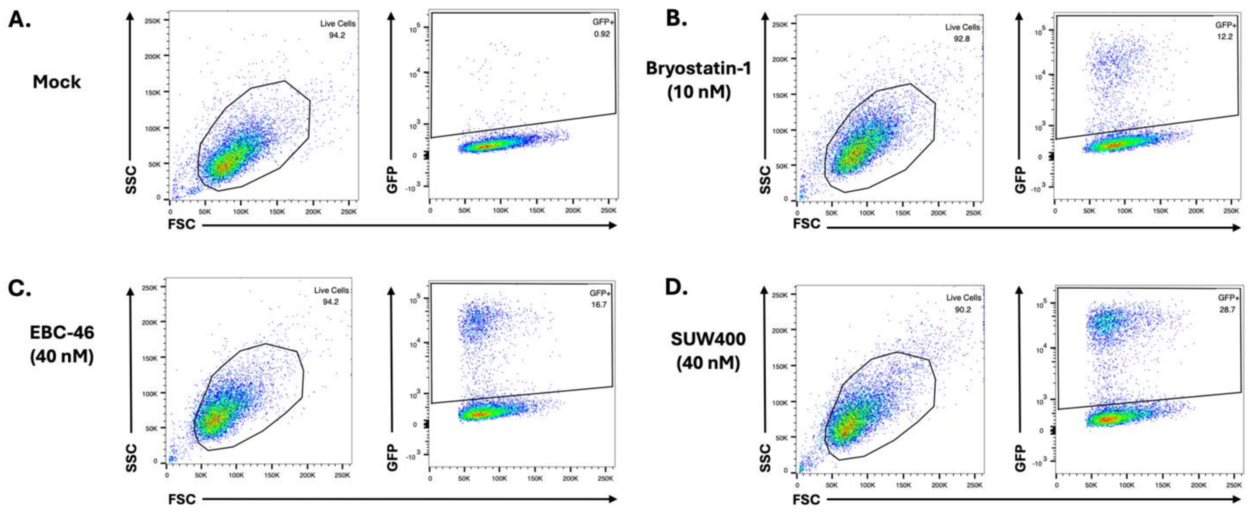

Fig. S3. Gating strategy. Representative flow cytometry plots with live cell gating (FSC vs. SSC) and GFP expression (FSC vs. GFP) of Jurkat-Latency (J-Lat) clone 10.6 cells. (**A**) Mock (untreated), (**B**) positive control bryostatin 1 (10 nM), (**C**) EBC-46 (40 nM), (**D**) SUW400 (40 nM).

Computational Methodologies

Using Schrödinger Maestro 13.5, the X-ray crystal structure of PDBu bound to the PKC-delta C1b domain (30, 32) was visualized (PDB ID: 7KNJ). Receptor grid generation, ligand structure generation, and docking were performed using Maestro 13.5 (PrepWizard, MacroModel, and Glide).

Ligand Docking Methodology for PKC-delta C1b and Tigliane-type Ligands

Protein preparation (PrepWizard):
7KNJ was imported into Maestro using "File" → "get PDB…". With the raw 7KNJ structure selected, the protein was prepared using the "Protein Preparation Workflow" function with the default settings found in Maestro 13.5 (OPLS4).

Receptor grid generation (Glide):
To make a grid box, the two additional phosphatidyl choline ligands found in this crystal structure were removed (one ligand maximum is allowed for grid generation). Using the "Receptor Grid Generation" function, with the prepared protein structure selected in the workspace, the receptor grid box was defined by selecting the PDBu ligand as the center of the grid box. Amino acid residues with rotatable hydroxyl/thiol groups in the receptor grid box were given the freedom to rotate (Y236, S240, T242, S249). All other settings were left as default.

Ligand preparation (MacroModel):
Using the "Conformational Search" function, structures of EBC-46 were generated by Torsional sampling (Monte Carlo Multiple Minimum). An implicit solvent model of water was used with the OPLS4 force field because of its more accurate hydrogen bond weighting. For minimization, PRCG was used with maximum iterations of 5000 with convergence on energy and a threshold of 0.001. All conformers within a 5.00 kcal energy window of the lowest energy structure (457 total conformers) were used in the ligand docking experiment.

Ligand docking (Glide):
Using the "Ligand Docking" function, the 7KNJ receptor grid box was selected as the .zip folder, and the ligands to be docked were imported from the .maegz file of the EBC-46 Monte Carlo conformational search (all conformers included). The precision was set to XP (extra precision), and the rest of the settings were left on the default parameters for XP docking. The docking pose with the highest docking score was used for comparison with the parent crystal structure of PDBu.

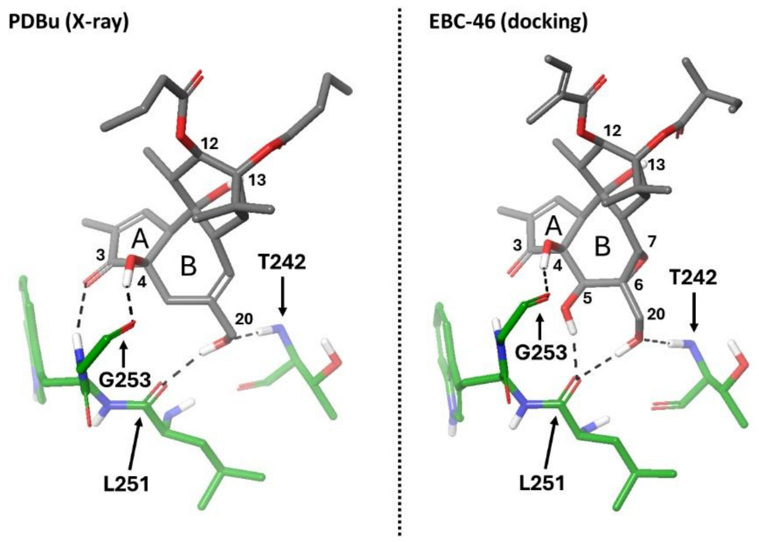

Fig. S4. Tigliane interactions with the PKC-δ-C1b domain. X-ray crystal structure of PDBu bound to the C1b domain of PKC-δ (*left*) and docking simulation of EBC-46 (*right*).

Phorbol Diester Pharmacophore Alignment

Generation of a phorbol pharmacophore hypothesis:
Using the above ligand docking procedure, the bound pose of phorbol 12,13-diacetate was calculated by performing a Monte Carlo search of its most stable conformers and docking them to the previously prepared receptor grid box. The best docking poses were exported for the generation of a pharmacophore model. Using the "Develop Pharmacophore Hypothesis" function in Maestro 13.5, two families of pharmacophores were generated – a "4-point model" (including the C3 oxygen, the C9 oxygen, and the C20-OH) and a "5-point model" (including the C4 alcohol as a hydrogen bond donor). Each model was then divided into three subgroups of varying tolerances (1 Å, 1.5 Å, and 2 Å) for a total of six pharmacophore hypotheses (**Fig. S5**).

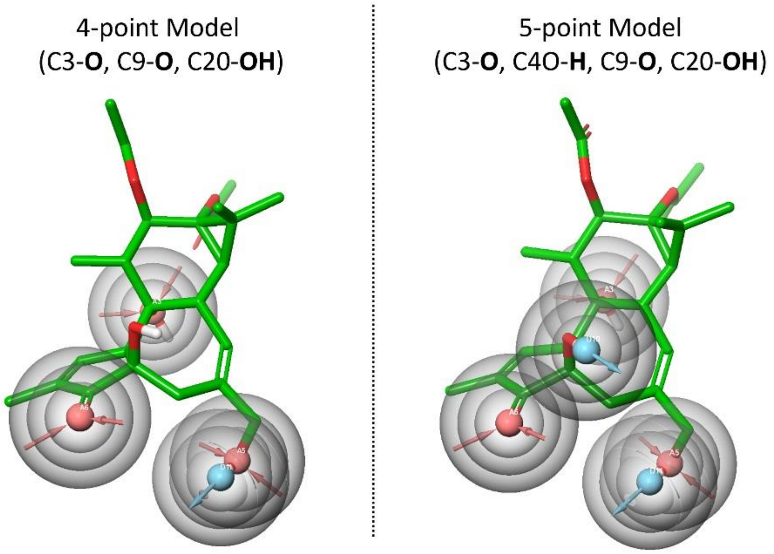

Fig. S5. Pharmacophore hypotheses. Pharmacophore model including C3 carbonyl, C20 alcohol, and C9 alcohol (*left*) and pharmacophore model also including the C4 alcohol (*right*).

Generation of Ligand Library for Screening:
ADMB was subjected to a Monte Carlo search as described above, and the results of this search were directly used in the following "Phase Ligand Screening" step. (S)-DAG was also subjected to a Monte Carlo search as described above, and the results of this conformational search for (S)-DAG were subjected to a further conformational screening using the "LigPrep" function (OPLS_2005, 32 conformers maximum). Both the results of the Monte Carlo search and the LigPrep search for (S)-DAG were used in the following "Phase Ligand Screening" step.

Phase Ligand Screening:
Using all six pharmacophore hypotheses, the previously described library of ADMB and (S)-DAG conformers were screened for their alignment with the phorbol pharmacophore using the "Phase Ligand Screening" function. All settings were left as default except the maximum number of hits to be returned per molecule was set to "3". The representative conformer for (S)-DAG was

generated by the 4-point model, while the representative conformer for ADMB was generated by the 5-point model. These conformers were selected from the highest scoring matches of the screening.

Alignment of Pharmacophoric Elements:

The PKC-bound conformer of PDBu was exported from the 7KNJ "prepared" X-ray crystal structure (previously described in the ligand docking procedure). The representative conformers for (S)-DAG and ADMB were then added to the workspace and aligned with the PDBu by first using the "Quick Align" function on all three molecules. Then the "Superposition" function was used to align the atom pairs of the C3, C9, and C20 oxygens with the respective pharmacophoric atoms on ADMB and (S)-DAG. The atoms of the PDBu structure were selected first, then aligned with the second compound. To align the third compound, its atoms were selected and then aligned to PDBu (the sequence of this alignment is important to superimpose all three structures in space). This superposition process also provided the RMSD analysis for the selected pharmacophoric elements. With all three molecules superimposed, the appropriate perspective was chosen, and the camera view was saved using the "Save camera view" function. The two aligned molecules were moved laterally, one at a time, using the "Move atoms" with shift + right click. The camera view was then restored with "Restore camera view" to show the three aligned 3D structures (**Fig. S6-A**). The lipid groups and the primary alcohols were then rotated manually with the "Move atoms" function for clarity of the alignment of pharmacophoric elements (**Fig. S6-B**).

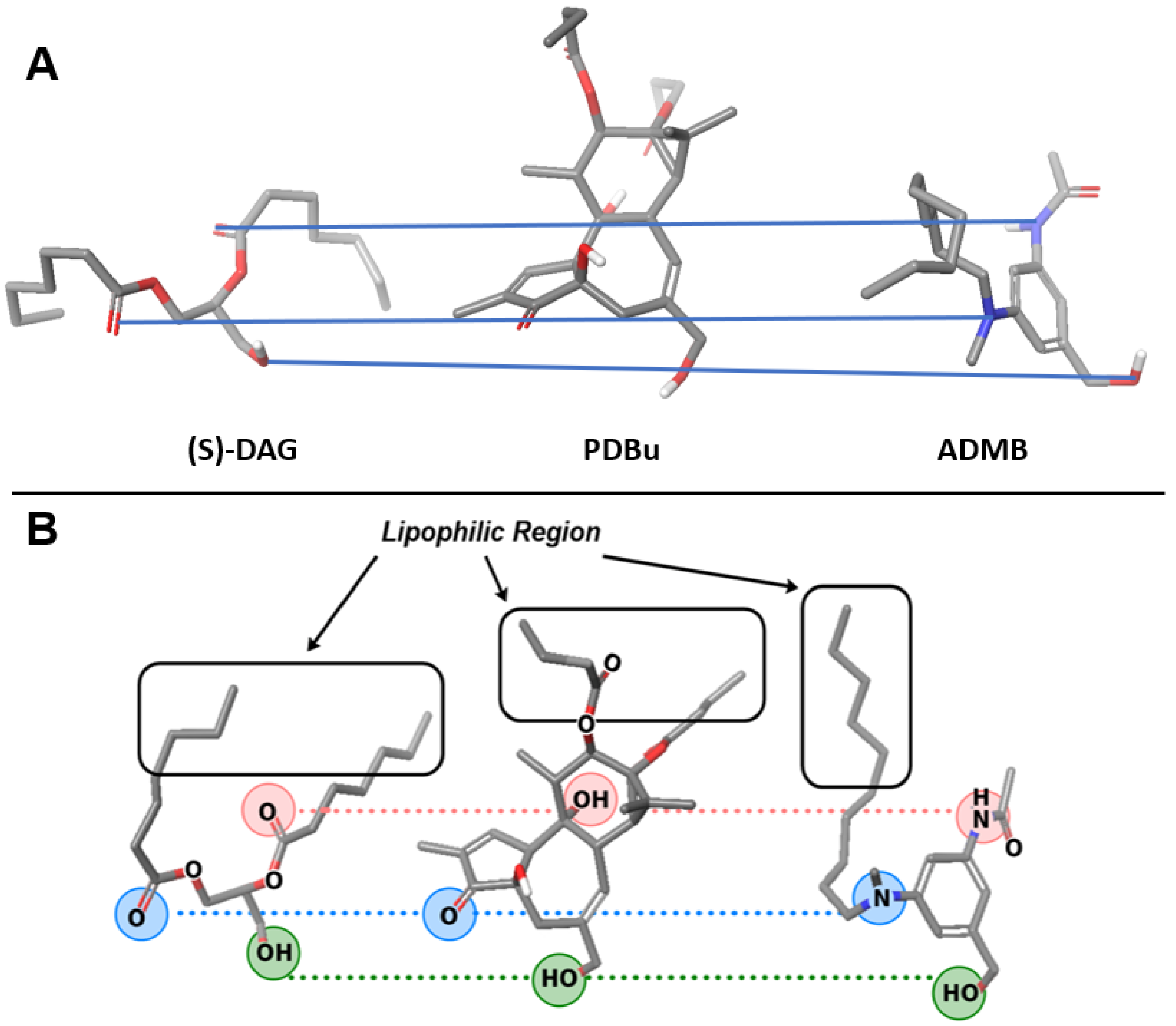

Fig. S6. **Pharmacophore alignment of (S)-DAG, PDBu, and ADMB**. (**A**) Raw structural alignment of (S)-DAG, PDBu, and ADMB, (**B**) Manually adjusted structural alignment of the three molecules with key atoms and pharmacophoric elements labeled.

Saturation Binding Experiment (PKC-β-I)	Saturation Binding Experiment (PKC-δ)

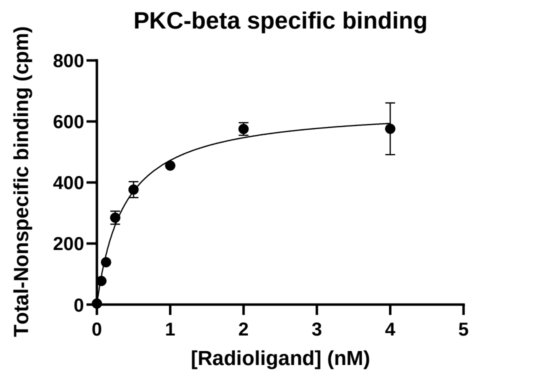

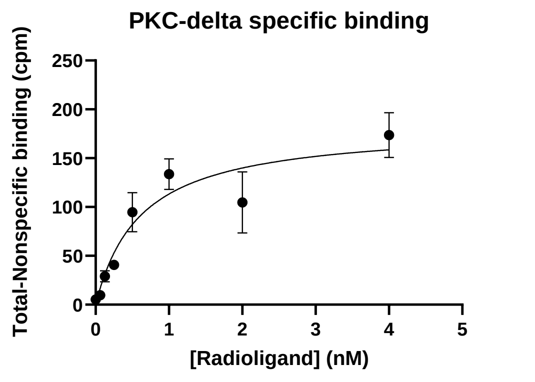

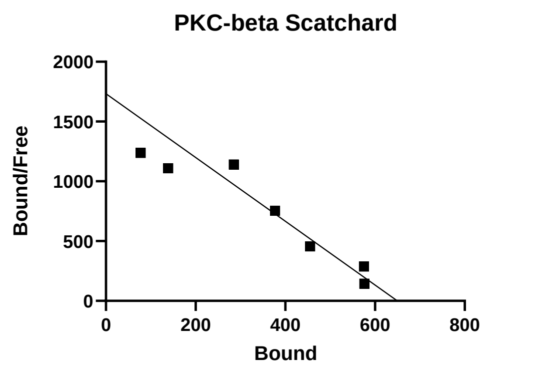

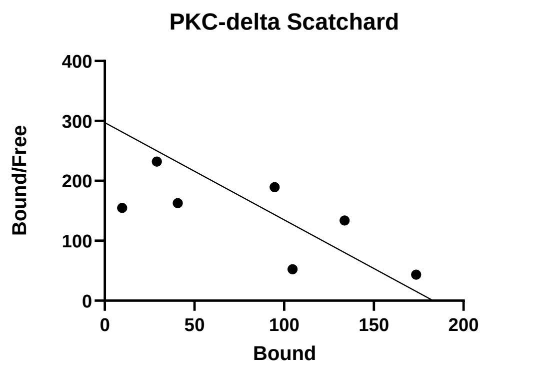

	PKC-β-I	PKC-δ
Best-fit values		
Bmax	649.6	183.0
Kd	0.3748	0.6158
95% CI (profile likelihood)		
Bmax	597.4 to 707.5	131.8 to 276.3
Kd	0.2818 to 0.4973	0.2223 to 1.865
Goodness of Fit		
Degrees of Freedom	6	6
R squared	0.9916	0.9151
Sum of Squares	2895	2279
Sy.x	21.97	19.49
Number of points		
# of X values	8	8
# Y values analyzed	8	8

Fig. S7. K_d determination of 3H-PDBU for PKC-β-I and PKC-δ.

EBC-46 (PKC-β-I)

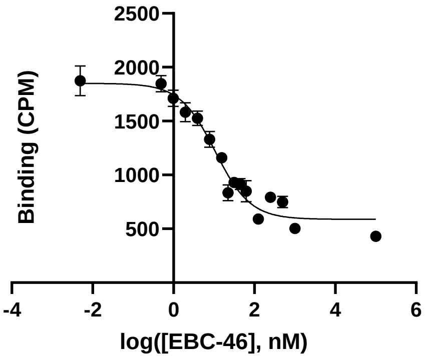

EBC-46 (PKC-δ)

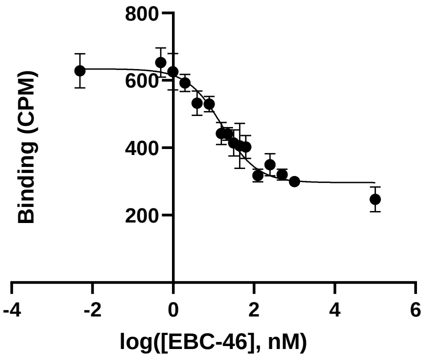

	EBC-46 (PKC-β-I)	EBC-46 (PKC-δ)
Best-fit values		
logKi	0.1397	0.5102
HotNM	= 2.500	= 2.500
HotKdNM	= 0.3700	= 0.6200
Top	1850	634.1
Bottom	587.9	296.6
Ki	1.380	3.237
95% CI (profile likelihood)		
logKi	0.0009304 to 0.2751	0.3231 to 0.6916
Top	1771 to 1932	608.2 to 661.5
Bottom	524.4 to 649.3	271.8 to 320.3
Ki	1.002 to 1.884	2.104 to 4.916
Goodness of Fit		
Degrees of Freedom	45	45
R squared	0.9472	0.9129
Sum of Squares	558529	69391
Sy.x	111.4	39.27
Constraints		
HotNM	HotNM = 2.5	HotNM = 2.5
HotKdNM	HotKdNM = 0.37	HotKdNM = 0.62
Number of points		
# of X values	48	48
# Y values analyzed	48	48

Fig. S8. K_i determination for EBC-46 for PKC-β-I and PKC-δ.

SUW400 (PKC-β-I)		SUW400 (PKC-δ)
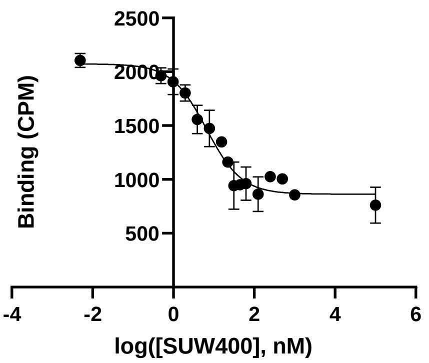		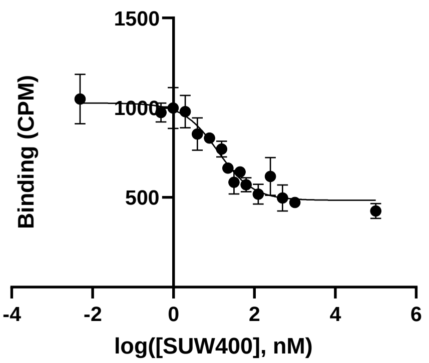
Best-fit values		
logKi	-0.07019	0.4034
HotNM	= 2.500	= 2.500
HotKdNM	= 0.3700	= 0.6200
Top	2073	1026
Bottom	863.6	484.0
Ki	0.8508	2.532
95% CI (profile likelihood)		
logKi	-0.2348 to 0.08993	0.1962 to 0.6027
Top	1977 to 2176	976.7 to 1078
Bottom	799.0 to 925.8	440.8 to 525.1
Ki	0.5823 to 1.230	1.571 to 4.006
Goodness of Fit		
Degrees of Freedom	45	45
R squared	0.9264	0.8844
Sum of Squares	699688	243564
Sy.x	124.7	73.57
Constraints		
HotNM	HotNM = 2.5	HotNM = 2.5
HotKdNM	HotKdNM = 0.37	HotKdNM = 0.62
Number of points		
# of X values	48	48
# Y values analyzed	48	48

Fig. S9. K_i determination for SUW400 for PKC-β-I and PKC-δ.

SUW402 (PKC-β-I)		SUW402 (PKC-δ)*

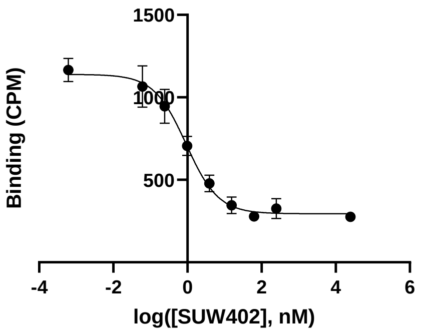

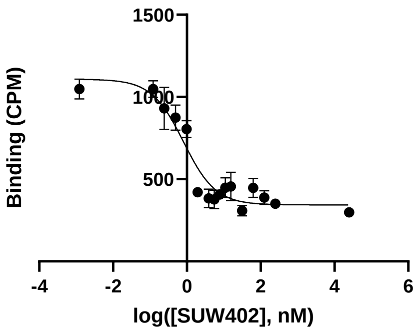

Best-fit values		
logKi	-0.9263	-0.3561
HotNM	= 2.500	= 2.500
HotKdNM	= 0.3700	= 2.940
Top	1139	1108
Bottom	294.3	343.4
Ki	0.1185	0.4404*
95% CI (profile likelihood)		
logKi	-1.092 to -0.7595	-0.5188 to -0.1906
Top	1082 to 1199	1032 to 1187
Bottom	253.6 to 334.3	304.7 to 381.3
Ki	0.08100 to 0.1740	0.3028 to 0.6447
Goodness of Fit		
Degrees of Freedom	24	45
R squared	0.9686	0.8991
Sum of Squares	99569	351048
Sy.x	64.41	88.32
Constraints		
HotNM	HotNM = 2.5	HotNM = 2.5
HotKdNM	HotKdNM = 0.37	HotKdNM = 2.94
Number of points		
# of X values	27	48
# Y values analyzed	27	48

Fig. S10. K_i determination for SUW402 for PKC-β-I and PKC-δ. *PKC-δ used in this experiment was from a different vendor - human PKC-δ-GST fusion protein (Sinobiological)

SUW403 (PKC-β-I)

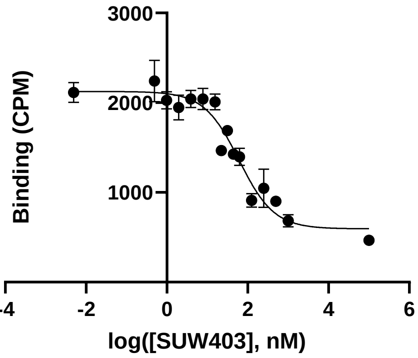

SUW403 (PKC-δ)

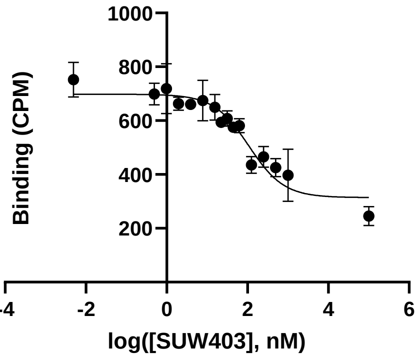

	SUW403 (PKC-β-I)	SUW403 (PKC-δ)
Best-fit values		
logKi	0.8975	1.330
HotNM	= 2.500	= 2.500
HotKdNM	= 0.3700	= 0.6200
Top	2125	698.1
Bottom	594.1	314.2
Ki	7.898	21.36
95% CI (profile likelihood)		
logKi	0.7465 to 1.054	1.071 to 1.611
Top	2040 to 2211	669.6 to 727.9
Bottom	468.9 to 712.7	257.8 to 363.4
Ki	5.579 to 11.32	11.77 to 40.79
Goodness of Fit		
Degrees of Freedom	45	45
R squared	0.9240	0.8547
Sum of Squares	1154535	139027
Sy.x	160.2	55.58
Constraints		
HotNM	HotNM = 2.5	HotNM = 2.5
HotKdNM	HotKdNM = 0.37	HotKdNM = 0.62
Number of points		
# of X values	48	48
# Y values analyzed	48	48

Fig. S11. K_i determination for SUW403 for PKC-β-I and PKC-δ.

SUW406 (PKC-β-I)

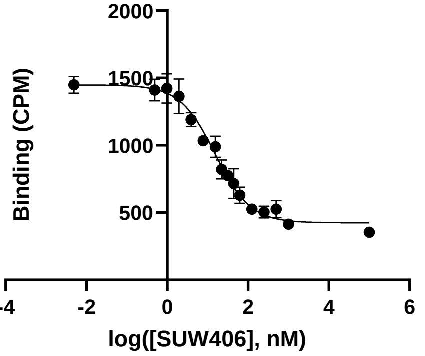

SUW406 (PKC-δ)

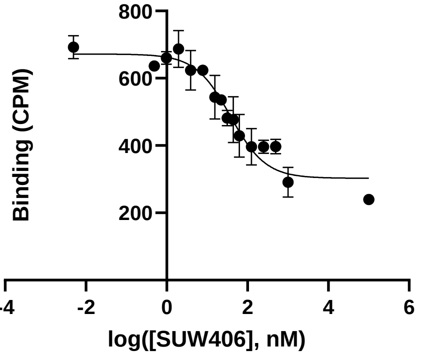

	SUW406 (PKC-β-I)	SUW406 (PKC-δ)
Best-fit values		
logKi	0.2968	0.8752
HotNM	= 2.500	= 2.500
HotKdNM	= 0.3700	= 0.6200
Top	1449	671.9
Bottom	424.2	302.8
Ki	1.981	7.503
95% CI (profile likelihood)		
logKi	0.1910 to 0.4003	0.6843 to 1.073
Top	1401 to 1497	644.6 to 700.1
Bottom	380.8 to 466.5	268.1 to 335.3
Ki	1.553 to 2.514	4.834 to 11.83
Goodness of Fit		
Degrees of Freedom	45	45
R squared	0.9663	0.8937
Sum of Squares	233068	101122
Sy.x	71.97	47.40
Constraints		
HotNM	HotNM = 2.5	HotNM = 2.5
HotKdNM	HotKdNM = 0.37	HotKdNM = 0.62
Number of points		
# of X values	48	48
# Y values analyzed	48	48

Fig. S12. K_i determination for SUW406 for PKC-β-I and PKC-δ.

SUW407 (PKC-β-I)		SUW407 (PKC-δ)
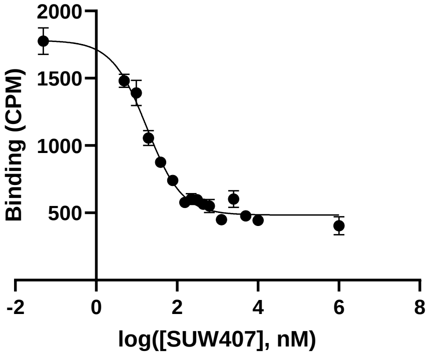		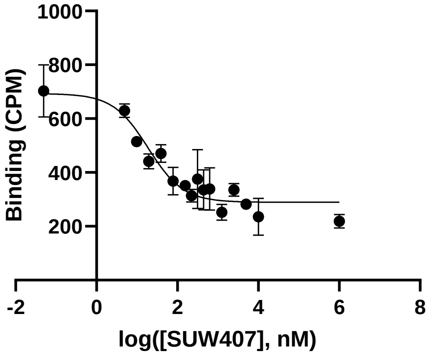
<u>Best-fit values</u>		
logKi	0.3637	0.5722
HotNM	= 2.500	= 2.500
HotKdNM	= 0.3700	= 0.6200
Top	1781	693.2
Bottom	483.8	289.3
Ki	2.311	3.734
<u>95% CI (profile likelihood)</u>		
logKi	0.2818 to 0.4482	0.3011 to 0.8939
Top	1713 to 1850	626.3 to 763.6
Bottom	456.7 to 510.6	261.9 to 314.9
Ki	1.913 to 2.807	2.001 to 7.833
<u>Goodness of Fit</u>		
Degrees of Freedom	45	45
R squared	0.9759	0.8198
Sum of Squares	192478	168142
Sy.x	65.40	61.13
<u>Constraints</u>		
HotNM	HotNM = 2.5	HotNM = 2.5
HotKdNM	HotKdNM = 0.37	HotKdNM = 0.62
<u>Number of points</u>		
# of X values	48	48
# Y values analyzed	48	48

Fig. S13. K_i determination for SUW407 for PKC-β-I and PKC-δ.

SUW413 (PKC-β-I)

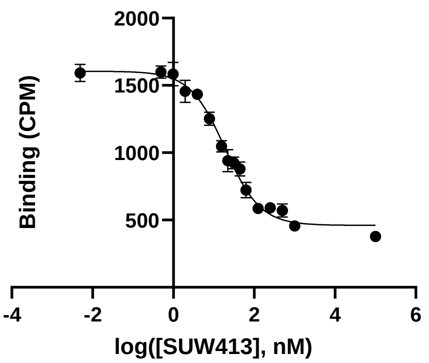

SUW413 (PKC-δ)

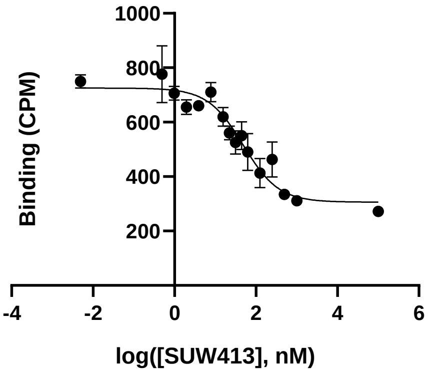

Best-fit values		
logKi	0.3892	0.9851
HotNM	= 2.500	= 2.500
HotKdNM	= 0.3700	= 0.6200
Top	1605	725.2
Bottom	460.4	306.1
Ki	2.450	9.664
95% CI (profile likelihood)		
logKi	0.3087 to 0.4687	0.7865 to 1.191
Top	1565 to 1646	695.0 to 756.4
Bottom	421.2 to 498.8	264.1 to 345.4
Ki	2.036 to 2.943	6.116 to 15.52
Goodness of Fit		
Degrees of Freedom	45	45
R squared	0.9790	0.8912
Sum of Squares	179575	131271
Sy.x	63.17	54.01
Constraints		
HotNM	HotNM = 2.5	HotNM = 2.5
HotKdNM	HotKdNM = 0.37	HotKdNM = 0.62
Number of points		
# of X values	48	48
# Y values analyzed	48	48

Fig. S14. K_i determination for SUW413 for PKC-β-I and PKC-δ.

	SUW421 (PKC-β-I)	SUW421 (PKC-δ)
	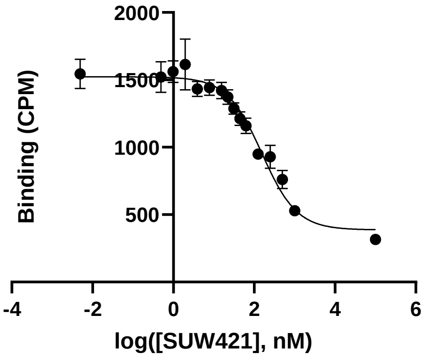	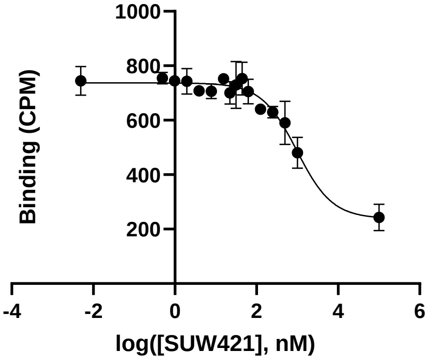
<u>Best-fit values</u>		
logKi	1.302	2.288
HotNM	= 2.500	= 2.500
HotKdNM	= 0.3700	= 0.6200
Top	1522	736.9
Bottom	386.6	238.5
Ki	20.05	194.1
<u>95% CI (profile likelihood)</u>		
logKi	1.152 to 1.452	2.133 to 2.444
Top	1478 to 1568	720.6 to 753.4
Bottom	291.1 to 476.4	185.7 to 290.8
Ki	14.20 to 28.31	135.9 to 277.9
<u>Goodness of Fit</u>		
Degrees of Freedom	45	45
R squared	0.9464	0.9006
Sum of Squares	378691	88078
Sy.x	91.74	44.24
<u>Constraints</u>		
HotNM	HotNM = 2.5	HotNM = 2.5
HotKdNM	HotKdNM = 0.37	HotKdNM = 0.62
<u>Number of points</u>		
# of X values	48	48
# Y values analyzed	48	48

Fig. S15. K_i determination for SUW421 for PKC-β-I and PKC-δ.

SUW422 (PKC-β-I)

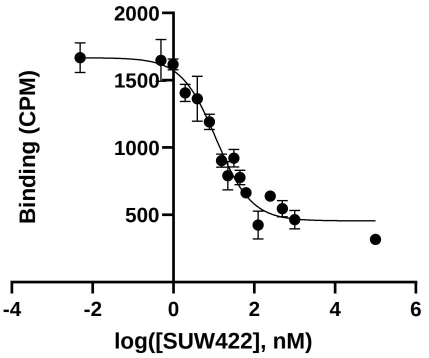

SUW422 (PKC-δ)

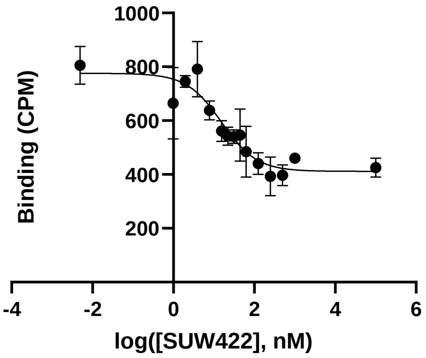

	SUW422 (PKC-β-I)	SUW422 (PKC-δ)
Best-fit values		
logKi	0.1731	0.4832
HotNM	= 2.500	= 2.500
HotKdNM	= 0.3700	= 0.6200
Top	1667	775.9
Bottom	455.4	413.9
Ki	1.490	3.042
95% CI (profile likelihood)		
logKi	0.03125 to 0.3113	0.1889 to 0.7678
Top	1591 to 1747	724.1 to 832.3
Bottom	391.9 to 516.8	368.9 to 456.1
Ki	1.075 to 2.048	1.545 to 5.859
Goodness of Fit		
Degrees of Freedom	45	40
R squared	0.9448	0.7707
Sum of Squares	541788	204802
Sy.x	109.7	71.55
Constraints		
HotNM	HotNM = 2.5	HotNM = 2.5
HotKdNM	HotKdNM = 0.37	HotKdNM = 0.62
Number of points		
# of X values	48	45
# Y values analyzed	48	43

Fig. S16. K_i determination for SUW422 for PKC-β-I and PKC-δ.

SUW424 (PKC-β-I)	SUW424 (PKC-δ)
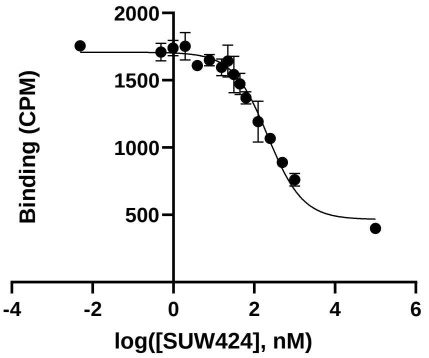	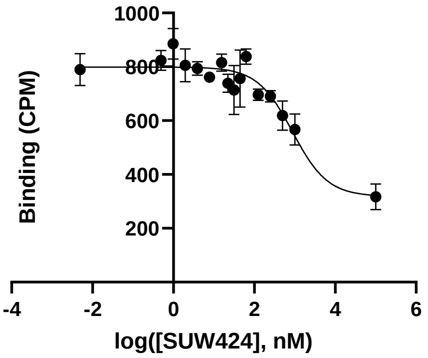

	SUW424 (PKC-β-I)	SUW424 (PKC-δ)
Best-fit values		
logKi	1.445	2.242
HotNM	= 2.500	= 2.500
HotKdNM	= 0.3700	= 0.6200
Top	1708	799.0
Bottom	465.6	318.1
Ki	27.85	174.5
95% CI (profile likelihood)		
logKi	1.325 to 1.562	2.011 to 2.469
Top	1671 to 1746	776.4 to 822.0
Bottom	377.9 to 549.8	245.4 to 390.2
Ki	21.16 to 36.50	102.5 to 294.2
Goodness of Fit		
Degrees of Freedom	45	45
R squared	0.9613	0.8226
Sum of Squares	298833	164354
Sy.x	81.49	60.43
Constraints		
HotNM	HotNM = 2.5	HotNM = 2.5
HotKdNM	HotKdNM = 0.37	HotKdNM = 0.62
Number of points		
# of X values	48	48
# Y values analyzed	48	48

Fig. S17. K_i determination for SUW424 for PKC-β-I and PKC-δ.

SUW425 (PKC-β-I)		SUW425 (PKC-δ)
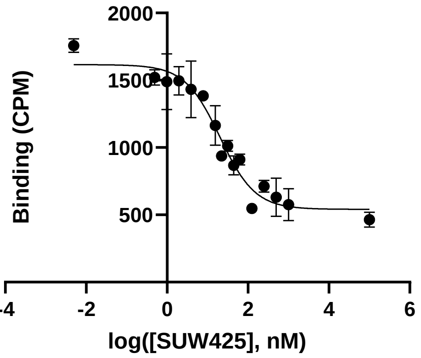		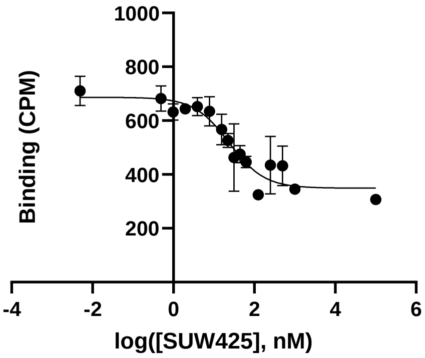
<u>Best-fit values</u>		
logKi	0.4165	0.6748
HotNM	= 2.500	= 2.500
HotKdNM	= 0.3700	= 0.6200
Top	1616	686.7
Bottom	541.0	349.2
Ki	2.609	4.730
<u>95% CI (profile likelihood)</u>		
logKi	0.2542 to 0.5744	0.4349 to 0.9095
Top	1542 to 1693	651.7 to 723.4
Bottom	465.2 to 613.9	311.2 to 385.1
Ki	1.796 to 3.753	2.722 to 8.120
<u>Goodness of Fit</u>		
Degrees of Freedom	45	45
R squared	0.9188	0.8200
Sum of Squares	651952	159246
Sy.x	120.4	59.49
<u>Constraints</u>		
HotNM	HotNM = 2.5	HotNM = 2.5
HotKdNM	HotKdNM = 0.37	HotKdNM = 0.62
<u>Number of points</u>		
# of X values	48	48
# Y values analyzed	48	48

Fig. S18. K_i determination for SUW425 for PKC-β-I and PKC-δ.

SUW426 (PKC-β-I)		SUW426 (PKC-δ)
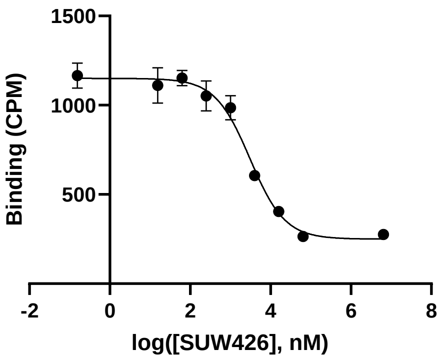		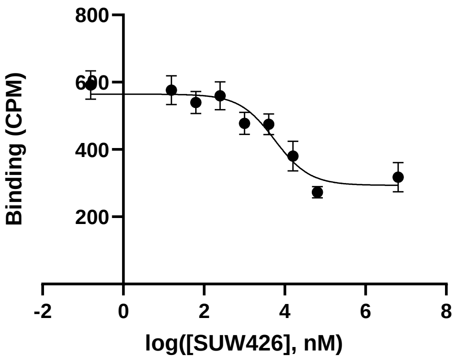
<u>Best-fit values</u>		
logKi	2.598	2.846
HotNM	= 2.500	= 5.000
HotKdNM	= 0.3700	= 0.7200
Top	1150	564.4
Bottom	249.5	293.5
Ki	396.4	701.8
<u>95% CI (profile likelihood)</u>		
logKi	2.466 to 2.729	2.459 to 3.181
Top	1113 to 1188	538.4 to 591.8
Bottom	198.9 to 298.9	251.0 to 333.7
Ki	292.7 to 535.8	287.7 to 1517
<u>Goodness of Fit</u>		
Degrees of Freedom	24	24
R squared	0.9767	0.8716
Sum of Squares	85431	45215
Sy.x	59.66	43.40
<u>Constraints</u>		
HotNM	HotNM = 2.5	HotNM = 5
HotKdNM	HotKdNM = 0.37	HotKdNM = 0.72
<u>Number of points</u>		
# of X values	27	27
# Y values analyzed	27	27

Fig. S19. K_i determination for SUW426 for PKC-β-I and PKC-δ.

	SUW428 (PKC-β-I)	SUW428 (PKC-δ)

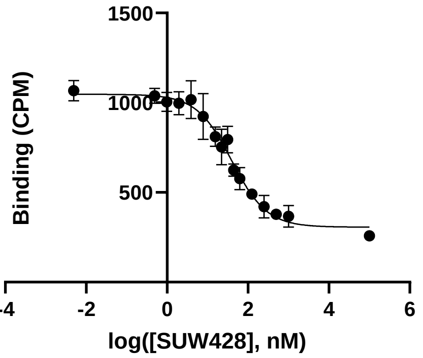

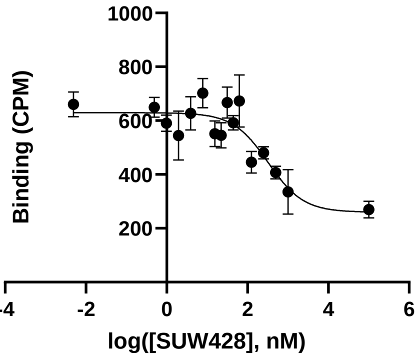

	SUW428 (PKC-β-I)	SUW428 (PKC-δ)
Best-fit values		
logKi	0.7091	1.808
HotNM	= 2.500	= 2.500
HotKdNM	= 0.3700	= 0.6200
Top	1047	629.5
Bottom	306.5	259.8
Ki	5.118	64.20
95% CI (profile likelihood)		
logKi	0.5868 to 0.8321	1.496 to 2.101
Top	1011 to 1084	600.4 to 659.7
Bottom	261.0 to 350.4	181.5 to 332.4
Ki	3.862 to 6.794	31.35 to 126.3
Goodness of Fit		
Degrees of Freedom	45	45
R squared	0.9481	0.7162
Sum of Squares	186767	235024
Sy.x	64.42	72.27
Constraints		
HotNM	HotNM = 2.5	HotNM = 2.5
HotKdNM	HotKdNM = 0.37	HotKdNM = 0.62
Number of points		
# of X values	48	48
# Y values analyzed	48	48

Fig. S20. K_i determination for SUW428 for PKC-β-I and PKC-δ.

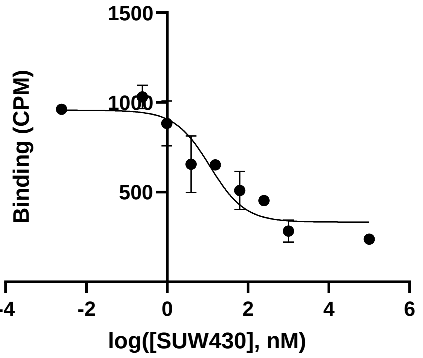

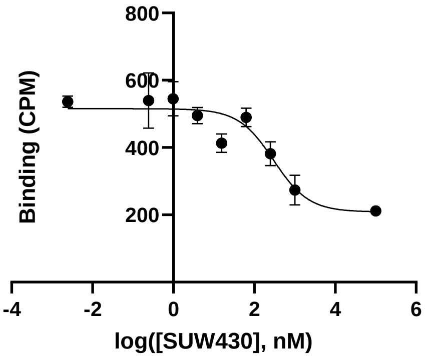

	SUW430 (PKC-β-I)	SUW430 (PKC-δ)
Best-fit values		
logKi	0.1684	1.776
HotNM	= 2.500	= 2.500
HotKdNM	= 0.3700	= 0.6200
Top	956.6	515.4
Bottom	333.3	208.7
Ki	1.474	59.71
95% CI (profile likelihood)		
logKi	-0.3875 to 0.7769	1.391 to 2.113
Top	860.9 to 1065	488.0 to 543.7
Bottom	232.1 to 419.8	150.4 to 264.3
Ki	0.4097 to 5.983	24.62 to 129.6
Goodness of Fit		
Degrees of Freedom	24	24
R squared	0.8564	0.8401
Sum of Squares	299708	61934
Sy.x	111.7	50.80
Constraints		
HotNM	HotNM = 2.5	HotNM = 2.5
HotKdNM	HotKdNM = 0.37	HotKdNM = 0.62
Number of points		
# of X values	27	27
# Y values analyzed	27	27

Fig. S21. K_i determination for SUW430 for PKC-β-I and PKC-δ.

	SUW431 (PKC-β-I)	SUW431 (PKC-δ)

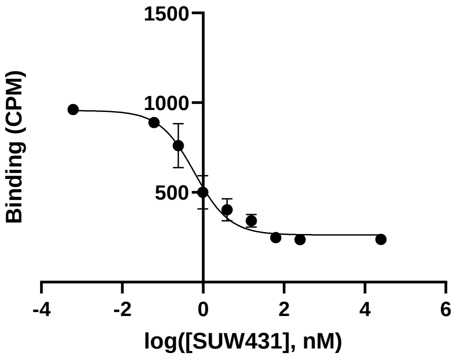

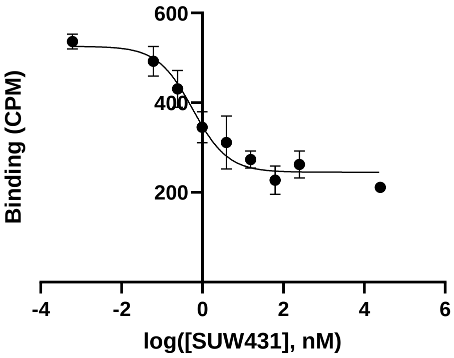

	SUW431 (PKC-β-I)	SUW431 (PKC-δ)
Best-fit values		
logKi	-1.102	-0.9424
HotNM	= 2.500	= 2.500
HotKdNM	= 0.3700	= 0.6200
Top	956.3	525.6
Bottom	262.9	244.9
Ki	0.07904	0.1142
95% CI (profile likelihood)		
logKi	-1.289 to -0.9069	-1.237 to -0.6262
Top	899.0 to 1016	490.7 to 562.8
Bottom	226.1 to 298.9	222.8 to 266.3
Ki	0.05135 to 0.1239	0.05796 to 0.2365
Goodness of Fit		
Degrees of Freedom	24	24
R squared	0.9577	0.9136
Sum of Squares	87274	30403
Sy.x	60.30	35.59
Constraints		
HotNM	HotNM = 2.5	HotNM = 2.5
HotKdNM	HotKdNM = 0.37	HotKdNM = 0.62
Number of points		
# of X values	27	27
# Y values analyzed	27	27

Fig. S22. K_i determination for SUW431 for PKC-β-I and PKC-δ.
